# New taxa of freshwater mussels (Unionidae) from a species-rich but overlooked evolutionary hotspot in Southeast Asia

**DOI:** 10.1038/s41598-017-11957-9

**Published:** 2017-09-14

**Authors:** Ivan N. Bolotov, Ilya V. Vikhrev, Alexander V. Kondakov, Ekaterina S. Konopleva, Mikhail Yu. Gofarov, Olga V. Aksenova, Sakboworn Tumpeesuwan

**Affiliations:** 1 Northern Arctic Federal University, Arkhangelsk, Russian Federation; 2 Federal Center for Integrated Arctic Research, Russian Academy of Sciences, Arkhangelsk, Russian Federation; 3 Department of Biology, Faculty of Science, Mahasarakham University, Maha Sarakham, Thailand

## Abstract

Southeast Asia harbors a unique and diverse freshwater fauna of Mesozoic origin, which is under severe threat of extinction because of rapid economic development and urbanization. The largest freshwater basins of the region are certainly the primary evolutionary hotspots and they attract the most attention as key biodiversity areas for conservation. In contrast, medium-sized rivers are considered low-importance areas with secondary biodiversity, whose faunas originated via founder events from larger basins during the Pleistocene, although such a scenario has never been tested by using a phylogenetic approach. In this investigation, we used freshwater mussels (Unionidae) as a model to estimate the levels of endemism within the Sittaung, a little-known remote basin in Myanmar, compared with the surrounding larger rivers (Irrawaddy, Salween and Mekong). We discovered that the Sittaung represents an exceptional evolutionary hotspot with numerous endemic taxa of freshwater mussels. On the basis of our extensive dataset, we describe two new tribes, two genera, seven species and a subspecies of Unionidae. Our results highlight that medium-sized basins may represent separate evolutionary hotspots that harbor a number of endemic lineages. These basins should therefore be a focus of special conservation efforts alongside the largest Southeast Asian rivers.

## Introduction

In the modern period of the sixth mass extinction (Anthropocene), freshwater biodiversity is under severe threat because of increasing anthropogenic pressure, which leads to habitat degradation, water pollution, keystone species declines, and the homogenization of faunas^1–6^. Climate change may increase the effects of human impacts, especially for taxa with low abundance and restricted ranges, and may trigger multiple local extinctions^7, 8^. Our understanding of spatial biodiversity patterns across freshwater basins is very limited because the systematics of many groups are not developed, including the important invertebrates such as bivalves and gastropods^9–13^. In Southeast Asia, the lack of reliable taxonomic information precludes producing the national maps of freshwater biodiversity hotspots, which is a task of great importance for conservation planning^9^.

Here, we use mussels in the family Unionidae, or naiads, as a model group for the assessment of spatial patterns of freshwater biodiversity across western Indo-China. This is the most species-rich bivalve family, with ~620–680 extant species^14–17^. The Unionidae most likely originated in Southeast and East Asia in the Jurassic, with subsequent expansions into other landmasses^9^. In several major Asian river systems (e.g., Mekong and Yangtze), exceptional intra-basin radiations of the Unionidae were discovered, which suggests that these basins may be considered ancient (long-lived) rivers that have existed throughout the Cenozoic^9, 18^. However, the freshwater mussel faunas of Asia have attracted little attention from scientists compared with those from Europe and North America^10, 17^. Although the importance of freshwater mussels in tropical ecosystems is still poorly known, they could play an essential role as biofilters in polluted water bodies^19^. Several species are successful invaders, and have spread beyond their native ranges together with the introduction of their host fishes and may threaten native communities^20, 21^. Finally, freshwater mussels are important objects for the ornamental pet trade, pearl cultivation and food markets across Asian countries^22–24^.

The taxonomy of freshwater mussels of Southeast Asia is complicated, because many nominal taxa were described from this region on the basis of conchological features, including small differences in shell shape^9, 10, 21, 25^. In western Indo-China, most historical samples were collected from the Irrawaddy, Salween, Pegu, Tavoy and Great Tenasserim river catchments^10, 24–48^. In contrast, nominal taxa from the Sittaung, a medium-sized river basin in Myanmar, were not described. In the 1880s, Leonardo Fea, an Italian naturalist and traveler, collected mussels from several tributaries of the Sittaung^49^. Fea’s collection was deposited in the Museo Civico di Storia Naturale di Genova (MSNG, Genoa, Italy). Since then, none of the freshwater mussels have been collected from the basin. Moreover, for some enigmatic reason Fea’s samples from the Sittaung were not used for the description of any unionid taxon^39, 40, 43, 50^.

From the comprehensive phylogenetic study of the Unionidae across the primary basins of the Oriental Region, Bolotov *et al*.^9^ showed that each large river system of this region is a separate evolutionary hotspot harboring a unique endemic naiad fauna. In the present investigation, we expand this sample to estimate the levels of endemism within the Sittaung compared with the surrounding larger rivers (Irrawaddy, Salween, and Mekong). From the phylogenetic and morphological studies of the mussel samples, we describe seven new species and a single subspecies, which are ancient endemic lineages of the Sittaung. Additionally, we found two new tribes and two new genera, which are described herein. Our findings highlight that the medium-sized basins may represent separate evolutionary hotspots that should be considered in future conservation planning for freshwater biodiversity in Southeast Asia.

## Results

### Phylogenetic and species delimitation analyses

**Figure 1:**
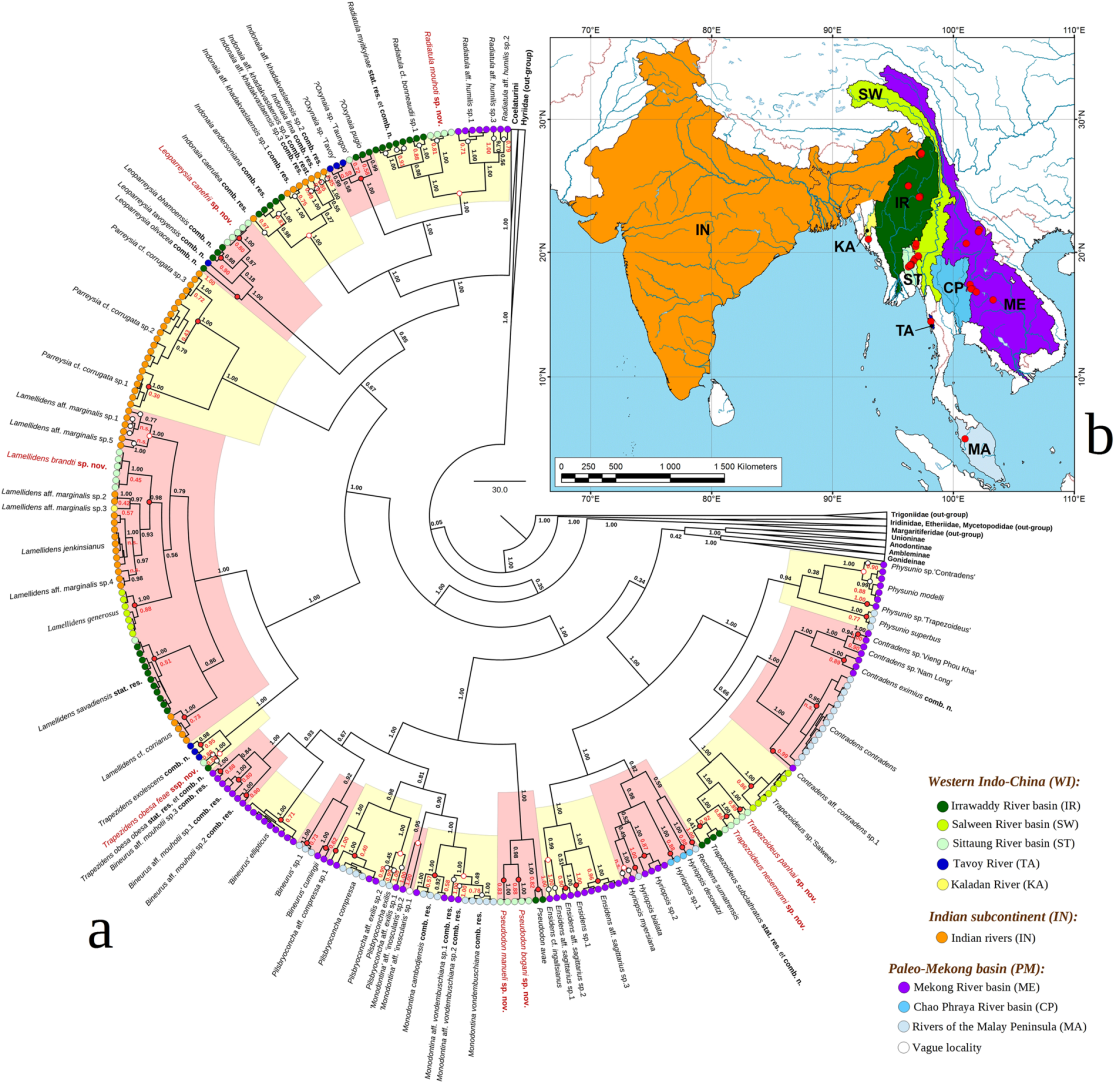
Haplotype-level phylogeny and distribution of Indo-Chinese Unionidae. (**a**) Multi-locus fossil-calibrated phylogeny based on the BEAST 1.8.4 model and obtained for the complete data set of mitochondrial and nuclear sequences (five partitions: three codons of COI + 16S rRNA + 28S rDNA). Color circles correspond to the distribution range of each haplotype. Black numbers near branches are Bayesian posterior probabilities. Red numbers near terminal nodes are support values for each prospective species (MOTU) based on the bPTP model of Zhang *et al*.^78^ (“n.s.” indicates that this MOTU was not supported). Solid red circles on the nodes indicate clades supported by both the sPTP (*p* < 0.001) and the mPTP models of Kapli *et al*.^79^ Empty black circles on the nodes indicate clades supported by the sPTP model of Kapli *et al*.^79^ (*p* < 0.001). Empty red circles on the nodes indicate clades supported by the mPTP of Kapli *et al*.^79^ The non-Indo-Chinese clades were collapsed. The list of sequences is presented in Supplementary Table 1. (**b**) Map of distribution areas. Red circles indicate our collecting localities (Supplementary Table 2). The map was created using ESRI ArcGIS 10 software (www.esri.com/arcgis); the topographic base of the map was created with ESRI Data and Maps.

Our multi-locus phylogeny (COI + 16S rRNA + 28S rDNA) contains 469 specimens of Unionidae, including 403 specimens from the Oriental Region (Supplementary Tables 1 and 2, Supplementary Figs 1 and 2). The Oriental sample includes 256 unique haplotypes belonging to 80 putative species (Fig. 1). The Bayesian Poisson tree processes (bPTP) model supports the majority of these possible species-level units (Fig. 1). The results inferred from the single-rate PTP (sPTP) model were generally similar to that of bPTP model, with a few exceptions (Fig. 1). The molecular operational taxonomic units (MOTUs) of the multi-rate PTP (mPTP) model were also comparable to those of the two other models, but in some cases, the mPTP revealed larger clusters, which occasionally joined all of the species within a certain genus (e.g., *Indonaia* and *Radiatula*) into a single MOTU (Fig. 1).

**Figure 2:**
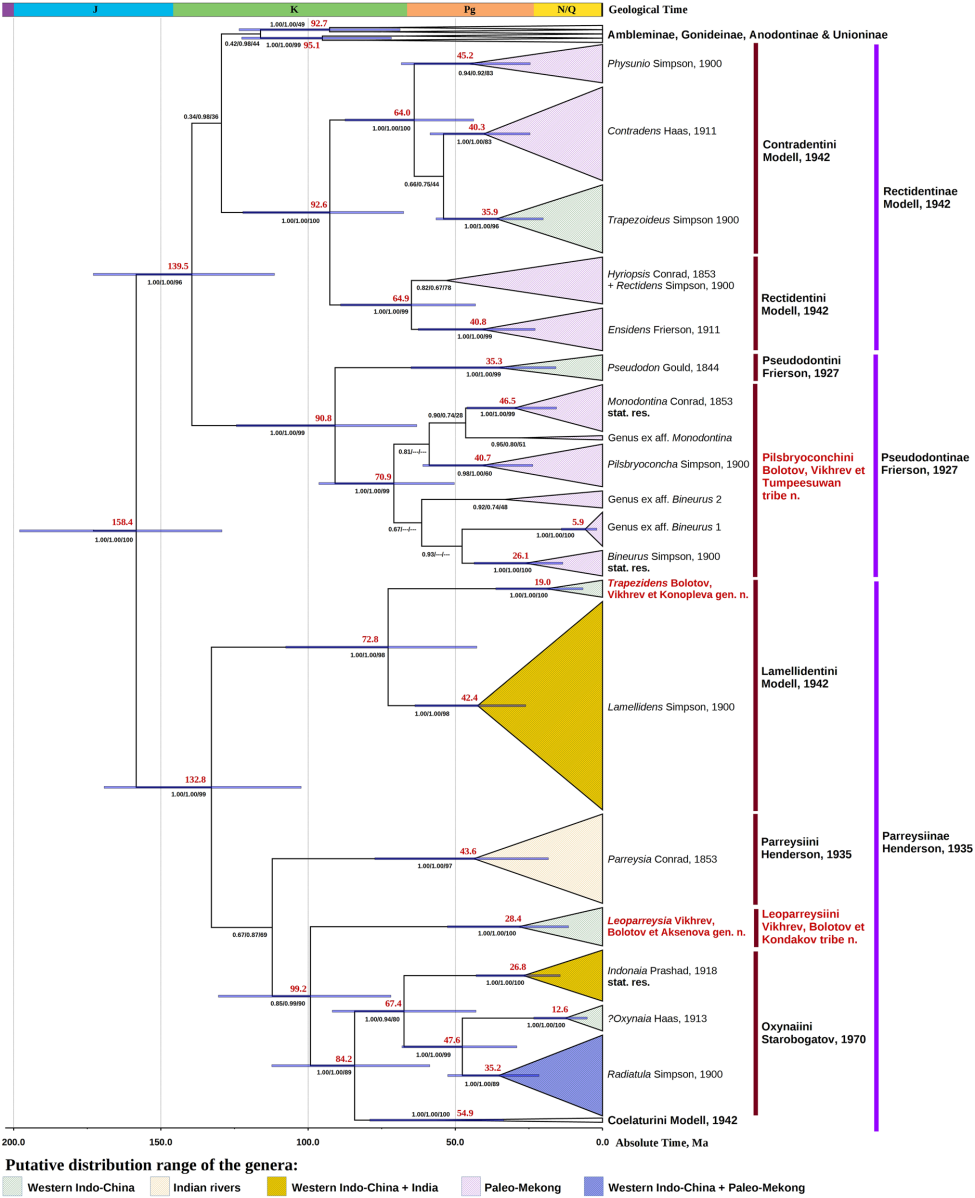
Generic revision of the Oriental Unionidae. Fossil-calibrated ultrametric chronogram calculated under a lognormal relaxed clock model and a Yule process speciation implemented in BEAST 1.8.4 and obtained for the complete data set of mitochondrial and nuclear sequences (five partitions: three codons of COI + 16S rRNA + 28S rDNA). Bars indicate 95% confidence intervals of the estimated divergence times between lineages (Ma). Black numbers near nodes are BPP values inferred from BEAST/BPP values inferred from MrBayes/BS values inferred from RAxML (“–”indicates a topological difference). Red numbers near nodes are mean ages (Ma). The timing of weakly supported nodes (MrBayes’s BPP < 0.90 and RAxML’s BS < 0.60) is omitted. Stratigraphic chart according to the International Commission on Stratigraphy, 2015. An expanded variant of the chronogram is presented in Supplementary Fig. 2. The putative distribution of the clades is based on the available DNA sequences. The out-group taxa are not shown.

Searching with MrBayes, BEAST and RAxML returned a similar well-resolved topology (Fig. 2 and Supplementary Figs 1 and 2). These phylogenetic models support three subfamilies, Pseudodontinae, Rectidentinae and Parreysiinae, whose representatives are widely distributed across the Oriental Region. In addition to previously described tribes, we found two new distant clades: Leoparreysiini Vikhrev, Bolotov et Kondakov **tribe nov**. and Pilsbryoconchini Bolotov, Vikhrev et Tumpeesuwan **tribe nov**. Our results support the majority of previously designated genera of the Indo-Chinese Unionidae, but the new phylogeny reveals that three genera should be restored and that two genera are new to science (Fig. 2).

**Figure 3:**
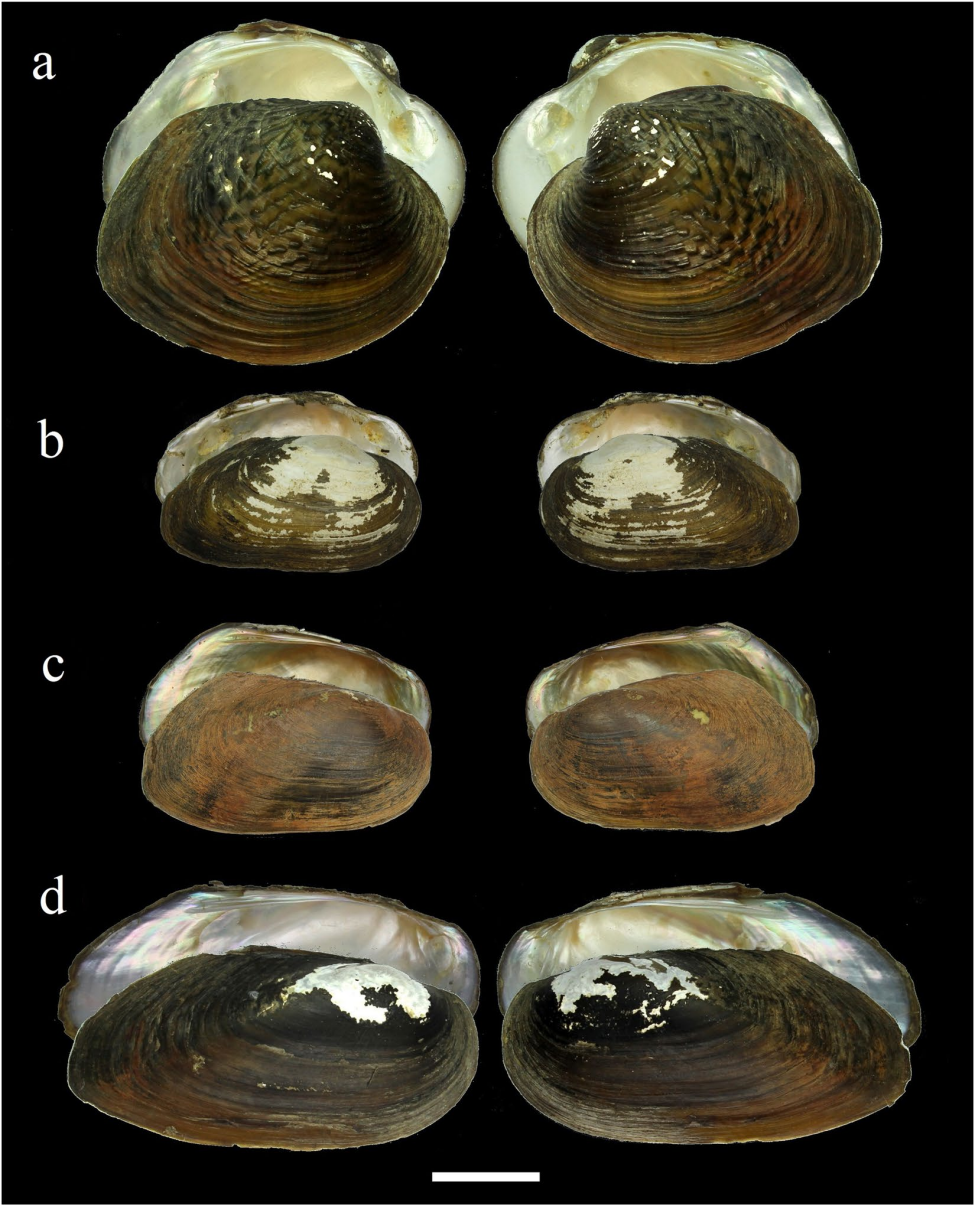
Shells of the endemic Parreysiinae taxa from the Sittaung River basin. **(a)** *Leoparreysia canefrii* Vikhrev, Bolotov et Kondakov **gen**. et **sp**. **nov**., Sittaung River near Taungoo, Myanmar (holotype RMBH biv254_4). **(b)** *Radiatula mouhoti* Vikhrev, Bolotov et Konopleva **sp**. **nov**., Sittaung River near Taungoo, Myanmar (holotype RMBH biv256). **(c)** *Lamellidens brandti* Bolotov, Konopleva et Vikhrev **sp**. **nov**., Pathi River, Myanmar (holotype RMBH biv243_14). **(d)** *Trapezidens obesa feae* Kondakov, Konopleva et Vikhrev **gen**. et **ssp**. **nov**., Myit Kyi Pauk Stream, Myanmar (holotype RMBH biv250_4). Scale bar = 2 cm. (Photos: Ekaterina S. Konopleva).

**Figure 4:**
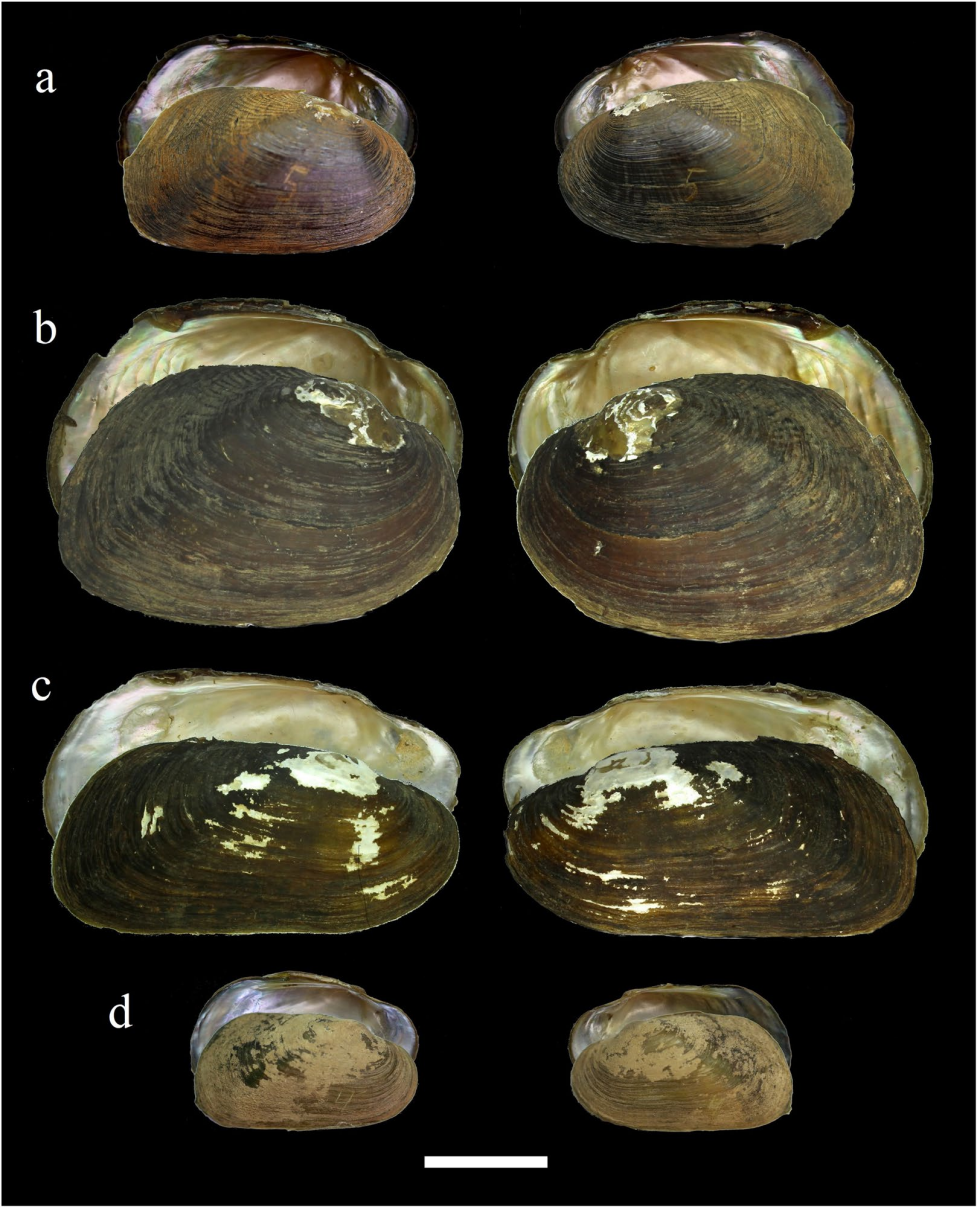
Shells of the endemic Pseudodontinae and Rectidentinae taxa from the Sittaung River basin. Pseudodontinae **(a**,**b)**, including **(a)** *Pseudodon bogani* Bolotov, Kondakov et Konopleva **sp**. **nov**., Kanni River, Myanmar (holotype RMBH biv241_5), and **(b)** *P*. *manueli* Konopleva, Kondakov et Vikhrev **sp**. **nov**., Pyowne Stream, Myanmar (holotype RMBH biv246_3). Rectidentinae **(c**,**d)**, including **(c)** *Trapezoideus nesemanni* Konopleva, Vikhrev et Bolotov **sp**. **nov**., Tauk Ue Kupt River, Myanmar (holotype RMBH biv255_2), and **(d)** *T*. *panhai* Konopleva, Bolotov et Kondakov **sp**. **nov**., Kyan Hone River (holotype RMBH biv138_4). Scale bar = 2 cm. (Photos: Ekaterina S. Konopleva).

Eight species and a single subspecies from the Sittaung were considered new to science, because they represent distinct endemic lineages and reveal significant morphological and molecular differences from previously known taxa (Fig. 1, Tables 1 and 2). The majority of these taxa are sisters to species from the Irrawaddy, indicating multiple ancient connections between these basins since the Oligocene (Fig. 1 and Supplementary Fig. 2). In this study, we describe seven species and a subspecies (Figs 3 and 4) because our sample of *Oxynaia* sp. “Taungoo” is too small and needs to be expanded in the future. Additionally, *Lamellidens savadiensis* **stat**. **res**. inhabits both the Sittaung and Irrawaddy drainages (Table 3). However, the presence of a single haplotype in the Sittaung instead of multiple haplotypes in the Irrawaddy (Fig. 1) may indicate a recent natural or human-mediated invasion of this species into the Sittaung. The habitats of each taxon that we collected are listed in Supplementary Table 3. All of the type series are deposited in RMBH, Russian Museum of Biodiversity Hotspots, the Federal Center for Integrated Arctic Research, Russian Academy of Sciences (Arkhangelsk, Russia).

**Table 1:** Shell measurements and reference DNA sequences for the type series of new taxa from the Sittaung River basin.

Taxon	Status of Specimen	Specimen Voucher*	Shell Length, mm	Shell Height, mm	Shell Width, mm	NCBI’s GenBank acc. nos.		
						COI	16S rRNA	28S rDNA
*Leoparreysia canefrii* Vikhrev, Bolotov et Kondakov **gen**. et **sp**. **nov**.	Holotype	biv254_4	67.7	50.7	30.8	MF352251	n/a	n/a
	Paratype	biv254_6	65.0	51.8	31.8	MF352252	n/a	n/a
	Paratype	biv249	20.0	14.6	9.2	MF352237	MF352307	MF352365
	Paratype	biv252_1	69.1	51.7	32.6	MF352245	MF352315	MF352373
	Paratype	biv252_3	28.3	21.3	12.6	MF352247	n/a	n/a
	Paratype	biv252_2	34.4	27.1	16.5	MF352246	MF352316	MF352374
	Paratype	biv254_2	76.0	56.4	33.6	MF352250	MF352319	MF352377
*Radiatula mouhoti* Vikhrev, Bolotov et Konopleva **sp**. **nov**.	Holotype	biv256	48.1	25.1	19.7	MF352257	MF352321	MF352382
	Paratype	biv253_1	48.7	28.1	18.6	MF352248	MF352317	MF352375
	Paratype	biv248_1	46.6	25.5	19.2	MF352234	MF352305	MF352363
	Paratype	biv248_3	32.2	28.7	11.6	MF352235	n/a	n/a
	Paratype	biv248_4	39.4	24.0	14.6	MF352236	MF352306	MF352364
	Paratype	biv253_6	45.7	26.7	17.3	MF352249	MF352318	MF352376
*Lamellidens brandti* Bolotov, Konopleva et Vikhrev **sp**. **nov**.	Holotype	biv243_14	53.6	30.0	17.1	MF352224	n/a	n/a
	Paratype	biv243_17	57.2	30.7	17.0	n/a	n/a	n/a
	Paratype	biv247_10	62.6	35.5	17.9	MF352232	MF352304	MF352362
	Paratype	biv242_3	57.3	29.0	19.5	MF352219	MF352293	MF352351
	Paratype	biv250_13	56.6	32.5	18.2	MF352241	MF352311	MF352369
	Paratype	biv243_10	52.3	29.2	16.6	MF352222	MF352296	MF352354
	Paratype	biv244_5	53.8	32.0	18.6	MF352227	MF352299	MF352357
	Paratype	biv244_2	57.5	34.0	21.1	MF352225	n/a	n/a
	Paratype	biv244_3	48.6	28.5	15.2	MF352226	MF352298	MF352356
*Trapezidens obesa feae* Kondakov, Konopleva et Vikhrev **gen**. et **ssp**. **nov**.	Holotype	biv250_4	75.7	37.1	22.1	MF352238	MF352308	MF352366
	Paratype	biv250_7	70.2	35.1	19.6	MF352239	MF352309	MF352367
	Paratype	biv250_8	70.4	35.1	20.2	MF352240	MF352310	MF352368
	Paratype	biv255_1	95.2	45.6	28.6	MF352253	MF352320	MF352378
	Paratype	biv250_3	79.8	38.5	23.3	n/a	n/a	n/a
*Pseudodon bogani* Bolotov, Kondakov et Konopleva **sp**. **nov**.	Holotype	biv241_5	57.5	32.7	17.4	MF352217	MF352291	MF352349
	Paratype	biv241_8	56.3	32.3	17.0	MF352218	MF352292	MF352350
	Paratype	biv241_4	62.6	40.0	21.8	MF352216	MF352290	MF352348
	Paratype	biv241_7	58.8	35.2	18.4	n/a	n/a	n/a
	Paratype	biv241_6	56.0	33.3	17.0	n/a	n/a	n/a
*Pseudodon manueli* Konopleva, Kondakov et Vikhrev **sp**. **nov**.	Holotype	biv246_3	79.6	51.8	26.1	MF352229	MF352301	MF352359
	Paratype	biv246_1	76.0	50.0	28.3	MF352228	MF352300	MF352358
	Paratype	biv246_8	65.8	41.5	23.1	MF352230	MF352302	MF352360
	Paratype	biv246_6	69.6	44.0	23.2	n/a	n/a	n/a
	Paratype	biv246_2	82.1	52.3	27.4	n/a	n/a	n/a
*Trapezoideus nesemanni* Konopleva, Vikhrev et Bolotov **sp**. **nov**.	Holotype	biv255_2	82.5	39.4	22.7	MF352254	n/a	MF352379
	Paratype	biv255_3	81.8	39.0	20.3	MF352255	n/a	MF352380
	Paratype	biv144_14	44.2	24.2	12.9	KX865906	KX865663	KX865777
	Paratype	biv144_25	28.6	16.4	7.8	KX865907	KX865664	KX865778
	Paratype	biv144_19	43.3	23.8	12.5	KX865908	KX865665	KX865779
*Trapezoideus panhai* Konopleva, Bolotov et Kondakov **sp**. **nov**.	Holotype	biv138_4	36.8	19.5	11.7	KX865909	KX865666	KX865780
	Paratype	biv138_7	37.0	20.8	11.9	KX865910	KX865667	KX865781
	Paratype	biv155_4	47.2	26.1	17.9	KX865911	KX865668	KX865782
	Paratype	biv155_25	35.8	18.4	11.4	KX865912	KX865669	KX865783
	Paratype	biv138_12	31.7	18.2	10.9	KX865913	KX865670	KX865784
	Paratype	biv155_11	46.4	25.5	16.2	KX865914	KX865671	KX865785

*All of the type series are deposited in RMBH, Russian Museum of Biodiversity Hotspots, the Federal Center for Integrated Arctic Research, Russian Academy of Sciences (Arkhangelsk, Russia). n/a – not available.

**Table 2:** Molecular diagnoses of the new taxa from the Sittaung River basin.

Taxon	Mean COI p-distance from the nearest neighbor of a new taxon, %	The nearest neighbor of a new taxon	Support of MOTUs by different versions of the PTP models			Fixed nucleotide differences based on the sequence alignment of congeners		
			bPTP	sPTP	mPTP	COI	16S rRNA	28S rDNA
*Leoparreysia canefrii* Vikhrev, Bolotov et Kondakov **gen**. et **sp**. **nov**.	6.2	*L*. *olivacea* **comb**. **nov**. (Irrawaddy)	Yes	Yes	Yes	11 C, 13 T, 199 G, 235 C, 242 C, 286 G, 349 G, 469 G, 514 T	13 C, 46 A, 49 C, 228 A, 248 T, 253 C, 294 T, 296 A, 318 T	224 G, 482 T, 584 G, 591 G, 643 G, 722 T
*Radiatula mouhoti* Vikhrev, Bolotov et Konopleva **sp**. **nov**.	4.4	*R*. aff. *bonneaudii* sp.1 (Irrawaddy)	Yes	Yes	No	28 A, 34 T, 94 G, 205 T, 250 C, 319 A, 334 C, 625 A	144 T, 262 C	n/a
*Lamellidens brandti* Bolotov, Konopleva et Vikhrev **sp**. **nov**.	2.7	*L*. aff. *marginalis* sp.3 (Kaladan)	Yes	No	No	193 C, 478 C	13 G, 18 C, 19 T, 155 C/A, 246 C/T	n/a
*Trapezidens obesa feae* Kondakov, Konopleva et Vikhrev **gen**. et **ssp**. **nov**.	2.4	*T*. *obesa obesa* **stat**. **res**. et **comb**. **nov**. (Irrawaddy)	Yes	Yes	No	7 A, 64 C, 127 A, 292 T, 313 T, 428 T, 574 T	19 T, 27 G, 49 G, 157 T, 158 T	584 T, 585 G, 586 C, 587 G
*Pseudodon bogani* Bolotov, Kondakov et Konopleva **sp**. **nov**.	7.9	*P*. *avae* (Irrawaddy)	Yes	Yes	Yes	37 A, 55 T, 70 T, 76 A, 92 C, 106 C, 163 G, 196 A, 205 C, 238 C, 250 C, 253 A, 307 G, 325 C, 349 A, 437 G, 457 C, 482 G, 502 A, 517 G, 538 A, 541 A, 559 A, 649 A, 652 C	48 T, 238 C, 242 G, 249 T, 322 C	482 C
*Pseudodon manueli* Konopleva, Kondakov et Vikhrev **sp**. **nov**.	9.9	*P*. *bogani* **sp**. **nov**. (Sittaung)	Yes	Yes	Yes	7 A, 10 C, 22 A, 25 G, 44 C, 70 G, 91 G, 124 C, 133 C, 157 C, 175 G, 205 G, 220 C, 226 C, 229 A, 238 G, 262 A, 274 G, 283 C, 292 G, 295 G, 316 A, 343 A, 355 A, 370 T, 382 C, 403 A, 448 C, 466 G, 472 A, 487 C, 496 T, 529 T, 535 G, 544 G, 592 C, 595 C, 598 G, 607 A, 613 C, 622 C	19 T, 140 G, 244 C, 256 T, 263 G, 293 A, 307 C, 308 C, 334 G	n/a
*Trapezoideus nesemanni* Konopleva, Vikhrev et Bolotov **sp**. **nov**.	2.6	*T*. *subclathratus* **stat**. **res**. et **comb**. **nov**. (Irrawaddy)	Yes	Yes	Yes	10 C, 157 T, 376 C, 484 C, 598 G, 616 C, 637 A, 655 A	192 C, 256 T, 298 G	116 T, 165 G*
*Trapezoideus panhai* Konopleva, Bolotov et Kondakov **sp**. **nov**.	5.8	*T*. *subclathratus* **stat**. **res**. et **comb**. **nov**. (Irrawaddy)	Yes	Yes	Yes	61 A, 148 A, 178 C, 211 A, 226 C, 253 A, 287 C, 295 T, 301 C, 352 G, 370 A, 403 G	315 G, 316 T, 329 A, 339 C	116 T, 165 G*

*Both species from the Sittaung reveal these diagnostic differences. The species delimitation models are as follows: bPTP of Zhang *et al*.^78^, sPTP of Kapli *et al*. (p < 0.001)^79^, and mPTP of Kapli *et al*.^79^ n/a – not available.

**Table 3:** List of the native Unionidae species from western Indo-China with new synonymies.

Genus	Species	Type Locality	Distribution
*Leoparreysia* Vikhrev, Bolotov et Aksenova **gen**. **nov**.	*L*. *canefrii* Vikhrev, Bolotov et Kondakov **sp**. **nov**.	Sittaung River near Taungoo	Sittaung
	*L*. *olivacea* (Prashad, 1930) **comb**. **nov**.	Kamaing in the Myitkyina District, Upper Burma^44^	Irrawaddy
	**L. *tavoyensis* (Gould, 1843) **comb**. **nov**.	Tavoy, British Burmah^26^	Tavoy
	**L. *bhamoensis* (Theobald, 1873) **comb**. **nov**. [**=** *Unio mandelayensis* Theobald, 1873 **syn**. **nov**.]	Bhamo, Burmah^32^	Irrawaddy
	*L. *burmana* (Blanford, 1869) **comb**. **nov**.	Ava and Bhamo^33^	Irrawaddy
	*L. *choprae* (Prashad, 1930) **comb**. **nov**.	Indawgyi Lake and a hill stream about five miles from Hopin towards Namna, Myitkyina^44^	Irrawaddy
	*L. *perconvexa* (Preston, 1912) **comb**. **nov**.	Nangyong Lake, Burma^41^	Irrawaddy
	*L. *vulcanus* (Hanley, 1875) **stat**. **res**. et **comb**. **nov**.	Birmah, vel Pegu^34^	Pegu
	*L. *feae* (Tapparone Canefri, 1889) **comb**. **nov**.	Meetan, Houngdaran River, Burma^38^	Salween
	*L. *houngdarauica* (Tapparone Canefri, 1889) **comb**. **nov**.	Meetan, Houngdaran River, Burma^38^	Salween
	?*L. *feddeni* (Theobald, 1873) **comb**. **nov**.	?Upper Burmah^31, 36, 43^	?Irrawaddy
*Radiatula* Simpson, 1900	*R*. *mouhoti* Vikhrev, Bolotov et Konopleva **sp**. **nov**.	Sittaung River near Taungoo	Sittaung
	*R*. *myitkyinae* (Prashad, 1930) **stat**. **res**. et **comb**. **nov**.	Kamaing in the Myitkyina District^44^	Irrawaddy
	*R*. aff. *bonneaudii* (Eydoux, 1838) sp.1	n/a	Irrawaddy
	*R. *chaudhurii* (Preston, 1912)	Upper Burma^41^	?Irrawaddy
	*R. *crispisulcata* (Benson, 1862)	Rivulo Bangong, prope Thyet-Myo, regionis Burmanicae^46^	Irrawaddy
*Oxynaia* Haas, 1913	*O*. sp. ‘Taungoo’	n/a	Sittaung
	*O*. sp. ‘Tavoy’	n/a	Tavoy
	*O*. *pugio* (Benson, 1862)	Regione Ava^46^	Irrawaddy
*Indonaia* Prashad, 1918 **stat**. **res**.	*I*. *andersoniana* (Nevill, 1877) **comb**. **res**.	Myadoung, Burma^36^	Irrawaddy
*Lamellidens* Simpson, 1900	*L*. *brandti* Bolotov, Konopleva et Vikhrev **sp**. **nov**.	Pathi River	Sittaung
	*L*. *savadiensis* (Nevill, 1877) **stat**. **res**. [=*Unio gianellii* Tapparone Canefri, 1889 **syn**. **nov**.; =*Lamellidens canefrianus* Simpson, 1900 **syn**. **nov**.; =*L*. *nongyangensis* Preston, 1912 **syn**. **nov**.; =*L*. *indawgyiensis* Prashad, 1930 **syn**. **nov**.]	At Sawady in the Thengleng stream, also at Bhamo and at Shuaygoomyo^36^	Irrawaddy and Sittaung
	*L*. *generosus* (Gould, 1847) [=*Physunio ferrugineus* Annandale, 1918 **syn**. **nov**.; =*P*. *micropteroides* Annandale, 1918 **syn**. **nov**.; =*Lamellidens burmanus* Simpson, 1914]	Newville, Tavoy, British Burmah^28^	Salween
	*L*. aff. *marginalis* (Lamarck, 1819) sp.3	n/a	Kaladan
*Trapezidens* Bolotov, Vikhrev et Konopleva **gen**. **nov**.	*T*. *obesa* (Hanley et Theobald, 1876) **stat**. **res**. et **comb**. **nov**. with the following subspecies: *T*. *o*. *obesa* (Hanley et Theobald, 1876) [=*Unio dolichorhynchus* Tapparone Canefri, 1889 **syn**. **nov**.; =*U*. *scutum* var. *humilior* Martens, 1899 **syn**. **nov**.] from the Irrawaddy, *T. *o*. *angustior* (Hanley et Theobald, 1876) **stat**. **res**. et **comb**. **nov**. from the Pegu, and *T*. *o*. *feae* Kondakov, Konopleva et Vikhrev **ssp**. **nov**. from the Sittaung	River Iriwadi, Birmah^35^	Irrawaddy, Pegu and Sittaung
	*T*. *exolescens* (Gould, 1843) **comb**. **nov**.	Tavoy, Burmah^25, 26^	Tavoy
	*T. *scutum* (Sowerby, 1868) **comb**. **nov**.	Tenasserim^30, 83^	Tenasserim
*Pseudodon* Gould, 1844	*P*. *bogani* Bolotov, Kondakov et Konopleva **sp**. **nov**.	Kanni River	Sittaung
	*P*. *manueli* Konopleva, Kondakov et Vikhrev **sp**. **nov**.	Pyowne Stream	Sittaung
	*P*. *avae* (Theobald, 1873)	Mandalay, Burmah^32^	Irrawaddy
	*P. *peguensis* (Anthony, 1865)	Pegu, British Burmah^47^	Pegu
	*P. *crebristriata* (Anthony, 1865)	Pegu, British Burmah^47^	Pegu
	*P. *inoscularis* (Gould, 1844)	River Salwen, Tavoy, Brit. Burmah^27^	Salween
	*P. *salweniana* (Gould, 1844)	Salwen River, British Burmah^27^	Salween
*Trapezoideus* Simpson 1900	*T*. *nesemanni* Konopleva, Vikhrev et Bolotov **sp**. **nov**.	Tauk Ue Kupt River	Sittaung
	*T*. *panhai* Konopleva, Bolotov et Kondakov **sp**. **nov**.	Kyan Hone River	Sittaung
	*T*. *subclathratus* (Martens, 1899) **stat**. **res**. et **comb**. **nov**. [=*T*. *zayleymanensis* Preston, 1912 **syn**. **nov**.]	Chindwinfluss bei Kalewa and bei Matu^37^	Irrawaddy
	*T*. sp. ‘Salween’	n/a	Salween
	*T. *foliaceus* (Gould, 1843)	Tavoy, Burmah	Tavoy
	*T. *peguensis* (Anthony, 1865)	Pegu, British Burmah^84^	Pegu

*Nominal taxa whose DNA sequences are not available. All of the other taxa were studied by means of a molecular approach (see Table 2 and Supplementary Table 1 for reference sequences). **The phylogenetic relationships between *Leoparreysia tavoyensis* **comb**. **nov**. and *L*. *bhamoensis* **comb**. **nov**. are not well resolved, and we previously suggested that *L*. *tavoyensis* inhabits both the Tavoy and Irrawaddy catchments^9^. However, an expanded sampling reveals that they are relatively distant phylogenetic lineages based on the 28S rDNA gene despite their high similarity in the mtDNA. n/a – not available. The coordinates of the type localities of the new taxa described from the Sittaung are presented in Supplementary Table 2.

**Taxonomic account**

**Family Unionidae Rafinesque**, **1820**

**Subfamily Parreysiinae Henderson**, **1935**

Type Genus: *Parreysia* Conrad, 1853 (by original designation)

**Comments:** This subfamily includes at least five valid tribes: Parreysiini Henderson, 1935, Coelaturini Modell, 1942, Lamellidentini Modell, 1942, Oxynaiini Starobogatov, 1970^17^ and Leoparreysiini Vikhrev, Bolotov et Kondakov, a new tribe described here.

**Distribution:** Africa, South and Southeast Asia^9, 17, 24, 45, 51–54^.

**Tribe Leoparreysiini Vikhrev**, **Bolotov et Kondakov tribe nov**.

Type Genus: *Leoparreysia* Vikhrev, Bolotov et Aksenova **gen**. **nov**.

**Comments:** This tribe includes only the genus *Leoparreysia* Vikhrev, Bolotov et Aksenova **gen**. **nov**.

**Diagnosis:** Shell very thick, shape of round, umbo rather prominent and situated near the anterior end. Pseudocardinal teeth well developed; laterals convex and thick. Anterior adductor scar round and very deep.

**Distribution:** Western Indo-China.

Genus *Leoparreysia* Vikhrev, Bolotov et Aksenova **gen**. **nov**.

Type Species: *Leoparreysia canefrii* Vikhrev, Bolotov et Kondakov **sp**. **nov**.

**Comments:** We assigned eleven species to the genus, including a new species from the Sittaung River basin (Table 3).

**Etymology:** This genus is named after Leonardo Fea (1852–1903), an adventurous Italian naturalist, who sampled freshwater mussels from unexplored areas of British Burma (Myanmar).

**Diagnosis:** The same as for the tribe.

**Distribution:** Western Indo-China.

*Leoparreysia canefrii* Vikhrev, Bolotov et Kondakov **sp**. **nov**.

**Figure 5:**
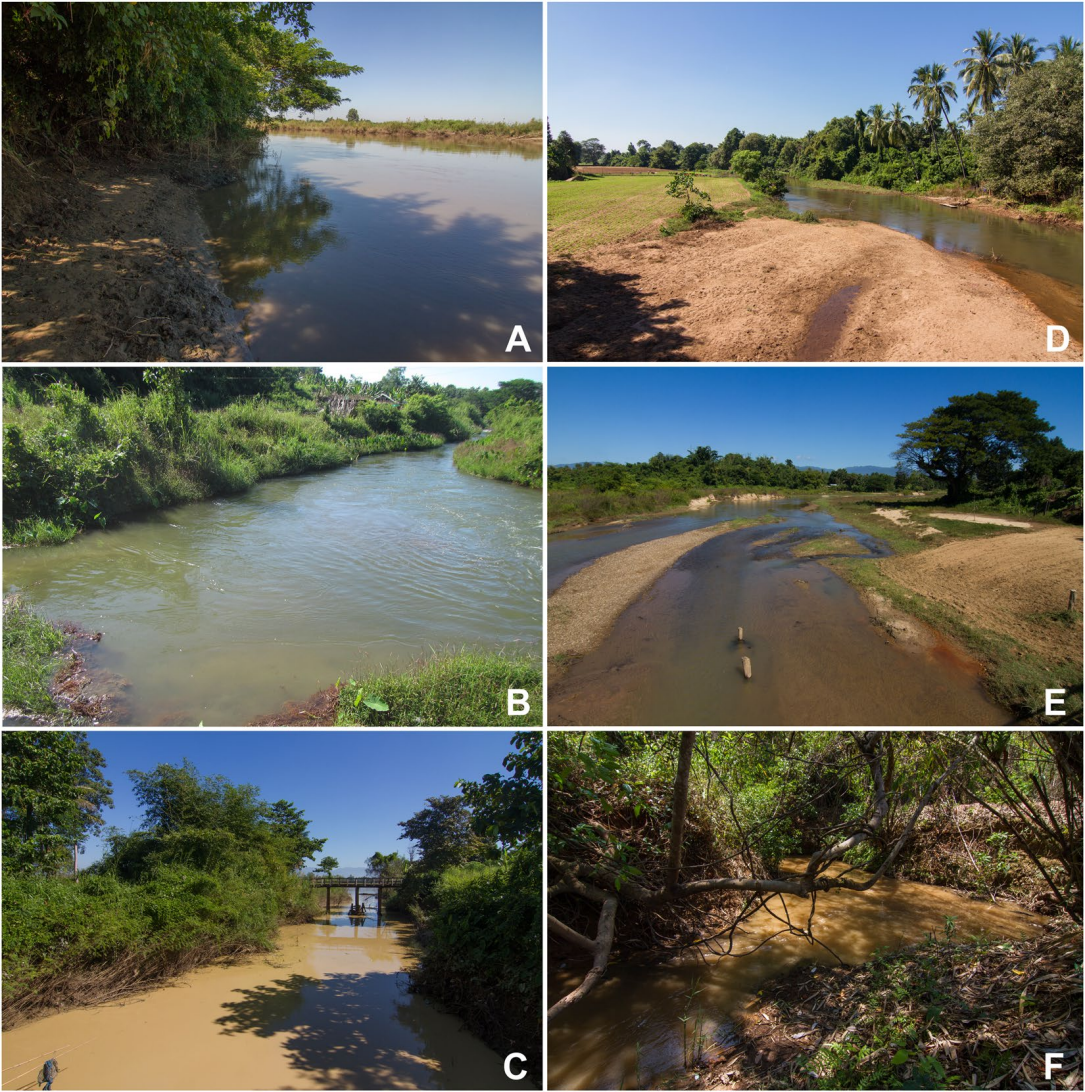
The type localities of new taxa from the Sittaung Basin. (**a**) Sittaung River near Taungoo (*Leoparreysia canefrii* **gen**. et **sp**. **nov**. and *Radiatula mouhoti* **sp**. **nov**.). (**b**) Pathi River (*Lamellidens brandti* **sp**. **nov**.). (**c**) Myit Kyi Pauk Stream (*Trapezidens obesa feae* **gen**. et **ssp**. **nov**.). (**d**) Kanni River (*Pseudodon bogani* **sp**. **nov**.). (**e**) Pyowne Stream (*Pseudodon manueli* **sp**. **nov**.). (**f**) Kyan Hone River (*Trapezoideus panhai* **sp**. **nov**.). (Photos: Ilya V. Vikhrev).

Figures 3a and 5a, Tables 1 and 2

**Type material:** Holotype RMBH biv254_4: Myanmar: Sittaung near Taungoo, 26.xi.2016, Vikhrev leg. Paratypes: the type locality, 6 specimens (RMBH biv254_6, biv254_2, biv249, biv252_1, biv252_3, and biv252_2), 25–26.xi.2016, Vikhrev leg.

**Etymology:** This species is named after Cesare Maria Tapparone-Canefri (1838–1891), an Italian malacologist, who described numerous mussel taxa from British Burma (Myanmar).

**Diagnosis:** The new species is similar to *L*. *olivacea* **comb**. **nov**. and *L*. *tavoyensis* **comb**. **nov**. but differs in a more inequilateral shell shape, specific shell sculpture with zigzag ridges, deeper umbo cavity, and fixed nucleotide substitutions (Table 2).

**Description:** Shell shape oval to slightly elliptic, not very inequilateral, inflated, heavy and thick with zigzag ridges ranging from umbo, obscured in several adults. Posterior ridge somewhat oblique. Shell length (SL) 20.0–76.0 mm, height (SH) 14.6–56.4 mm, width (SW) 9.2–33.6 mm. Shell sculpture strong. Periostracum dark-olive to yellowish-brown; nacre white, sometimes with yellow spots. Umbo pronounced; beak sculpture strong. Left valve with two short curved lateral teeth and two striate pseudocardinals. Right valve with a single curved lateral tooth and two distinct pseudocardinals; anterior tooth strongly indented, massive, posterior tooth small and lamellar. Umbo cavity deep. Anterior adductor scar well-marked, very deep, funneled; posterior scar shallow.

**Distribution:** Sittaung River.

**Tribe Oxynaiini Starobogatov**, **197**0

Type Genus: *Oxynaia* Haas, 1913 (designated in this study)

**Comments:** This tribe includes at least three phylogenetically distant genus-level clades: *Radiatula* Simpson, 1900 (see below), *Indonaia* Prashad, 1918 **stat**. **res**. (type species: *Unio caeruleus* Lea, 1831, by original designation; type locality: Bengal, India) and *Oxynaia* Haas, 1913 (type species: *Unio jourdyi* Morlet, 1886, by original designation; type locality: Tonkin, environs de Dang-son). The latter clade may belong to another, possibly undescribed genus, but the *Oxynaia jourdyi* sequences are not available. A single *Indonaia* species and three *Oxynaia* taxa were found in western Indo-China (Table 3).

**Distribution:** Southeast and South Asia^9, 17, 45^.

Genus *Radiatula* Simpson, 1900

Type Species: *Unio crispisulcatus* Benson, 1862 (by original designation; type locality: rivulo Bangong, prope Thyet-Myo, regionis Burmanicae)

**Comments:** Five species of the genus are recorded in western Indo-China, including a new species from Sittaung (Table 3).

**Distribution:** Southeast Asia^9, 39, 40, 45^.

*Radiatula mouhoti* Vikhrev, Bolotov et Konopleva **sp**. **nov**.

Figures 3b and 5a, Tables 1 and 2

**Type material:** Holotype RMBH biv256: Myanmar: Sittaung near Taungoo, 26.xi.2016, Vikhrev leg. Paratypes: Myanmar: the type locality, 5 specimens (RMBH biv253_1, biv253_6, biv248_4, biv248_1, and biv248_3), 25–26.xi.2016, Vikhrev leg.

**Etymology:** This species is named after Henri Mouhot (1826–1861), a French naturalist and explorer, who travelled across Siam, Cambodia and Laos.

**Diagnosis:** The new species is similar to *R*. aff. *bonneaudii* sp.1, but differs by a less deep and rounded anterior adductor scar, a well-marked posterior scar, and fixed nucleotide substitutions (Table 2).

**Description:** Shell elliptical, elongate, inequilateral, somewhat inflated, ventral margin straight; with thin wrinkles from umbo along dorsal margin; sculptured in the umbo area. Posterior ridge oblique, narrow. SL 32.2–48.7 mm, SH 24.0–28.7 mm, SW 11.6–19.7 mm. Periostracum smooth, olive, slightly greenish, some shells with dark-brown ventral margin, nacre yellowish with visible pallial line. Umbo not prominent, beak sculpture not well developed. Left valve with two short lateral teeth and two ribbed pseudocardinals. Right valve with a single lateral tooth and two distant pseudocardinals; anterior tooth strong, indented; posterior tooth slender. Umbo cavity rather deep. Anterior adductor scar pronounced, oval; posterior scar well-marked, rounded.

**Distribution:** Sittaung River.

**Tribe Lamellidentini Modell**, **1942**

Type Genus: *Lamellidens* Simpson, 1900 (by original designation)

**Comments:** This tribe includes at least two distant genus-level clades: *Lamellidens* Simpson, 1900 and *Trapezidens* Bolotov, Vikhrev et Konopleva **gen**. **nov**.

**Distribution:** South and Southeast Asia^9, 17, 45^.

Genus *Lamellidens* Simpson, 1900

Type Species: *Unio marginalis* Lamarck, 1819 (by original designation)

**Comments:** A large genus, which includes numerous nominal taxa^24, 40, 43–45, 51, 52^ and multiple cryptic DNA lineages, particularly from India^9^. In contrast, the species diversity of *Lamellidens* in western Indo-China was largely overestimated, as we were able to find only four species there (Table 3). Based on the DNA sequences and conchological features of the topotypes, *Physunio ferrugineus* **syn**. **nov**. and *P*. *micropteroides* **syn**. **nov**., two nominal taxa from Lake Inle, are synonyms of *Lamellidens generosus*, which is the only member of the genus in the Salween River basin (Supplementary Fig. 3 and Table 3). It is another example of incorrect placement of Lamellidentini taxa within the Contradentini because of the convergent similarity in the shell shape^25^. In the Irrawaddy, we recorded only *Lamellidens savadiensis* **stat**. **res**. (Table 3). This species was also found in the Sittaung, where it lives together with a species new to science, which is described here.

**Distribution:** South Asia and western Indo-China^9, 45^.

*Lamellidens brandti* Bolotov, Konopleva et Vikhrev **sp**. **nov**.

Figures 3c and 5b, Tables 1 and 2

**Type material:** Holotype RMBH biv243_14: Myanmar: Sittaung, Pathi River, 23.xi.2016, Vikhrev leg. Paratypes: the type locality, 3 specimens (RMBH biv242_3, biv243_17 and biv243_10), Myanmar: Sittaung, reservoir at Yetho River, 3 specimens (RMBH biv244_5, biv244_2 and biv244_3), Myanmar: Sittaung, fishing ponds near Taungoo, 1 specimen (RMBH biv247_10), Myanmar: Sittaung, Myit Kyi Pauk Stream, 1 specimen (RMBH no. biv250_13), 23–26.xi.2016, Vikhrev leg.

**Etymology:** This species is named after Dr. Rolf Arthur Max Brandt (1917–1989), a famous German malacologist, who studied the mussels of Southeast Asia.

**Diagnosis:** The new species is similar to *L*. *savadiensis*, but differs by a smaller shell, shallow anterior and posterior adductor scars, and fixed nucleotide substitutions (Table 2).

**Description:** Shell oval or trapezoidal, elongated, with more or less pronounced postdorsal wing, thin, inequilateral, not inflated. Posterior ridge broad, angular. SL 48.6–62.6 mm, SH 28.5–35.5 mm, SW 15.2–21.1 mm. Shell sculpture not strong. Periostracum smooth, brown, with yellowish concentric bands, mainly along the ventral margin; nacre white-bluish, shining. Umbo not prominent, beak sculpture slightly prominent. Left valve with two parallel lateral teeth and one lamellar pseudocardinal. Right valve with a single straight lateral tooth and two thin lamellar pseudocardinals. Umbo cavity not deep. Muscle adductor scars are shallow.

**Distribution:** Sittaung River Basin.

Genus *Trapezidens* Bolotov, Vikhrev et Konopleva **gen**. **nov**.

Type Species: *Unio exolescens* Gould, 1843 (type locality: Tavoy, Burmah)

**Comments:** A small remarkable genus with three species: *T*. *exolescens* **comb**. **nov**., *T*. *obesa* **stat**. **res**. et **comb**. **nov**. (with three subspecies) and *T*. *scutum* **comb**. **nov**. (Table 3).

**Diagnosis:** Shell slightly thickened, of trapezoidal or elliptical shape, umbo indistinct and situated near the anterior end. Pseudocardinal teeth well developed, triangular; laterals strait and thick. Anterior adductor scar round and rather deep.

**Etymology:** This genus is named after its type species, *Unio exolescens*, which was erroneously considered a member of another genus, *Trapezoideus* Simpson, 1900, due to the convergent similarity in the shell shape^25^.

**Distribution:** Western Indo-China^9^.

*Trapezidens obesa feae* Kondakov, Konopleva et Vikhrev **ssp**. **nov**.

Figures 3d and 5c, Tables 1 and 2

**Type material:** Holotype RMBH biv250_4: Myanmar: Sittaung, Myit Kyi Pauk Stream, 26.xi.2016, Vikhrev leg. Paratypes: the type locality, 3 specimens (RMBH biv250_7, biv250_8 and biv250_3), Myanmar: Sittaung near Taungoo, 1 specimen (RMBH biv255_1), 26.xi.2016, Vikhrev leg.

**Etymology:** This subspecies is named after Leonardo Fea (1852–1903), an Italian naturalist.

**Diagnosis:** The new subspecies differs by an almost straight dorsal margin without a wing, weakly developed adductor scars, a more oblong shell shape, and fixed nucleotide substitutions (Table 2).

**Description:** Shell elliptic, slightly inflated, very inequilateral, rather strong. Posterior ridge narrow, roundly angular. SL 70.2–95.2 mm, SH 35.1–45.6 mm, SW 19.6–28.6 mm. Periostracum blackish-brown with light-brown or sandy border along the ventral margin; nacre whitish. Left valve with two parallel straight lateral teeth and two pseudocardinals, anterior tooth well developed, posterior tooth small, undeveloped. Right valve with a single blade-shaped straight lateral tooth and two distant pseudocardinals, the lower tooth better developed, with small scratches. Anterior adductor scar not developed, of oval shape; posterior scar shallow.

**Distribution:** Sittaung River Basin.

**Subfamily Pseudodontinae Frierson**, **1927**

Type Genus: *Pseudodon* Gould, 1844 (by original designation)

**Comments:** This remarkable subfamily, which was restored by Bolotov *et al*.,^9^ includes the two valid tribes: Pseudodontini Frierson, 1927 and Pilsbryoconchini Bolotov, Vikhrev et Tumpeesuwan **tribe nov**. Frierson^55^ (p. 67) did not provide any description of the subfamily, but noted that “The above species [*Gonidea angulata* (Lea, 1838)] is usually without ‘teeth’, but sometimes bears large high teeth, one in each valve, strikingly alike to the *Pseudodon cambodgensis* [sic.], and the outward facies of the two species are quite similar, so that it might be that the species in fact a member of the Pseudodontinae.”

**Distribution:** Southeast and East Asia^9, 45^.

**Tribe Pseudodontini Frierson**, **1927**

Type Genus: *Pseudodon* Gould, 1844 (by original designation)

**Comments:** This tribe includes the single genus *Pseudodon* Gould, 1844. The genera *Trigonodon* Conrad, 1865 [type species: *Monocondyloea crebristriata* Anthony, 1865, by original designation; type locality: Pegu, British Burmah] and *Indopseudodon* Prashad, 1922 [type species: *Anodon salweniana* Gould, 1844; type locality: Salwen River, British Burmah] are considered synonyms of *Pseudodon* on the basis of conchological features.

**Distribution:** Western Indo-China.

Genus *Pseudodon* Gould, 1844

Type Species: *Anodon inoscularis* Gould, 1844 (by original designation; type locality: River Salwen, Tavoy, Brit. Burmah)

**Comments:** This genus contains seven species, including two new species from Sittaung (Table 3).

**Diagnosis:** Shell rather thick, of elliptical or round shape, umbo slightly prominent and situated near the anterior end. Pseudocardinal teeth tubercle-like, rather strong and prominent.

**Distribution:** Western Indo-China.

*Pseudodon bogani* Bolotov, Kondakov et Konopleva **sp**. **nov**.

Figures 4a and 5d, Tables 1 and 2

**Type material:** Holotype RMBH biv241_5: Myanmar: Sittaung, Kanni River, 23.xi.2016, Vikhrev leg. Paratypes: the type locality, 4 specimens (RMBH biv241_8, biv241_4, biv241_7 and biv241_6), 23.xi.2016, Vikhrev leg.

**Etymology:** This new species is dedicated to Dr. Arthur Bogan, a famous American malacologist, who developed the basic principles for the conservation of freshwater mussels worldwide.

**Diagnosis:** The new species is similar to *P*. *avae* and *P*. *manueli* **sp**. **nov**., but differs by a straighter ventral margin, massive hinge plate, and fixed nucleotide substitutions (Table 2).

**Description:** Shell ovate to elliptical, thick, very inequilateral, moderately inflated, umbo area ornamented by W-shaped wrinkles. Posterior ridge broader than the anterior ridge. SL 56.0–62.6 mm, SH 32.3–40.0 mm, SW 17.0–21.8 mm. Shell sculpture strong. Periostracum not smooth, with visible concentric bands, dark-brown; nacre peach-colored, yellowish. Umbo not projecting, corrugated, beak sculpture not prominent. A massive tubercle-like pseudocardinal tooth in each valve. Umbo cavity shallow. Anterior adductor scar well-marked, bean-like, rather deep; posterior scar rounded, visible.

**Distribution:** Kanni River.

*Pseudodon manueli* Konopleva, Kondakov et Vikhrev **sp**. **nov**.

Figures 4b and 5e, Tables 1 and 2

**Type material:** Holotype RMBH biv246_3: Myanmar: Sittaung, Pyowne Stream, 25.xi.2016, Vikhrev leg. Paratypes: the type locality, 4 specimens (RMBH biv246_1, biv246_8, biv246_6 and biv246_2), 25.xi.2016, Vikhrev leg.

**Etymology:** This species is dedicated to Dr. Manuel Lopes-Lima, a malacologist from Portugal, who developed a recent integrative system for the Unionidae.

**Diagnosis:** The new species is similar to *P*. *bogani* **sp**. **nov**. and *P*. *avae*, but differs by less pronounced pseudocardinals, adductor scars not produced, and fixed nucleotide substitutions (Table 2).

**Description:** Shell ovate to elliptical, not very thick, inequilateral, moderately inflated, dorsal margin ornamented by W-shaped wrinkles. Posterior ridge broader than the anterior ridge. SL 65.8–82.1 mm, SH 41.5–52.3 mm, SW 23.1–28.3 mm. Periostracum light brown; nacre white-yellowish. Umbo slightly projecting, somewhat corrugated, beak sculpture not strong. A small tubercle-like pseudocardinal in each valve. Umbo cavity shallow. Anterior adductor scar poorly visible; posterior scar shallow.

**Distribution:** Pyowne Stream.

**Tribe Pilsbryoconchini Bolotov**, **Vikhrev et Tumpeesuwan tribe nov**.

Type Genus: *Pilsbryoconcha* Simpson, 1900

**Comments:** This tribe includes at least three valid genera: *Pilsbryoconcha* Simpson, 1900 (type species: *Anodonta exilis* Lea, 1838, by original designation; type locality: unknown), *Monodontina* Conrad, 1853 **stat**. **res**. (type species: *Margaritana vondembuschiana* Lea, 1840, by original designation; type locality: Java), and *Bineurus* Simpson, 1900 **stat**. **res**. (type species: *Monocondyloea mouhotii* Lea, 1863, by original designation; type locality: Laos Mts., Cambodia, Siam). However, there are three distant, relatively well-supported clades, which may represent distinct genera (Fig. 2). An integrative revision of this large tribe should be undertaken in the future based on expanded sampling from the Mekong, as the phylogenetic relationships between these clades are still unresolved (Fig. 2).

**Diagnosis:** Shell rather thin, of elliptical or elongated shape, umbo slightly prominent and situated near the anterior end. Pseudocardinals reduced or lacking; laterals reduced.

**Distribution:** Paleo-Mekong basin^9^.

**Subfamily Rectidentinae Modell**, **1942**

**Comments:** This subfamily includes two tribes: Rectidentini Modell, 1942 (type genus: *Rectidens* Simpson, 1900) and Contradentini Modell, 1942 (see below).

**Distribution:** Southeast Asia, including the Greater Sunda Islands^9, 17, 45^.

**Tribe Contradentini Modell**, **1942**

Type Genus: *Contradens* Haas, 1911 (by original designation)

**Comments:** This tribe includes at least three genus-level phylogenetic clades: *Contradens* Haas, 1911, *Trapezoideus* Simpson 1900, and *Physunio* Simpson, 1900 (Fig. 2).

**Distribution:** Southeast Asia, including the Greater Sunda Islands^9, 17, 45^.

Genus *Trapezoideus* Simpson 1900

Type Species: *Unio foliaceus* Gould, 1843 (by original designation; type locality: Tavoy, Burmah)

**Comments:** The genus was established on the basis of conchological features^39, 40^. We consider this genus as a separate Contradentini clade, which includes taxa inhabiting the rivers of western Indo-China, although the molecular sequences of the type species are not available^25^. The genus contains at least six species, including two new taxa from the Sittaung (Table 3). Among the other taxa that were assigned to the genus, *Trapezoideus peninsularis* Simpson, 1900 [type locality: Sumatra] is surely a member of *Contradens*, but the position of Indian taxa such as *T*. *prashadi* Haas, 1922 [type locality: Mysore, Sudöstindien] and *T*. *theca* (Benson, 1862) [type locality: fluvio Cane, prope Banda, Bundelkhund] is still uncertain. The two latter taxa may belong to *Lamellidens* ^33, 46^, but their DNA sequences are not available.

**Diagnosis:** Shell rather thin, from a trapezoidal to elongate-elliptic form, the anterior margin narrower than the posterior margin. One lateral tooth and two pseudocardinals in the right valve, two laterals and elongate lamellar cardinal tooth in the left valve. Pseudocardinals reduced or lacking. Anterior adductor scar more or less produced, posterior scar usually less marked. Umbo more or less projected. Older specimens with more elongate shape and flattened umbo.

**Distribution:** Western Indo-China.

*Trapezoideus nesemanni* Konopleva, Vikhrev et Bolotov **sp**. **nov**.

Figure 4c, Tables 1 and 2

**Type material:** Holotype RMBH biv255_2: Myanmar: Sittaung, Tauk Ue Kupt River, 26.xi.2016, Vikhrev leg. Paratypes: the type locality, 4 specimens (RMBH biv255_3, biv_144_14, biv_144_25, and biv_144_19), 20.iv.2015 and 26.xi.2016, Bolotov, Vikhrev & locals leg.

**Etymology:** This new species is dedicated to Dr. Hasko Friedrich Nesemann, an Austrian malacologist, who made great contributions to the knowledge of molluscs from the Ganga River system.

**Diagnosis:** The new species is similar to *T*. *subclathratus* and *T*. *panhai* **sp**. **nov**., but differs in its very thin shell, shallow adductor scars, and fixed nucleotide substitutions (Table 2).

**Description:** Shell trapezoidal, thin, not inflated, anterior side rounded and narrow. Posterior ridge broad and sloped. SL 28.6–82.5 mm, SH 16.4–39.4 mm, SW 7.8–22.7 mm. Shell sculpture not strong. Periostracum brown to dark-brown; nacre yellowish. Umbo not prominent, with wrinkles in the umbo area, corrugated. Beak sculpture not strong, slightly pronounced. Right valve with two slightly beaked, flat pseudocardinals and one lateral tooth. Left valve with one elongate, flat pseudocardinal tooth and two laterals. Several specimens with a single thin lateral tooth in each valve and a single shallow, lamellar pseudocardinal in each valve. Umbo cavity shallow. Anterior adductor scar not deep, drop-like in shape. Posterior scar rounded, shallow.

**Distribution:** Tauk Ue Kupt River.

*Trapezoideus panhai* Konopleva, Bolotov et Kondakov **sp**. **nov**.

Figures 4d and 5f, Tables 1 and 2

**Type material:** Holotype RMBH biv_138_4: Myanmar: Sittaung, Kyan Hone River, 17.iv.2015, Bolotov leg. Paratypes: the type locality, 5 specimens (RMBH biv_138_7, biv_155_4, biv_155_25, biv_138_12, biv_155_11), 17–18.iv.2015, Bolotov & locals leg.

**Etymology:** This new species is dedicated to Prof. Dr. Somsak Panha, a famous Thai zoologist, who described numerous molluscan taxa from Southeast Asia.

**Diagnosis:** The new species is similar to *T*. *subclathratus* and *T*. *nesemanni* **sp**. **nov**., but differs by a thicker shell, more developed hinge and umbones, and fixed nucleotide substitutions (Table 2).

**Description:** Shell oval, elongated, with narrower anterior side and broader posterior end, rather thick, inequilateral. Ventral margin concave or straight. Posterior ridge broad. SL 31.7–47.2 mm, SH 18.2–26.1 mm, SW 10.9–17.9 mm. Shell sculpture rather strong. Periostracum brown to olive-brown; nacre whitish. Umbo somewhat prominent, beak sculpture not very strong, pronounced. Hinge plates with one lateral tooth and two pseudocardinals in the right valve, and two laterals and elongate lamellar cardinal in the left valve. Umbo cavity not very deep. Anterior adductor scar drop-like and deep; posterior scar somewhat oval-shaped and well-marked.

**Distribution:** Kyan Hone River.

## Discussion

### Taxonomic comments

The taxonomy of the Unionidae from western Indo-China is complicated^9, 10, 25^. Our study is the first attempt to establish an updated taxonomic scheme for the fauna of this region based on an integrative approach (Table 3). The results of this investigation indicate that the Irrawaddy represents the most species-rich basin (16 taxa, including 10 species sequenced), followed by the Sittaung (10 species, all sequenced), Salween (6 taxa, 2 species sequenced), and Tavoy (4 taxa, 3 species sequenced). The faunas of other basins are poorly known. Only five nominal taxa were described from the Pegu River, and none of them were sequenced. The small and medium sized basins of the western coast of Myanmar (Bay of Bengal and Andaman Sea) are almost unstudied.

There is a historical tradition to attribute numerous unionid species from western Indo-China to those described from Indian rivers, which began with the pioneering works^36–38, 46^ followed by the subsequent revisions^39, 40, 43–45, 50, 51^. This view was accepted until recently^10, 24^, but none of the true Indian taxa are actually distributed in western Indo-China^9^. For example, the records of *Lamellidens corrianus* (Lea, 1834), *L*. *jenkinsianus* (Benson, 1862), *L*. *lamellatus* (Lea, 1838), *L*. *marginalis* (Lamarck, 1819), *Parreysia corrugata* (Müller, 1774), *P*. *favidens* (Benson, 1862), *P*. *smaragdites* (Benson, 1862), *Radiatula bonneaudii* (Eydoux, 1838), *R*. *caerulea* (Lea, 1831), and *R*. *pachysoma* (Benson, 1862) in the Irrawaddy^10, 24, 44^ are erroneous. However, the occurrences mentioned above may correspond to morphologically similar but phylogenetically distinct, endemic taxa such as *Lamellidens savadiensis*, *Trapezidens obesa*, *Leoparreysia* spp., *Radiatula* aff. *bonneaudii* sp.1, and *Indonaia andersoniana*. The published records of *Lamellidens consobrinus* (Lea, 1860), *L*. *generosus* and *Physunio ferrugineus* from the Irrawaddy^10^ most likely refer to *Lamellidens savadiensis*.

### Biogeography and conservation

Our modeling confirms that the primary Indo-Chinese Unionidae clades are of Mesozoic origin and that the most ancient intra-area radiations occurred within the putative paleo-Mekong basin. These results agree with the model of Bolotov *et al*.^9^, although the present study suggests that the two largest paleo-Mekong radiations may have had a pre-Cenozoic origin (mean age = 65–71 Ma). Based on our fossil-calibrated phylogeny, the fauna of the Sittaung is related to that of the Irrawaddy but represents a separate evolutionary entity harboring a number of endemic lineages. Our model suggests that the majority of the lineages were separated during the Miocene (mean age = 8.0–22.1 Ma), but the split between *Pseudodon avae* and the two sister taxa from the Sittaung appears to be more ancient (mean age = 35.3 Ma) (Supplementary Fig. 2). These results are consistent with the model indicating that the major rivers of Indo-China represent ancient evolutionary hotspots with almost 100% level of endemism in the Unionidae^9^. In contrast, our modeling contradicts the hypothesis of an Early Miocene paleo-drainage joining the Salween and Mekong^56, 57^ because the Salween fauna relates to those of the Sittaung and Irrawaddy but not the Mekong. The most important biogeographic boundary between the unionid faunas in Indo-China is situated along the Mekong – Salween watershed. The Sittaung is an example of the medium-sized drainages, which are less resistant to human activities, e.g., dam construction, channelization and water pollution^58^. This unique basin should therefore be a focus of international conservation efforts alongside the largest Southeast Asian rivers.

## Methods

### Nomenclatural acts

The electronic edition of this article conforms to the requirements of the amended International Code of Zoological Nomenclature (ICZN), and hence the new names contained herein are available under that Code from the electronic edition of this article. This published work and the nomenclatural acts it contains have been registered in ZooBank (http://zoobank.org), the online registration system for the ICZN. The LSID for this publication is: urn:lsid:zoobank.org:pub:1CC25D33–7DF8–41D5–9817–5285C01A7779. The electronic edition of this paper was published in a journal with an ISSN, and has been archived and is available from PubMed Central.

### Studies of the type series of the Oriental taxa

The type specimens were studied in the malacological collections of the National Museum of Natural History, Smithsonian Institution, Washington, DC, USA (NMNH), the British Museum of Natural History, London, UK (NHMUK), and the Museo Civico di Storia Naturale di Genova, Genoa, Italy (MSNG). Additionally, we accessed the images of the types of several nominal taxa at the MUSSELp Database^59^.

### Taxon sampling and laboratory protocols

Our phylogeny of the Unionidae was based on 469 representatives of 138 in-group species from the Oriental Region (Indo-China and India), East Asia, Europe, Africa and North America (Supplementary Table 1). This sample includes all primary clades of the Unionidae, which were determined in the recent studies^9, 17^. The majority of these sequences were sampled from our work on the biogeography of the Oriental freshwater mussels^9^. New sequences were obtained from 74 specimens belonging to 15 species that were collected from the Sittaung and Irrawaddy river basins, and from a river of the Malay Peninsula (Supplementary Tables 1 and 2). All of our voucher specimens are deposited in RMBH, Russian Museum of Biodiversity Hotspots, the Federal Center for Integrated Arctic Research, Russian Academy of Sciences (Arkhangelsk, Russia). Total genomic DNA was extracted from 95% ethanol-preserved tissue samples using the NucleoSpin^®^ Tissue Kit (Macherey-Nagel GmbH & Co. KG, Germany), following the manufacturer’s protocol. For molecular analyses we obtained partial sequences of two mtDNA markers, i.e., the *cytochrome c oxidase* subunit I gene (COI) and the 16S ribosomal RNA (16S rRNA), and a fragment of the nuclear 28S ribosomal DNA (28S rDNA). Primer sequences for PCR are shown in Supplementary Table 4. Thermocycling was implemented with marker-specific PCR programs as follows: (i) COI: 95 °C (4 min), followed by 37 cycles at 94 °C (50 sec), 50 °C (50 sec), 72 °C (50 sec) and a final extension at 72 °C (5 min); (ii) 16S rRNA: 95 °C (4 min), followed by 33 cycles at 94 °C (50 sec), 47 °C (50 sec), 72 °C (50 sec) and a final extension at 72 °C (5 min); (iii) 28S rDNA: 95 °C (4 min), followed by 38 cycles at 94 °C (50 sec), 57 °C (50 sec), 72 °C (50 sec) and a final extension at 72 °C (5 min). Forward and reverse sequence reactions were performed directly on purified PCR products using the ABI PRISM^®^ BigDye™ Terminator v. 3.1 reagents kit and run on an ABI PRISM^®^ 3730 DNA analyzer (Thermo Fisher Scientific Inc., Waltham, MA, USA). The resulting sequences were checked by eye using a sequence alignment editor (BioEdit v. 7.2.5)^60^. A total of 25 mussel species were used as an out-group, including representatives of Margaritiferidae (10 species), Iridinidae (2 species), Etheriidae (1 species), Mycetopodidae (1 species), Hyriidae (9 species) and Trigoniidae (2 species) (Supplementary Table 1).

### Sequence alignment, checking the congruence of phylogenetic signals and substitution saturation analyses

The sequence alignment of COI, 16S rRNA and 28S rDNA gene fragments was performed separately using the Muscle algorithm implemented in MEGA6^61^. The aligned sequence data sets were checked through GBlocks v. 0.91b^62^ which allow to exclude hypervariable fragments from the sequence alignments using options for less stringent selection, enabling gap positions, smaller final blocks and less strict flanking positions. The resulting lengths of the sequence alignments are listed in Supplementary Table 5. To estimate each partition for evidence of substitution saturation, we performed the test of Xia *et al*.^63^ using DAMBE v. 5.3.108^64^, which showed little saturation even under the assumption of an asymmetrical tree (*P* < 0.001). A partition homogeneity test was calculated in PAUP* v. 4.0a151 to confirm the congruence of phylogenetic signals among sequence data sets^65^. This test revealed that the signals among the data sets are consistent (Supplementary Table 6).

### Phylogenetic analyses and divergence time estimates

The alignment data sets were joined in combined multi-gene nucleotide sequence alignments and collapsed into unique haplotypes (Supplementary Table 1) using an online FASTA sequence toolbox (FaBox 1.41)^66^. Absent sites were treated as missing data. For phylogenetic analyses, we used the resulting combined data set with unique haplotypes, including those that were possibly identical but differed by the availability of gene partitions for certain specimens.

In phylogenetic analyses, we tested only the dataset with five partitions (3 codons of COI + 16S rRNA + 28S rDNA), because Bolotov *et al*.^9^ show that the combined phylogeny of the Indo-Chinese Unionidae corresponds to those obtained from separate partitions. The ML phylogenetic analysis was conducted using RAxML v. 8.2.6 HPC Black Box^67^ at the San Diego Supercomputer Center through the CIPRES Science Gateway^68^. A unique GTR model was applied for each partition with corrections for gamma distribution. Nodal support values were estimated using an automatic, rapid bootstrapping algorithm according to the developer’s recommendation^67^, and the majority rule consensus tree was constructed from the independent searches. Bayesian inference (BI) analyses were performed in MrBayes v. 3.2.6^69^ at the San Diego Supercomputer Center through the CIPRES Science Gateway^68^. The data set tested was similar under the ML model. The best models of sequence evolution for each partition based on the corrected Akaike Information Criterion (AICc) of MEGA6^61^ are presented in Supplementary Table 8. Two runs, each with three heated (temperature = 0.1) and one cold Markov chain, were conducted for 15 million generations. Trees were sampled every 1000th generation. After completion of the MCMC analysis, the first 15% of trees were discarded as burn-in (pre-convergence part), and the majority rule consensus tree was calculated from the remaining trees. Convergence of the MCMC chains to a stationary distribution was checked visually based on the plotted posterior estimates using an MCMC trace analysis tool (Tracer v. 1.6)^70^. The effective sample size (ESS) value for each parameter sampled from the MCMC analysis was always recorded as >1300.

We estimated the acceptance of a global molecular clock to our multi-gene data set using the maximum likelihood test of MEGA6^61^, which revealed that the null hypothesis of equal evolutionary rate throughout the tree was rejected at a 5% significance level (*p* < 0.001). Thus, the time-calibrated haplotype-level Bayesian phylogeny was reconstructed in BEAST v. 1.8.4 based on multiple fossil calibration points using a lognormal relaxed clock algorithm with the Yule speciation process as the tree prior^71–73^. Calculations were performed at the San Diego Supercomputer Center through the CIPRES Science Gateway^68^. A fossil-calibrated ultrametric tree was obtained using BEAST v. 1.8.4. We specified similar settings to five partitions (3 codons of COI + 16S rRNA + 28S rDNA) as in the MrBayes analyses, but by using simplified evolutionary models. The HKY model was applied to each partition instead the GTR model, because the prior and posterior ESS values under the GTR model were recorded always <100. This indicates that the GTR model is likely overly complex for our data^9^. The eight published fossil calibrations were used for timing of the phylogeny^9^. We designated priors for out-group taxa using a “Monophyly” option of BEAUti v. 1.8.4^73^ as follows: (Trigoniidae, (Unionida)). Four replicate BEAST searches were conducted, each with 50 million generations. The trees were sampled every 5,000th generation. The log files were checked visually with Tracer v. 1.6 for an assessment of the convergence of the MCMC chains and the effective sample size of parameters^70^. The first 10–52% of trees were discarded as an appropriate burn-in. All the ESS values were recorded as >250, with exception of two parameters with the ESS >120; the posterior distributions were similar to the prior distributions. The resulting tree files from four independent analyses were compiled with LogCombiner v. 1.8.4^73^. The maximum clade credibility tree was obtained from 31,404 primary trees using TreeAnnotator v. 1.8.4^73^.

### Species delimitation analyses

The preliminary delimitation of biological species was based on a molecular approach using the MOTU concept^74–78^. MOTUs were separated based on the bPTP model^78^ to infer putative species boundaries on a phylogenetic input tree inferred from a maximum likelihood (ML) analysis. The ML analysis was conducted based on an alignment of the COI haplotype sequences of Parreysiinae and Pseudodontinae + Rectidentinae groups separately using RAxML v. 8.2.6 HPC Black Box^67^ at the San Diego Supercomputer Center through the CIPRES Science Gateway^68^. A unique GTR model was applied for each partition (three codons of COI) with corrections for gamma distribution. *Margaritifera laosensis* and *M*. *dahurica* were used as an out-group for the each group. We used an implementation of the bPTP model thorough online bPTP server (http://species.h-its.org/ptp) with 500,000 Markov Chain Monte Carlo (MCMC) generations and 10% burn-in^78^. All out-group taxa were removed from the input tree using an appropriate option of the server. The output parameters of the bPTP model for each clade under the highest Bayesian solution were as follows: (i) Parreysiinae: an estimated number of species = 40–88, mean value = 61.1, acceptance rate = 0.46; and (ii) Pseudodontinae + Rectidentinae: an estimated number of species = 45–69, mean value = 53.5, acceptance rate = 0.31. Additionally, MOTUs were obtained using the sPTP (p < 0.001) and mPTP models of Kapli *et al*.^79^ thorough online mPTP server (http://mptp.h-its.org). A phylogenetic input tree was obtained from ML analysis, which was conducted based on an alignment of the COI haplotype sequences of the Oriental Unionidae with the two haplotypes of *Margaritifera laosensis* and *M*. *dahurica* as an out-group using RAxML v. 8.2.6 HPC Black Box^67^.

In order to diagnose each new species, we followed a two-step procedure of Delić *et al*.^80^. Firstly, we estimate the morphological differences between a new species and closely related (congeneric) taxa. The comparative analysis of the shell morphology was carried out with attention to the structure of the pseudo-cardinal and lateral teeth, muscle attachment scars, shell shape and umbo position^25^. Secondly, the molecular diagnosis of each new species was provided using the fixed nucleotide differences^80–82^, which were estimated for each gene separately using a Toggle conserved sites tool of MEGA6 at 50% level^61^. For each species, an alignment of congeneric haplotype sequences was performed using the ClustalW algorithm implemented in MEGA6^61^. All the deleterious mutations were retained for the analyses. Additionally, a COI mean p-distance to the nearest neighbor of each species was calculated in MEGA6^61^.

### Data availability

The sequences generated under this study are available from GenBank. Accession numbers for each specimen are presented in Supplementary Table 1. The type specimens of the new species and voucher specimens of the other taxa that we studied are available in the RMBH, Russian Museum of Biodiversity Hotspots, the Federal Center for Integrated Arctic Research, Russian Academy of Sciences (Arkhangelsk, Russia).

## Electronic supplementary material

Supplementary Info
