# Supplementary material for: New taxa of freshwater mussels (Unionidae) from a species-rich but overlooked evolutionary hotspot in Southeast Asia
Source: Sci Rep. 2017 Sep 14;7:11573. doi: 10.1038/s41598-017-11957-9 (PMC5599626; doi:10.1038/s41598-017-11957-9)
Supplement: Supplementary file 1 — Supplementary Info [file 41598_2017_11957_MOESM1_ESM.pdf]

SUPPLEMENTARY INFORMATION

New taxa of freshwater mussels (Unionidae) from a species-rich but overlooked evolutionary hotspot in Southeast Asia

Ivan N. Bolotov*, Ilya V. Vikhrev, Alexander V. Kondakov, Ekaterina S. Konopleva, Mikhail Yu. Gofarov, Olga V. Aksenova and Sakboworn Tumpeesuwan

*Corresponding author: inepras@yandex.ru

Contents

Supplementary Table 1. List of sequences used in this study, including the species, the location and NCBI's GenBank accession numbers.

Supplementary Table 2. List of sampling localities in Indo-China

Supplementary Table 3. Habitats of the native Unionidae species from western Indo-China

Supplementary Table 4. Primer sequences for PCR amplification and sequencing

Supplementary Table 5. Alignment length prior to and after treatment for length variability in GBlocks v. 0.91b

Supplementary Table 6. Probability (p-value) of phylogenetic conflict among sequence data sets from a partition-homogeneity test implemented in PAUP* v. 4.0a150

Supplementary Table 7. Models of sequence evolution for each partition based on corrected Akaike Information Criterion (AICc) of MEGA6 that were applied within a Bayesian inference framework

Supplementary Figure 1. Majority rule consensus phylogenetic tree of the Unionidae recovered from BI analysis of the complete data set of mitochondrial and nuclear sequences (five partitions: three codons of COI + 16S rRNA + 28S rDNA)

Supplementary Figure 2. Fossil-calibrated ultrametric chronogram calculated under a lognormal relaxed clock model and a Yule process speciation implemented in BEAST 1.8.4 and obtained for the complete data set of mitochondrial and nuclear sequences (five partitions: three codons of COI + 16S rRNA + 28S rDNA)

Supplementary Figure 3. Shell variability of *Lamellidens generosus* (Gould, 1847) from Lake Inle and surrounding water bodies.

Supplementary Table 1. List of sequences used in this study, including the species, the location and NCBI's GenBank accession numbers. Range Codes are as follows: (WI) Western Indo-China: Irrawaddy, Sittaung, Salween, Tavoy, and Kaladan catchments; (IN) rivers of the Indian subcontinent; (PM) Paleo-Mekong basin: Mekong and Chao Phraya catchments, and rivers of the Malay Peninsula; (AF) tropical Africa: Congo, Zambezi, and Nile drainage basins; (EA) East Asia (from the Red River catchment to Far Eastern Russia); (EU) Europe; and (NA) North America.

Taxa	Locality	Range Code	Specimen Voucher*	Haplotype Code	COI	16S rRNA	28S rDNA	Reference
In-Group Taxa								
UNIONIDAE Rafinesque, 1820								
PARREYSIINAE Henderson 1935								
Lamellidentini Modell, 1942								
Trapezidens exolescens (Gould, 1843) **comb. nov.**	Myanmar: Tavoy River	WI	RMBH: biv_145_3	Hap129	KX230532	KX230548	KX230559	Ref. 9
Trapezidens exolescens (Gould, 1843) **comb. nov.**	Myanmar: Tavoy River	WI	RMBH: biv_145_21	Hap129	KX230536	KX230550	KX230561	Ref. 9
Trapezidens exolescens (Gould, 1843) **comb. nov.**	Myanmar: Tavoy River	WI	RMBH: biv_146_2	Hap129	KX230537	KX230551	KX230562	Ref. 9
Trapezidens exolescens (Gould, 1843) **comb. nov.**	Myanmar: Tavoy River	WI	RMBH: biv_146_14	Hap129	KX230540	KX230552	KX230563	Ref. 9
Trapezidens exolescens (Gould, 1843) **comb. nov.**	Myanmar: Tavoy River	WI	RMBH: biv_145_12	Hap130	KX230535	KX230549	KX230560	Ref. 9
Trapezidens exolescens (Gould, 1843) **comb. nov.**	Myanmar: Tavoy River	WI	RMBH: biv_146_23	Hap131	KX230542	KX230553	KX230564	Ref. 9
Trapezidens obesa feae **ssp. nov.**	Myanmar: Sittaung River basin, Myit Kyi Pauk Stream	WI	RMBH: biv_250_4	Hap307	MF352238	MF352308	MF352366	Present study
Trapezidens obesa feae **ssp. nov.**	Myanmar: Sittaung River basin, Myit Kyi Pauk Stream	WI	RMBH: biv_250_7	Hap307	MF352239	MF352309	MF352367	Present study
Trapezidens obesa feae **ssp. nov.**	Myanmar: Sittaung River basin, Myit Kyi Pauk Stream	WI	RMBH: biv_250_8	Hap307	MF352240	MF352310	MF352368	Present study
Trapezidens obesa feae **ssp. nov.**	Myanmar: Sittaung River near Taungoo	WI	RMBH: biv_255_1	Hap307	MF352253	MF352320	MF352378	Present study
Trapezidens obesa obesa (Hanley et Theobald, 1876)	Myanmar: Irrawaddy River	WI	UA: 20729 / UMMZ: 304642	Hap164	JN243903	KP795042	JN243881	Ref. 53, 54
Lamellidens savadiensis (Nevill, 1877) **stat. res.**	Myanmar: Irrawaddy River basin, Lake Indawgyi	WI	RMBH: biv_109_1	Hap070	KX230544	KX230555	KX230566	Ref. 9
Lamellidens savadiensis (Nevill, 1877) **stat. res.**	Myanmar: Irrawaddy River basin, Lake Indawgyi	WI	RMBH: biv_109_2	Hap071	KX230545	KX230556	KX230567	Ref. 9
Lamellidens savadiensis (Nevill, 1877) **stat. res.**	Myanmar: Irrawaddy River basin, Lake Indawgyi	WI	RMBH: biv_109_3	Hap072	KX230546	KX230557	KX230568	Ref. 9
Lamellidens savadiensis (Nevill, 1877) **stat. res.**	Myanmar: Irrawaddy River basin, Chindwin River: Paukin Lake	WI	UA:20727.1/UMMZ :MC:304346	Hap167	JN243902	KF011263	JN243880	Ref. 53, 54

Taxa	Locality	Range Code	Specimen Voucher*	Haplotype Code	COI	16S rRNA	28S rDNA	Reference
Lamellidens savadiensis (Nevill, 1877) **stat. res.**	Myanmar: Sittaung River basin, Pathi River	WI	RMBH: biv_242_15	Hap296	MF352221	MF352295	MF352353	Present study
Lamellidens savadiensis (Nevill, 1877) **stat. res.**	Myanmar: Sittaung River basin, Pathi River	WI	RMBH: biv_242_8	Hap296	MF352220	MF352294	MF352352	Present study
Lamellidens savadiensis (Nevill, 1877) **stat. res.**	Myanmar: Sittaung River basin, fishing pond	WI	RMBH: biv_247_14	Hap296	MF352233	n/a	n/a	Present study
Lamellidens savadiensis (Nevill, 1877) **stat. res.**	Myanmar: Sittaung River basin, fishing pond	WI	RMBH: biv_247_8	Hap296	MF352231	MF352303	MF352361	Present study
Lamellidens savadiensis (Nevill, 1877) **stat. res.**	Myanmar: Irrawaddy River basin, Lake Nant Phar	WI	RMBH: biv_257_1	Hap316	MF352258	MF352322	MF352383	Present study
Lamellidens savadiensis (Nevill, 1877) **stat. res.**	Myanmar: Irrawaddy River basin, Lake Nant Phar	WI	RMBH: biv_257_6	Hap317	MF352259	MF352323	MF352384	Present study
Lamellidens savadiensis (Nevill, 1877) **stat. res.**	Myanmar: Irrawaddy River basin, Lake Nant Phar	WI	RMBH: biv_257_9	Hap318	MF352260	MF352324	MF352385	Present study
Lamellidens savadiensis (Nevill, 1877) **stat. res.**	Myanmar: Irrawaddy River basin, Lake Shwe Kyi	WI	RMBH: biv_261_3	Hap316	MF352269	MF352333	MF352393	Present study
Lamellidens savadiensis (Nevill, 1877) **stat. res.**	Myanmar: Irrawaddy River basin, Lake Shwe Kyi	WI	RMBH: biv_261_6	Hap316	MF352270	MF352334	MF352394	Present study
Lamellidens savadiensis (Nevill, 1877) **stat. res.**	Myanmar: Irrawaddy River basin, Lake Shwe Kyi	WI	RMBH: biv_261_7	Hap318	MF352271	n/a	n/a	Present study
Lamellidens savadiensis (Nevill, 1877) **stat. res.**	Myanmar: Irrawaddy River basin, Nant Sa Yi River	WI	RMBH: biv_262_1	Hap324	MF352272	MF352335	MF352395	Present study
Lamellidens savadiensis (Nevill, 1877) **stat. res.**	Myanmar: Irrawaddy River basin, Nant Sa Yi River	WI	RMBH: biv_262_2	Hap325	MF352273	n/a	n/a	Present study
Lamellidens savadiensis (Nevill, 1877) **stat. res.**	Myanmar: Irrawaddy River basin, Nant Sa Yi River	WI	RMBH: biv_262_4	Hap326	MF352274	MF352336	MF352396	Present study
Lamellidens savadiensis (Nevill, 1877) **stat. res.**	Myanmar: Irrawaddy River basin, Lake Myaung	WI	RMBH: biv_264_1	Hap328	MF352277	MF352339	MF352398	Present study
Lamellidens savadiensis (Nevill, 1877) **stat. res.**	Myanmar: Irrawaddy River basin, Lake Myaung	WI	RMBH: biv_264_4	Hap316	MF352278	n/a	n/a	Present study
Lamellidens savadiensis (Nevill, 1877) **stat. res.**	Myanmar: Irrawaddy River basin, Lake Myaung	WI	RMBH: biv_264_7	Hap328	MF352279	MF352340	MF352399	Present study
Lamellidens brandti **sp. nov.**	Myanmar: Sittaung River basin, Pathi river	WI	RMBH: biv_242_3	Hap295	MF352219	MF352293	MF352351	Present study
Lamellidens brandti **sp. nov.**	Myanmar: Sittaung River basin, Pathi river	WI	RMBH: biv_243_10	Hap297	MF352222	MF352296	MF352354	Present study
Lamellidens brandti **sp. nov.**	Myanmar: Sittaung River basin, Pathi river	WI	RMBH: biv_243_12	Hap295	MF352223	MF352297	MF352355	Present study
Lamellidens brandti **sp. nov.**	Myanmar: Sittaung River basin, Pathi river	WI	RMBH: biv_243_14	Hap298	MF352224	n/a	n/a	Present study
Lamellidens brandti **sp. nov.**	Myanmar: Sittaung River basin, reservoir of Yetho River	WI	RMBH: biv_244_2	Hap299	MF352225	n/a	n/a	Present study
Lamellidens brandti **sp. nov.**	Myanmar: Sittaung River basin, reservoir of Yetho River	WI	RMBH: biv_244_3	Hap299	MF352226	MF352298	MF352356	Present study
Lamellidens brandti **sp. nov.**	Myanmar: Sittaung River basin, reservoir of Yetho River	WI	RMBH: biv_244_5	Hap300	MF352227	MF352299	MF352357	Present study
Lamellidens brandti **sp. nov.**	Myanmar: Sittaung River basin, fishing pond	WI	RMBH: biv_247_10	Hap303	MF352232	MF352304	MF352362	Present study

Taxa	Locality	Range Code	Specimen Voucher*	Haplotype Code	COI	16S rRNA	28S rDNA	Reference
Lamellidens brandti **sp. nov.**	Myanmar: Sittaung River near Taungoo	WI	RMBH: biv_250_13	Hap298	MF352241	MF352311	MF352369	Present study
Lamellidens generosus (Gould, 1847)	Myanmar: Salween River basin, Lake Inle	WI	RMBH: biv_113_8	Hap042	KX865825	KX865596	KX865699	Ref. 9
Lamellidens generosus (Gould, 1847)	Myanmar: Salween River basin, Lake Inle	WI	RMBH: biv_113_12	Hap043	KX865826	KX865597	KX865700	Ref. 9
Lamellidens generosus (Gould, 1847)	Myanmar: Salween River basin, Lake Inle	WI	RMBH: biv_113_14	Hap044	KX865827	KX865598	KX865701	Ref. 9
Lamellidens generosus (Gould, 1847)	Myanmar: Salween River basin, Lake Inle, a channel in Nuangshve	WI	RMBH: biv_112_7	Hap106	KX865828	KX865599	KX865702	Ref. 9
Lamellidens generosus (Gould, 1847)	Myanmar: Salween River basin, Lake Inle, a channel in Nuangshve	WI	RMBH: biv_112_12	Hap106	KX865829	KX865600	KX865703	Ref. 9
Lamellidens generosus (Gould, 1847)	Myanmar: Salween River basin, Lake Inle, a channel in Nuangshve	WI	RMBH: biv_112_15	Hap106	KX865830	KX865601	KX865704	Ref. 9
Lamellidens generosus (Gould, 1847)	Myanmar: Salween River basin, Nam Pilu River	WI	RMBH: biv_141_5	Hap123	KX865831	KX865602	KX865705	Ref. 9
Lamellidens generosus (Gould, 1847)	Myanmar: Salween River basin, Nam Pilu River	WI	RMBH: biv_141_9	Hap124	KX865832	KX865603	KX865706	Ref. 9
Lamellidens generosus (Gould, 1847)	Myanmar: Salween River basin, Nam Pilu River	WI	RMBH: biv_141_18	Hap125	KX865833	KX865604	KX865707	Ref. 9
Lamellidens generosus (Gould, 1847)	Myanmar: Salween River basin, Snake Stream	WI	RMBH: biv_142_1	Hap125	KX865834	KX865605	KX865708	Ref. 9
Lamellidens aff. *marginalis* (Lamarck, 1819) sp.1	India	IN	SBM12	Hap169	KF690121	n/a	n/a	GenBank
Lamellidens aff. *marginalis* (Lamarck, 1819) sp.1	India	IN	SBM16	Hap358	KF690118	n/a	n/a	GenBank
Lamellidens aff. *marginalis* (Lamarck, 1819) sp.1	India: Krishna River	IN	RLm3	Hap336	KP268833	n/a	n/a	GenBank
Lamellidens aff. *marginalis* (Lamarck, 1819) sp.1	India: Krishna River	IN	RLm4	Hap336	KP268834	n/a	n/a	GenBank
Lamellidens aff. *marginalis* (Lamarck, 1819) sp.1	India	IN	SBM2	Hap166	KF690119	n/a	n/a	GenBank
Lamellidens aff. *marginalis* (Lamarck, 1819) sp.2	India: Karli River basin, Bangsaal River	IN	RNBI19	Hap170	JQ861227	n/a	n/a	GenBank
Lamellidens aff. *marginalis* (Lamarck, 1819) sp.2	India: Karli River basin, Bangsaal River	IN	RNBI21	Hap346	JQ861228	n/a	n/a	GenBank
Lamellidens aff. *marginalis* (Lamarck, 1819) sp.3	Myanmar: Kaladan River basin, unnamed puddle	WI	RMBH: biv_153	Hap136	KX230547	KX230558	KX230569	Ref. 9
Lamellidens aff. *marginalis* (Lamarck, 1819) sp.4	India	IN	NBFGR:LMN5	Hap339	GQ149471	n/a	n/a	GenBank
Lamellidens aff. *marginalis* (Lamarck, 1819) sp.4	India	IN	NBFGR:LMN7	Hap340	GQ149472	n/a	n/a	GenBank
Lamellidens aff. *marginalis* (Lamarck, 1819) sp.4	India	IN	NBFGR:LMO26	Hap341	GQ149474	n/a	n/a	GenBank
Lamellidens aff. *marginalis* (Lamarck, 1819) sp.4	India	IN	NBFGR:LMO42	Hap342	GQ149475	n/a	n/a	GenBank
Lamellidens aff. *marginalis* (Lamarck, 1819) sp.5	India	IN	SBM24	Hap355	KF690108	n/a	n/a	GenBank

Taxa	Locality	Range Code	Specimen Voucher*	Haplotype Code	COI	16S rRNA	28S rDNA	Reference
Lamellidens aff. *marginalis* (Lamarck, 1819) sp.5	India	IN	SBM9	Hap359	KF690120	n/a	n/a	GenBank
Lamellidens cf. *corrianus* (Lea, 1834)	India: Karli River basin, unnamed stream	IN	RNBI25	Hap165	JQ861226	n/a	n/a	GenBank
Lamellidens cf. *corrianus* (Lea, 1834)	India: Krishna River basin, Koyna River	IN	RLc1	Hap377	KT869147	n/a	n/a	GenBank
Lamellidens cf. *corrianus* (Lea, 1834)	India: Krishna River basin, Ghataprabha River	IN	RNBI22	Hap344	JQ861223	n/a	n/a	GenBank
Lamellidens cf. *corrianus* (Lea, 1834)	India: Daman Ganga River basin, Wagh River	IN	RNBI27	Hap332	JQ861224	n/a	n/a	GenBank
Lamellidens cf. *corrianus* (Lea, 1834)	India: Krishna River basin, Ghataprabha River	IN	RNBI26	Hap345	JQ861225	n/a	n/a	GenBank
Lamellidens cf. *corrianus* (Lea, 1834)	India: Karli River basin, Bangsaal River	IN	RNBI18	Hap332	JQ861243	n/a	n/a	GenBank
Lamellidens cf. *jenkinsianus* (Benson, 1862)	India	IN	SBM8	Hap168	KF690117	n/a	n/a	GenBank
Lamellidens cf. *jenkinsianus* (Benson, 1862)	India: Krishna River	IN	RLd1	Hap371	KP268835	n/a	n/a	GenBank
Lamellidens cf. *jenkinsianus* (Benson, 1862)	India: Krishna River	IN	RLd2	Hap372	KP268836	n/a	n/a	GenBank
Lamellidens cf. *jenkinsianus* (Benson, 1862)	India: Godavari River	IN	RLd3	Hap376	KT869146	n/a	n/a	GenBank
Lamellidens cf. *jenkinsianus* (Benson, 1862)	India	IN	NBFGR:LMK14	Hap338	GQ149468	n/a	n/a	GenBank
Lamellidens cf. *jenkinsianus* (Benson, 1862)	India	IN	NBFGR:LMK15	Hap338	GQ149469	n/a	n/a	GenBank
Lamellidens cf. *jenkinsianus* (Benson, 1862)	India	IN	NBFGR:LMK25	Hap338	GQ149470	n/a	n/a	GenBank
Lamellidens cf. *jenkinsianus* (Benson, 1862)	India	IN	NBFGR:LMN34	Hap333	GQ149473	n/a	n/a	GenBank
Lamellidens cf. *jenkinsianus* (Benson, 1862)	India	IN	SBM17	Hap356	KF690112	n/a	n/a	GenBank
Lamellidens cf. *jenkinsianus* (Benson, 1862)	India	IN	SBM10	Hap357	KF690113	n/a	n/a	GenBank
Lamellidens cf. *jenkinsianus* (Benson, 1862)	India	IN	SBM4	Hap376	KF690114	n/a	n/a	GenBank
Lamellidens cf. *jenkinsianus* (Benson, 1862)	India	IN	SBM5	Hap376	KF690115	n/a	n/a	GenBank
Lamellidens cf. *jenkinsianus* (Benson, 1862)	India	IN	SBM28	Hap357	KF690116	n/a	n/a	GenBank
Parreysiini Henderson, 1935								
Parreysia cf. *corrugata* (Müller, 1774) sp.1	India: Periyar River	IN	RNB1	Hap334	KJ872809	n/a	n/a	GenBank
Parreysia cf. *corrugata* (Müller, 1774) sp.1	India: Periyar River	IN	RNB2	Hap334	KJ872810	n/a	n/a	GenBank
Parreysia cf. *corrugata* (Müller, 1774) sp.1	India: Periyar River	IN	RCB2	Hap363	KJ872811	n/a	n/a	GenBank

Taxa	Locality	Range Code	Specimen Voucher*	Haplotype Code	COI	16S rRNA	28S rDNA	Reference
Parreysia cf. *corrugata* (Müller, 1774) sp.1	India: Periyar River	IN	RCB3	Hap363	KJ872812	n/a	n/a	GenBank
Parreysia cf. *corrugata* (Müller, 1774) sp.1	India: Periyar River	IN	RCB4	Hap363	KJ872813	n/a	n/a	GenBank
Parreysia cf. *corrugata* (Müller, 1774) sp.1	India: Periyar River	IN	RCB5	Hap363	KJ872814	n/a	n/a	GenBank
Parreysia cf. *corrugata* (Müller, 1774) sp.1	India: Periyar River	IN	RCB8	Hap364	KJ872815	n/a	n/a	GenBank
Parreysia cf. *corrugata* (Müller, 1774) sp.1	India: Periyar River	IN	RCB9	Hap365	KJ872816	n/a	n/a	GenBank
Parreysia cf. *corrugata* (Müller, 1774) sp.1	India: Periyar River	IN	RCB12	Hap366	KJ872817	n/a	n/a	GenBank
Parreysia cf. *corrugata* (Müller, 1774) sp.1	India: Pamba River basin, Achankovil River	IN	RPc10	Hap373	KT869140	n/a	n/a	GenBank
Parreysia cf. *corrugata* (Müller, 1774) sp.2	India: Krishna River basin, Ghataprabha River	IN	RNBI1	Hap140	JQ861229	n/a	n/a	GenBank
Parreysia cf. *corrugata* (Müller, 1774) sp.2	India: Karli River basin, Pitdhaval River	IN	RNBI14	Hap141	JQ861230	n/a	n/a	GenBank
Parreysia cf. *corrugata* (Müller, 1774) sp.2	India: Tapi River	IN	RNBI13	Hap347	JQ861231	n/a	n/a	GenBank
Parreysia cf. *corrugata* (Müller, 1774) sp.2	India: Ulhas River basin	IN	RNBI11	Hap141	JQ861232	n/a	n/a	GenBank
Parreysia cf. *corrugata* (Müller, 1774) sp.2	India: Krishna River basin, Ghataprabha River	IN	RNBI6	Hap349	JQ861233	n/a	n/a	GenBank
Parreysia cf. *corrugata* (Müller, 1774) sp.2	India: Krishna River basin, Ghataprabha River	IN	RNBI5	Hap140	JQ861234	n/a	n/a	GenBank
Parreysia cf. *corrugata* (Müller, 1774) sp.2	India: Krishna River basin, Ghataprabha River	IN	RNBI4	Hap140	JQ861235	n/a	n/a	GenBank
Parreysia cf. *corrugata* (Müller, 1774) sp.2	India: Krishna River basin, Ghataprabha River	IN	RNBI3	Hap351	JQ861236	n/a	n/a	GenBank
Parreysia cf. *corrugata* (Müller, 1774) sp.2	India: Krishna River basin, Ghataprabha River	IN	RNBI2	Hap140	JQ861237	n/a	n/a	GenBank
Parreysia cf. *corrugata* (Müller, 1774) sp.2	India: Krishna River, Nagarjuna Sagar	IN	RPf2	Hap369	KP268829	n/a	n/a	GenBank
Parreysia cf. *corrugata* (Müller, 1774) sp.2	India	IN	SBM3	Hap144	KF690110	n/a	n/a	GenBank
Parreysia cf. *corrugata* (Müller, 1774) sp.2	India	IN	SBM27	Hap145	KF690111	n/a	n/a	GenBank
Parreysia cf. *corrugata* (Müller, 1774) sp.2	India: Krishna River	IN	RPf1	Hap368	KP268828	n/a	n/a	GenBank
Parreysia cf. *corrugata* (Müller, 1774) sp.2	India: Krishna River, Nagarjuna Sagar	IN	RPf3	Hap370	KP268830	n/a	n/a	GenBank
Parreysia cf. *corrugata* (Müller, 1774) sp.2	India: Godavari River	IN	RPf4	Hap375	KT869142	n/a	n/a	GenBank
Parreysia cf. *corrugata* (Müller, 1774) sp.3	India: Suk river	IN	RNBI12	Hap352	JQ861238	n/a	n/a	GenBank
Parreysia cf. *corrugata* (Müller, 1774) sp.3	India: Karli River basin, Pitdhaval River	IN	RNBI18	Hap143	JQ861239	n/a	n/a	GenBank

Taxa	Locality	Range Code	Specimen Voucher*	Haplotype Code	COI	16S rRNA	28S rDNA	Reference
Parreysia cf. *corrugata* (Müller, 1774) sp.3	India: Karli River basin, Pitdhaval River	IN	RNBI17	Hap142	JQ861240	n/a	n/a	GenBank
Parreysia cf. *corrugata* (Müller, 1774) sp.3	India: Pinddhaval River	IN	RNBI16	Hap353	JQ861241	n/a	n/a	GenBank
Parreysia cf. *corrugata* (Müller, 1774) sp.3	India: Pinddhaval River	IN	RNBI15	Hap354	JQ861242	n/a	n/a	GenBank
Leoparreysiini Vikhrev, Bolotov et Kondakov **tribe nov.**								
Leoparreysia olivacea (Prashad, 1930) **comb. nov.**	Myanmar: Irrawaddy River	WI	UMMZ:304641	Hap139	KP795022	KP795044	KP795005	Ref. 54
Leoparreysia canefrii **sp. nov.**	Myanmar: Sittaung River near Taungoo	WI	RMBH: biv_249	Hap306	MF352237	MF352307	MF352365	Present study
Leoparreysia canefrii **sp. nov.**	Myanmar: Sittaung River near Taungoo	WI	RMBH: biv_252_1	Hap309	MF352245	MF352315	MF352373	Present study
Leoparreysia canefrii **sp. nov.**	Myanmar: Sittaung River near Taungoo	WI	RMBH: biv_252_2	Hap310	MF352246	MF352316	MF352374	Present study
Leoparreysia canefrii **sp. nov.**	Myanmar: Sittaung River near Taungoo	WI	RMBH: biv_252_3	Hap311	MF352247	n/a	n/a	Present study
Leoparreysia canefrii **sp. nov.**	Myanmar: Sittaung River near Taungoo	WI	RMBH: biv_254_2	Hap310	MF352250	MF352319	MF352377	Present study
Leoparreysia canefrii **sp. nov.**	Myanmar: Sittaung River near Taungoo	WI	RMBH: biv_254_4	Hap313	MF352251	n/a	n/a	Present study
Leoparreysia canefrii **sp. nov.**	Myanmar: Sittaung River near Taungoo	WI	RMBH: biv_254_6	Hap309	MF352252	n/a	n/a	Present study
Leoparreysia tavoyensis (Gould, 1843) **comb. nov.**	Myanmar: Tavoy River	WI	RMBH: biv_149	Hap135	KX230543	KX230554	KX230565	Ref. 9
Leoparreysia bhamoensis (Theobald, 1873) **comb. nov.** (=*Unio mandelayensis* Theobald, 1873 **syn. nov.**)	Myanmar: Irrawaddy River basin, Lake Myaung	WI	RMBH: biv_266_4	Hap329	MF352280	MF352343	MF352400	Present study
Leoparreysia bhamoensis (Theobald, 1873) **comb. nov.** (=*Unio mandelayensis* Theobald, 1873 **syn. nov.**)	Myanmar: Irrawaddy River basin, Lake Myaung	WI	RMBH: biv_266_5	Hap329	MF352281	MF352344	MF352401	Present study
Leoparreysia bhamoensis (Theobald, 1873) **comb. nov.** (=*Unio mandelayensis* Theobald, 1873 **syn. nov.**)	Myanmar: Irrawaddy River basin, Lake Myaung	WI	RMBH: biv_266_6	Hap329	MF352282	MF352345	MF352402	Present study
Leoparreysia bhamoensis (Theobald, 1873) **comb. nov.** (=*Unio mandelayensis* Theobald, 1873 **syn. nov.**)	Myanmar: Irrawaddy River	WI	UA20726.1/ UMMZ:304640	Hap137	JN243901	KP795043	JN243877	Ref. 53, 54
Leoparreysia bhamoensis (Theobald, 1873) **comb. nov.** (=*Unio mandelayensis* Theobald, 1873 **syn. nov.**)	Myanmar: Irrawaddy River	WI	UA: 20722.1	Hap138	JN243900	n/a	JN243876	Ref. 53
Coelaturini Modell, 1942								

Taxa	Locality	Range Code	Specimen Voucher*	Haplotype Code	COI	16S rRNA	28S rDNA	Reference
Nitia teretiuscula (Philippi, 1847)	Egypt: Nile River	AF	ANSP:416305/UA:20993.1	Hap173	JN243897	n/a	JN243875	Ref. 53
Nitia teretiuscula (Philippi, 1847)	Egypt: Nile River	AF	ANSP:416305	Hap174	KJ081160	n/a	JN243875	Ref. 85
Coelatura gabonensis (Küster, 1862)	Congo River	AF	ANSP:A21417/ UA:21018.1	Hap156	JN243895	n/a	JN243873	Ref. 53
Coelatura mweruensis (Smith, 1908)	Zambia: Congo River basin, Lake Mweru	AF	UMMZ:304377	Hap161	KJ081174	n/a	KJ081189	Ref. 85
Coelatura mweruensis (Smith, 1908)	Zambia: Congo River basin, Lake Bangweulu	AF	ANSP:416276	Hap157	KJ081167	n/a	KJ081186	Ref. 85
Coelatura mweruensis (Smith, 1908)	Zambia: Congo River basin, Lake Bangweulu	AF	ANSP:416276	Hap158	KJ081168	n/a	KJ081187	Ref. 85
Coelatura mweruensis (Smith, 1908)	Zambia: Congo River basin, Chembeshi River basin, Kalungu River	AF	ANSP:416247	Hap159	KJ081169	n/a	KJ081188	Ref. 85
Coelatura mweruensis (Smith, 1908)	Zambia: Congo River basin, Chembeshi River basin, Kalungu River	AF	ANSP:416247	Hap160	KJ081170	n/a	KJ081188	Ref. 85
Coelatura mweruensis (Smith, 1908)	Zambia: Congo River basin, Lake Mweru	AF	UMMZ:304377	Hap162	KJ081175	n/a	KJ081190	Ref. 85
Coelatura mweruensis (Smith, 1908)	Zambia: Congo River basin, Lake Mweru	AF	UMMZ:304377	Hap163	KJ081176	n/a	KJ081191	Ref. 85
Coelatura mweruensis (Smith, 1908)	Zambia: Congo River basin, Lake Mweru	AF	UMMZ:304379	Hap171	KJ081180	n/a	KJ081193	Ref. 85
Coelatura mweruensis (Smith, 1908)	Zambia: Congo River basin, Lake Mweru	AF	ANSP:416363/UA:20834.1	Hap172	JN243896	n/a	JN243874	Ref. 53
Coelatura kunenensis (Mousson, 1887)	Zambia: Zambezi River	AF	ANSP:419411	Hap154	KJ081164	n/a	KJ081184	Ref. 85
Coelatura kunenensis (Mousson, 1887)	Zambia: Zambezi River	AF	ANSP:419411	Hap155	KJ081165	n/a	KJ081185	Ref. 85
Coelatura aff. *aegyptiaca* (Cailliaud, 1827) sp.1	Egypt: Nile River	AF	ANSP:416304	Hap152	JN243894	KP795045	JN243872	Ref. 53, 54
Coelatura aff. *aegyptiaca* (Cailliaud, 1827) sp.2	Egypt: Nile River	AF	ANSP:416304	Hap153	JN243892	n/a	JN243870	Ref. 53
Oxynaiini Starobogatov, 1970								
Indonaia caerulea (Lea, 1831) **comb. res.**	India: Krishna River basin, fish pond	IN	RRc1	Hap374	KT869141	n/a	n/a	GenBank
Indonaia andersoniana (Nevill, 1877) **comb. res.**	Myanmar: Irrawaddy River basin, Lake Indawgyi	WI	RMBH: biv_108 _1	Hap098	KX865835	KX865606	KX865709	Ref. 9
Indonaia andersoniana (Nevill, 1877) **comb. res.**	Myanmar: Irrawaddy River basin, Lake Indawgyi	WI	RMBH: biv_108 _2	Hap099	KX865836	KX865607	n/a	Ref. 9
Indonaia andersoniana (Nevill, 1877) **comb. res.**	Myanmar: Irrawaddy River basin, Lake Indawgyi	WI	RMBH: biv_108 _3	Hap100	KX865837	KX865608	n/a	Ref. 9
Indonaia andersoniana (Nevill, 1877) **comb. res.**	Myanmar: Irrawaddy River basin, Lake Nant Phar	WI	RMBH: biv_259_1	Hap321	MF352263	MF352327	MF352387	Present study
Indonaia andersoniana (Nevill, 1877) **comb. res.**	Myanmar: Irrawaddy River basin, Lake Nant Phar	WI	RMBH: biv_259_3	Hap321	MF352264	MF352328	n/a	Present study
Indonaia andersoniana (Nevill, 1877) **comb. res.**	Myanmar: Irrawaddy River basin, Lake Nant Phar	WI	RMBH: biv_259_4	Hap321	MF352265	MF352329	MF352388	Present study

Taxa	Locality	Range Code	Specimen Voucher*	Haplotype Code	COI	16S rRNA	28S rDNA	Reference
Indonaia andersoniana (Nevill, 1877) **comb. res.**	Myanmar: Irrawaddy River basin, Nant Sa Yi River	WI	RMBH: biv_263_1	Hap327	MF352275	MF352337	MF352397	Present study
Indonaia andersoniana (Nevill, 1877) **comb. res.**	Myanmar: Irrawaddy River basin, Nant Sa Yi River	WI	RMBH: biv_263_2	Hap327	MF352276	MF352338	n/a	Present study
Indonaia andersoniana (Nevill, 1877) **comb. res.**	Myanmar: Irrawaddy River basin, Lake Myaung	WI	RMBH: biv_267_1	Hap327	MF352283	MF352341	n/a	Present study
Indonaia andersoniana (Nevill, 1877) **comb. res.**	Myanmar: Irrawaddy River basin, Lake Myaung	WI	RMBH: biv_267_3	Hap327	MF352284	MF352342	n/a	Present study
Indonaia aff. *khadakvaslaensis* (Ray, 1966) sp.1	India	IN	SBM7	Hap148	KF690124	n/a	n/a	GenBank
Indonaia aff. *khadakvaslaensis* (Ray, 1966) sp.1	India: Godavari River	IN	RR1	Hap337	KT869143	n/a	n/a	GenBank
Indonaia aff. *khadakvaslaensis* (Ray, 1966) sp.1	India: Godavari River	IN	RR2	Hap337	KT869144	n/a	n/a	GenBank
Indonaia aff. *khadakvaslaensis* (Ray, 1966) sp.1	India: Godavari River	IN	RR3	Hap337	KT869145	n/a	n/a	GenBank
Indonaia aff. *khadakvaslaensis* (Ray, 1966) sp.1	India	IN	SBM19	Hap360	KF690122	n/a	n/a	GenBank
Indonaia aff. *khadakvaslaensis* (Ray, 1966) sp.2	India	IN	SBM15	Hap149	KF690107	n/a	n/a	GenBank
Indonaia aff. *khadakvaslaensis* (Ray, 1966) sp.3	India: Karli River basin, Bangsaal River	IN	RNBI7	Hap147	JQ861244	n/a	n/a	GenBank
Indonaia aff. *khadakvaslaensis* (Ray, 1966) sp.4	India: Krishna River basin, Ghataprabha River	IN	RNBI24	Hap343	JQ861221	n/a	n/a	GenBank
Indonaia lima (Simpson, 1900) **comb. res.**	India: Krishna River, Nagarjuna Sagar	IN	RRI1	Hap367	KP268827	n/a	n/a	GenBank
Radiatula mouhoti **sp. nov.**	Myanmar: Sittaung River near Taungoo	WI	RMBH: biv_248_1	Hap304	MF352234	MF352305	MF352363	Present study
Radiatula mouhoti **sp. nov.**	Myanmar: Sittaung River near Taungoo	WI	RMBH: biv_248_3	Hap304	MF352235	n/a	n/a	Present study
Radiatula mouhoti **sp. nov.**	Myanmar: Sittaung River near Taungoo	WI	RMBH: biv_248_4	Hap305	MF352236	MF352306	MF352364	Present study
Radiatula mouhoti **sp. nov.**	Myanmar: Sittaung River near Taungoo	WI	RMBH: biv_253_1	Hap312	MF352248	MF352317	MF352375	Present study
Radiatula mouhoti **sp. nov.**	Myanmar: Sittaung River near Taungoo	WI	RMBH: biv_253_6	Hap305	MF352249	MF352318	MF352376	Present study
Radiatula mouhoti **sp. nov.**	Myanmar: Sittaung River near Taungoo	WI	RMBH: biv_256	Hap315	MF352257	MF352321	MF352382	Present study
Radiatula cf. *bonneaudii* (Eydoux, 1838) sp.1	Myanmar: Irrawaddy River basin, Tar Pein River	WI	RMBH: biv_260_5	Hap322	MF352266	MF352330	MF352390	Present study
Radiatula cf. *bonneaudii* (Eydoux, 1838) sp.1	Myanmar: Irrawaddy River basin, Tar Pein River	WI	RMBH: biv_260_9	Hap322	MF352267	MF352331	MF352391	Present study
Radiatula cf. *bonneaudii* (Eydoux, 1838) sp.1	Myanmar: Irrawaddy River basin, Tar Pein River	WI	RMBH: biv_260_10	Hap323	MF352268	MF352332	MF352392	Present study
Radiatula cf. *bonneaudii* (Eydoux, 1838) sp.1	Myanmar: Irrawaddy River	WI	UA:20714.2/UMMZ : 304645	Hap146	JN243898	KP795047	JN243878	Ref. 53, 54
Radiatula burmana (Blanford, 1869) **comb. nov.**	Myanmar: Irrawaddy River basin, Lake Indawgyi	WI	RMBH: biv_107_1	Hap062	KX865838	KX865609	KX865710	Ref. 9
Radiatula burmana (Blanford, 1869) **comb. nov.**	Myanmar: Irrawaddy River basin, Lake Indawgyi	WI	RMBH: biv_107_2	Hap063	KX865839	KX865610	KX865711	Ref. 9

Taxa	Locality	Range Code	Specimen Voucher*	Haplotype Code	COI	16S rRNA	28S rDNA	Reference
Radiatula burmana (Blanford, 1869) **comb. nov.**	Myanmar: Irrawaddy River basin, Lake Indawgyi	WI	RMBH: biv_107_3	Hap064	KX865840	KX865611	KX865712	Ref. 9
Radiatula burmana (Blanford, 1869) **comb. nov.**	Myanmar: Irrawaddy River basin, Lake Indawgyi	WI	RMBH: biv_106_1	Hap101	KX865841	KX865612	KX865713	Ref. 9
Radiatula burmana (Blanford, 1869) **comb. nov.**	Myanmar: Irrawaddy River basin, Lake Indawgyi	WI	RMBH: biv_106_3	Hap101	KX865842	KX865614	KX865714	Ref. 9
Radiatula burmana (Blanford, 1869) **comb. nov.**	Myanmar: Irrawaddy River basin, Lake Indawgyi	WI	RMBH: biv_106_2	Hap102	KX865843	KX865613	KX865715	Ref. 9
Radiatula aff. *humilis* (Lea, 1856) sp.1	Thailand: Mekong River basin, Chi River	PM	RMBH: biv_126_1	Hap058	KX865844	KX865615	KX865716	Ref. 9
Radiatula aff. *humilis* (Lea, 1856) sp.1	Thailand: Mekong River basin, Chi River	PM	RMBH: biv_126_2	Hap059	KX865845	KX865616	KX865717	Ref. 9
Radiatula aff. *humilis* (Lea, 1856) sp.1	Thailand: Mekong River basin, Chi River	PM	RMBH: biv_126_3	Hap060	KX865846	KX865617	KX865718	Ref. 9
Radiatula aff. *humilis* (Lea, 1856) sp.1	Thailand: Mekong River basin, Chi River	PM	RMBH: biv_129 _1	Hap092	KX865847	KX865618	KX865719	Ref. 9
Radiatula aff. *humilis* (Lea, 1856) sp.1	Thailand: Mekong River basin, Chi River	PM	RMBH: biv_129 _3	Hap092	KX865848	KX865620	KX865720	Ref. 9
Radiatula aff. *humilis* (Lea, 1856) sp.1	Thailand: Mekong River basin, Chi River	PM	RMBH: biv_129 _2	Hap093	KX865849	KX865619	KX865721	Ref. 9
Radiatula aff. *humilis* (Lea, 1856) sp.2	Thailand: Mekong River basin, Chi River	PM	RMBH: biv_124_1	Hap079	KX865850	KX865621	KX865722	Ref. 9
Radiatula aff. *humilis* (Lea, 1856) sp.2	Thailand: Mekong River basin, Chi River	PM	RMBH: biv_124_2	Hap080	KX865851	KX865622	KX865723	Ref. 9
Radiatula aff. *humilis* (Lea, 1856) sp.3	Cambodia: Mekong River basin	PM	UMMZ:304646	Hap150	KP795023	KP795048	KP795006	Ref. 54
Oxynaia sp. 'Tavoy'	Myanmar: Tavoy River	WI	RMBH: biv_147_3	Hap132	KX865852	KX865623	KX865724	Ref. 9
Oxynaia sp. 'Tavoy'	Myanmar: Tavoy River	WI	RMBH: biv_147_10	Hap132	KX865853	KX865624	KX865725	Ref. 9
Oxynaia sp. 'Tavoy'	Myanmar: Tavoy River	WI	RMBH: biv_147_18	Hap132	KX865854	KX865625	KX865726	Ref. 9
Oxynaia sp. 'Tavoy'	Myanmar: Tavoy River	WI	RMBH: biv_148_4	Hap132	KX865855	KX865626	KX865727	Ref. 9
Oxynaia sp. 'Tavoy'	Myanmar: Tavoy River	WI	RMBH: biv_148_7	Hap133	KX865856	KX865627	KX865728	Ref. 9
Oxynaia sp. 'Tavoy'	Myanmar: Tavoy River	WI	RMBH: biv_148_15	Hap134	KX865857	KX865628	KX865729	Ref. 9
Oxynaia aff. *pugio* (Benson, 1862) sp.1	Myanmar: Irrawaddy River basin, Chindwin River: Paukin Lake	WI	UA:20739.1/UMMZ : 304644	Hap151	JN243899	KP795046	JN243879	Ref. 53, 54
Oxynaia pugio (Benson, 1862)	Myanmar: Irrawaddy River basin, Lake Nant Phar	WI	RMBH: biv_258_1	Hap319	MF352261	MF352325	MF352386	Present study
Oxynaia pugio (Benson, 1862)	Myanmar: Irrawaddy River basin, Lake Nant Phar	WI	RMBH: biv_258_2	Hap320	MF352262	MF352326	MF352389	Present study
Oxynaia pugio (Benson, 1862)	Myanmar: Irrawaddy River basin, Lake Myaung	WI	RMBH: biv_268_1	Hap319	MF352285	n/a	n/a	Present study
Oxynaia pugio (Benson, 1862)	Myanmar: Irrawaddy River basin, Lake Myaung	WI	RMBH: biv_268_2	Hap319	MF352286	MF352346	MF352403	Present study
Oxynaia pugio (Benson, 1862)	Myanmar: Irrawaddy River basin, Lake Myaung	WI	RMBH: biv_268_4	Hap319	MF352287	MF352347	MF352404	Present study

Taxa	Locality	Range Code	Specimen Voucher*	Haplotype Code	COI	16S rRNA	28S rDNA	Reference
Oxynaia sp. "Taungoo"	Myanmar: Sittaung River basin, Myit Kyi Pauk stream	WI	RMBH: biv_251_1	Hap308	MF352242	MF352312	MF352370	Present study
Oxynaia sp. "Taungoo"	Myanmar: Sittaung River basin, Myit Kyi Pauk stream	WI	RMBH: biv_251_2	Hap308	MF352243	MF352313	MF352371	Present study
Oxynaia sp. "Taungoo"	Myanmar: Sittaung River basin, Myit Kyi Pauk stream	WI	RMBH: biv_251_3	Hap308	MF352244	MF352314	MF352372	Present study
PSEUDODONTINAE Frierson, 1927								
Pseudodontini Frierson, 1927								
Pseudodon avae (Theobald, 1873)	Myanmar: Irrawaddy River basin, a tributary of Lake Indawgyi	WI	RMBH: biv_110_5	Hap089	KX865858	KX865629	KX865730	Ref. 9
Pseudodon avae (Theobald, 1873)	Myanmar: Irrawaddy River basin, a tributary of Lake Indawgyi	WI	RMBH: biv_110_10	Hap089	KX865859	KX865630	KX865731	Ref. 9
Pseudodon avae (Theobald, 1873)	Myanmar: Irrawaddy River basin, a tributary of Lake Indawgyi	WI	RMBH: biv_110_11	Hap091	KX865860	KX865631	KX865732	Ref. 9
Pseudodon bogani **sp. nov.**	Myanmar: Sittaung River basin, Kanni River	WI	RMBH: biv_241_4	Hap292	MF352216	MF352290	MF352348	Present study
Pseudodon bogani **sp. nov.**	Myanmar: Sittaung River basin, Kanni River	WI	RMBH: biv_241_5	Hap293	MF352217	MF352291	MF352349	Present study
Pseudodon bogani **sp. nov.**	Myanmar: Sittaung River basin, Kanni River	WI	RMBH: biv_241_8	Hap294	MF352218	MF352292	MF352350	Present study
Pseudodon manueli **sp. nov.**	Myanmar: Sittaung River basin, Pyowne River	WI	RMBH: biv_246_1	Hap301	MF352228	MF352300	MF352358	Present study
Pseudodon manueli **sp. nov.**	Myanmar: Sittaung River basin, Pyowne River	WI	RMBH: biv_246_3	Hap302	MF352229	MF352301	MF352359	Present study
Pseudodon manueli **sp. nov.**	Myanmar: Sittaung River basin, Pyowne River	WI	RMBH: biv_246_8	Hap301	MF352230	MF352302	MF352360	Present study
Pilsbryoconchini Bolotov, Vikhrev et Tumpeesuwan **tribe nov.**								
Bineurus aff. *mouhotii* (Lea, 1863) sp.1	Laos: Mekong River basin, Nam Long River	PM	RMBH: biv_182_2	Hap110	KX865876	KX865647	KX865747	Ref. 9
Bineurus aff. *mouhotii* (Lea, 1863) sp.1	Laos: Mekong River basin, Nam Pe River	PM	RMBH: biv_183_4	Hap110	KX865877	KX865648	KX865748	Ref. 9
Bineurus aff. *mouhotii* (Lea, 1863) sp.1	Laos: Mekong River basin, Nam Long River	PM	RMBH: biv_182_19	Hap113	KX865878	KX865649	KX865749	Ref. 9
Bineurus aff. *mouhotii* (Lea, 1863) sp.1	Laos: Mekong River basin, a tributary of Nam Fa River near Vieng Phou Kha	PM	RMBH: biv_201_2	Hap187	KY561623	KY561641	KY561655	Ref. 9
Bineurus aff. *mouhotii* (Lea, 1863) sp.1	Laos: Mekong River basin, a tributary of Nam Fa River near Vieng Phou Kha	PM	RMBH: biv_201_3	Hap187	KY561624	KY561642	KY561656	Ref. 9
Bineurus aff. *mouhotii* (Lea, 1863) sp.1	Laos: Mekong River basin, a tributary of Nam Fa River near Vieng Phou Kha	PM	RMBH: biv_201_5	Hap187	KY561625	KY561643	KY561657	Ref. 9
Bineurus aff. *mouhotii* (Lea, 1863) sp.1	Laos: Mekong River basin, a tributary of Nam Fa River near Vieng Phou Kha	PM	RMBH: biv_202_1	Hap187	KY561626	KY561644	KY561658	Ref. 9
Bineurus aff. *mouhotii* (Lea, 1863) sp.2	Thailand: Mekong River basin, Loei River	PM	RMBH: biv_119_1	Hap045	KX865879	KX865650	KX865750	Ref. 9

Taxa	Locality	Range Code	Specimen Voucher*	Haplotype Code	COI	16S rRNA	28S rDNA	Reference
Bineurus aff. *mouhotii* (Lea, 1863) sp.2	Thailand: Mekong River basin, Loei River	PM	RMBH: biv_119_2	Hap045	KX865880	KX865651	KX865751	Ref. 9
Bineurus aff. *mouhotii* (Lea, 1863) sp.2	Thailand: Mekong River basin, Loei River	PM	RMBH: biv_119_3	Hap047	KX865881	KX865652	KX865752	Ref. 9
Bineurus aff. *mouhotii* (Lea, 1863) sp.2	Thailand: Mekong River basin, Loei River	PM	RMBH: biv_119_4	Hap047	KX865882	KX865653	KX865753	Ref. 9
Bineurus aff. *mouhotii* (Lea, 1863) sp.3	Laos: Mekong River basin	PM	UMMZ:304649	Hap177	KP795026	KP795051	KP795009	Ref. 54
Bineurus aff. *mouhotii* (Lea, 1863) sp.3	Laos: Mekong River basin	PM	NCSM84903	Hap202	KX822663	n/a	KX822619	Ref. 17
"*Bineurus*" *ellipticus* (Conrad, 1865)	Thailand: Mekong River basin, Phong River	PM	RMBH: biv_120_4	Hap048	KX865862	KX865633	KX865734	Ref. 9
"*Bineurus*" *ellipticus* (Conrad, 1865)	Thailand: Mekong River basin, Phong River	PM	RMBH: biv_120_12	Hap049	KX865863	KX865634	KX865735	Ref. 9
"*Bineurus*" *ellipticus* (Conrad, 1865)	Thailand: Mekong River basin, Phong River	PM	RMBH: biv_120_15	Hap049	KX865864	KX865635	KX865736	Ref. 9
"*Bineurus*" *ellipticus* (Conrad, 1865)	Thailand: Mekong River basin, Phong River	PM	RMBH: biv_120_3	Hap052	KX865865	KX865636	KX865737	Ref. 9
"*Bineurus*" *ellipticus* (Conrad, 1865)	Thailand: Mekong River basin, Phong River	PM	RMBH: biv_120_8	Hap053	KX865866	KX865637	KX865738	Ref. 9
"*Bineurus*" *ellipticus* (Conrad, 1865)	Thailand: Mekong River basin, Phong River	PM	RMBH: biv_120_14	Hap054	KX865867	KX865638	KX865739	Ref. 9
"*Bineurus*" *ellipticus* (Conrad, 1865)	Thailand: Mekong River basin, Phong River	PM	RMBH: biv_120_7	Hap065	KX865868	KX865639	KX865740	Ref. 9
"*Bineurus*" *ellipticus* (Conrad, 1865)	Thailand: Mekong River basin, Phong River	PM	RMBH: biv_120_11	Hap066	KX865869	KX865640	KX865741	Ref. 9
"*Bineurus*" *ellipticus* (Conrad, 1865)	Thailand: Mekong River basin, Phong River	PM	RMBH: biv_120_13	Hap067	KX865870	KX865641	KX865742	Ref. 9
"*Bineurus*" *ellipticus* (Conrad, 1865)	Thailand: Mekong River basin, Phong River	PM	RMBH: biv_205_4	Hap191	KY561622	KY561640	KY561654	Ref. 9
"*Bineurus*" *cumingii* (Lea, 1850) **comb. nov.**	Laos: Mekong River basin	PM	UMMZ:304648	Hap176	KP795025	KP795050	KP795008	Ref. 54
"*Bineurus*" *cumingii* (Lea, 1850) **comb. nov.**	Laos: Mekong River basin	PM	NCSM84884	Hap201	KX822662	n/a	KX822618	Ref. 17
"*Bineurus*" sp.1	Malaysia	PM	X115	Hap214	KX051295	n/a	n/a	Ref. 21
"*Bineurus*" sp.1	Malaysia	PM	X79	Hap216	KX051292	n/a	n/a	Ref. 21
"*Bineurus*" sp.1	Malaysia	PM	X89	Hap217	KX051293	n/a	n/a	Ref. 21
"*Monodontina*" aff. "*inoscularis*" sp.1	SE Asia	PM	H418.f	Hap181	DQ206793	n/a	n/a	GenBank
"*Monodontina*" aff. "*inoscularis*" sp.2	Cambodia: Mekong River basin	PM	UMMZ:304349/UMMZ:MC:304349	Hap178	KP795027	KF011261	KP795010	Ref. 54
Monodontina cambodjensis (Petit de la Saussaye, 1865) **comb. res.**	Cambodia: Mekong River basin, Tonle Sap River: Pursat River	PM	UMMZ:304350/UMMZ:MC:304350	Hap179	KP795028	KF011262	KP795011	Ref. 54
Monodontina cambodjensis (Petit de la Saussaye, 1865) **comb. res.**	Thailand	PM	n/a	Hap200	KX822660	n/a	KX822616	Ref. 17
Monodontina cambodjensis (Petit de la Saussaye, 1865) **comb. res.**	Malaysia	PM	X154	Hap210	KX051299	n/a	n/a	Ref. 21

Taxa	Locality	Range Code	Specimen Voucher*	Haplotype Code	COI	16S rRNA	28S rDNA	Reference
Monodontina cambodjensis (Petit de la Saussaye, 1865) **comb. res.**	Malaysia	PM	BIV1658	Hap211	KX051298	n/a	n/a	Ref. 21
Monodontina cambodjensis (Petit de la Saussaye, 1865) **comb. res.**	Malaysia	PM	X198	Hap212	KX051297	n/a	n/a	Ref. 21
Monodontina vondembuschiana (Lea, 1840) **comb. res.**	Malaysia	PM	BIV1693	Hap204	KX051311	n/a	n/a	Ref. 21
Monodontina vondembuschiana (Lea, 1840) **comb. res.**	Malaysia	PM	X38	Hap204	KX051308	n/a	n/a	Ref. 21
Monodontina vondembuschiana (Lea, 1840) **comb. res.**	Malaysia	PM	X39	Hap204	KX051307	n/a	n/a	Ref. 21
Monodontina vondembuschiana (Lea, 1840) **comb. res.**	Malaysia	PM	X001	Hap204	KX051305	n/a	n/a	Ref. 21
Monodontina vondembuschiana (Lea, 1840) **comb. res.**	Malaysia	PM	BIV1669	Hap204	KX051302	n/a	n/a	Ref. 21
Monodontina vondembuschiana (Lea, 1840) **comb. res.**	Malaysia	PM	BIV1692	Hap204	KX051300	n/a	n/a	Ref. 21
Monodontina vondembuschiana (Lea, 1840) **comb. res.**	Malaysia	PM	X157	Hap205	KX051306	n/a	n/a	Ref. 21
Monodontina vondembuschiana (Lea, 1840) **comb. res.**	Malaysia	PM	BIV1657	Hap205	KX051304	n/a	n/a	Ref. 21
Monodontina vondembuschiana (Lea, 1840) **comb. res.**	Malaysia	PM	BIV1670	Hap205	KX051301	n/a	n/a	Ref. 21
Monodontina vondembuschiana (Lea, 1840) **comb. res.**	Malaysia	PM	BIV1822	Hap207	KX051310	n/a	n/a	Ref. 21
Monodontina vondembuschiana (Lea, 1840) **comb. res.**	Malaysia	PM	X32	Hap208	KX051309	n/a	n/a	Ref. 21
Monodontina vondembuschiana (Lea, 1840) **comb. res.**	Malaysia	PM	BIV1806	Hap209	KX051303	n/a	n/a	Ref. 21
Monodontina vondembuschiana (Lea, 1840) **comb. res.**	Malaysia	PM	BIV1721	Hap213	KX051296	n/a	n/a	Ref. 21
Monodontina aff. *vondembuschiana* (Lea, 1840) sp.1	Thailand: Mekong River basin, Phong River	PM	RMBH: biv_122	Hap109	KX865861	KX865632	KX865733	Ref. 9
Monodontina aff. *vondembuschiana* (Lea, 1840) sp.2	Laos: Mekong River basin	PM	UMMZ:304650	Hap180	KP795029	KP795052	AF400694	Ref. 54, 86
Pilsbryoconcha compressa (Martens, 1860)	Thailand: Mekong River basin, artificial pond near the Ban Nong-Bua village	PM	RMBH: biv_116_1	Hap055	KX865872	KX865643	KX865744	Ref. 9
Pilsbryoconcha compressa (Martens, 1860)	Thailand: Mekong River basin, artificial pond near the Ban Nong-Bua village	PM	RMBH: biv_116_2	Hap056	KX865873	KX865644	KX865745	Ref. 9
Pilsbryoconcha compressa (Martens, 1860)	Thailand: Mekong River basin, artificial pond near the Ban Nong-Bua village	PM	RMBH: biv_116_3	Hap057	KX865874	KX865645	n/a	Ref. 9
Pilsbryoconcha compressa (Martens, 1860)	Thailand: Mekong River basin, Huai Nam Khu Reservoir	PM	RMBH: biv_118	Hap107	KX865875	KX865646	KX865746	Ref. 9
Pilsbryoconcha compressa (Martens, 1860)	Malaysia	PM	BIV1607	hap055	KX051285	n/a	n/a	Ref. 21
Pilsbryoconcha compressa (Martens, 1860)	Thailand	PM	n/a	Hap198	KX822656	n/a	KX822613	Ref. 17
Pilsbryoconcha compressa (Martens, 1860)	Malaysia	PM	X257	Hap218	KX051283	n/a	n/a	Ref. 21

Taxa	Locality	Range Code	Specimen Voucher*	Haplotype Code	COI	16S rRNA	28S rDNA	Reference
Pilsbryoconcha compressa (Martens, 1860)	Malaysia	PM	X278	Hap219	KX051284	n/a	n/a	Ref. 21
Pilsbryoconcha aff. *compressa* (Martens, 1860) sp.1	Thailand: Mekong River basin, Chi River	PM	RMBH: biv_125_1	Hap086	KX865871	KX865642	KX865743	Ref. 9
Pilsbryoconcha exilis (Lea, 1838)	Malaysia	PM	X213	Hap206	KX051289	n/a	n/a	Ref. 21
Pilsbryoconcha exilis (Lea, 1838)	Malaysia	PM	X85	Hap206	KX051290	n/a	n/a	Ref. 21
Pilsbryoconcha exilis (Lea, 1838)	Malaysia	PM	X200	Hap206	KX051286	n/a	n/a	Ref. 21
Pilsbryoconcha exilis (Lea, 1838)	Malaysia	PM	X131	Hap206	KX051287	n/a	n/a	Ref. 21
Pilsbryoconcha exilis (Lea, 1838)	Malaysia	PM	X129	Hap206	KX051288	n/a	n/a	Ref. 21
Pilsbryoconcha aff. *exilis* (Lea, 1838) sp.1	Cambodia: Mekong River basin	PM	UMMZ:304647	Hap175	KP795024	KP795049	KP795007	Ref. 54
Pilsbryoconcha aff. *exilis* (Lea, 1838) sp.2	Malaysia	PM	X219	Hap220	KX051291	n/a	n/a	Ref. 21
RECTIDENTINAE Modell, 1942								
Contradentini Modell, 1942								
Contradens contradens (Lea, 1838)	West Malaysia: Pahang River	PM	ANSP:389059	Hap184	DQ191411	n/a	AF400692	Ref. 86, 87
Contradens contradens (Lea, 1838)	Malaysia	PM	X076	Hap215	KX051294	n/a	n/a	Ref. 21
Contradens contradens (Lea, 1838)	Malaysia	PM	BIV1787	Hap229	KX051270	n/a	n/a	Ref. 21
Contradens contradens (Lea, 1838)	Malaysia	PM	BIV1789	Hap229	KX051269	n/a	n/a	Ref. 21
Contradens contradens (Lea, 1838)	Malaysia	PM	BIV1765	Hap229	KX051265	n/a	n/a	Ref. 21
Contradens contradens (Lea, 1838)	Malaysia	PM	BIV1638	Hap229	KX051259	n/a	n/a	Ref. 21
Contradens contradens (Lea, 1838)	Malaysia	PM	X054	Hap229	KX051251	n/a	n/a	Ref. 21
Contradens contradens (Lea, 1838)	Malaysia	PM	X060	Hap229	KX051250	n/a	n/a	Ref. 21
Contradens contradens (Lea, 1838)	Malaysia	PM	X072	Hap229	KX051247	n/a	n/a	Ref. 21
Contradens contradens (Lea, 1838)	Malaysia	PM	RMBH: biv_211_2	Hap229	MF352289	n/a	MF352406	Present study
Contradens contradens (Lea, 1838)	Malaysia	PM	BIV1767	Hap230	KX051253	n/a	n/a	Ref. 21
Contradens contradens (Lea, 1838)	Malaysia	PM	BIV1695	Hap230	KX051249	n/a	n/a	Ref. 21
Contradens contradens (Lea, 1838)	Malaysia	PM	X132	Hap231	KX051257	n/a	n/a	Ref. 21
Contradens contradens (Lea, 1838)	Malaysia	PM	X100	Hap231	KX051246	n/a	n/a	Ref. 21
Contradens contradens (Lea, 1838)	Malaysia	PM	X208	Hap232	KX051258	n/a	n/a	Ref. 21
Contradens contradens (Lea, 1838)	Malaysia	PM	BIV1764	Hap232	KX051254	n/a	n/a	Ref. 21

Taxa	Locality	Range Code	Specimen Voucher*	Haplotype Code	COI	16S rRNA	28S rDNA	Reference
Contradens contradens (Lea, 1838)	Malaysia	PM	BIV1777	Hap233	KX051262	n/a	n/a	Ref. 21
Contradens contradens (Lea, 1838)	Malaysia	PM	BIV1748	Hap233	KX051244	n/a	n/a	Ref. 21
Contradens contradens (Lea, 1838)	Malaysia	PM	BIV1709	Hap234	KX051267	n/a	n/a	Ref. 21
Contradens contradens (Lea, 1838)	Malaysia	PM	BIV1757	Hap234	KX051243	n/a	n/a	Ref. 21
Contradens contradens (Lea, 1838)	Malaysia	PM	BIV1710	Hap235	KX051268	n/a	n/a	Ref. 21
Contradens contradens (Lea, 1838)	Malaysia	PM	BIV1839	Hap236	KX051266	n/a	n/a	Ref. 21
Contradens contradens (Lea, 1838)	Malaysia	PM	BIV1639	Hap237	KX051264	n/a	n/a	Ref. 21
Contradens contradens (Lea, 1838)	Malaysia	PM	X231	Hap238	KX051261	n/a	n/a	Ref. 21
Contradens contradens (Lea, 1838)	Malaysia	PM	BIV1714	Hap239	KX051256	n/a	n/a	Ref. 21
Contradens contradens (Lea, 1838)	Malaysia	PM	BIV1758	Hap240	KX051255	n/a	n/a	Ref. 21
Contradens contradens (Lea, 1838)	Malaysia	PM	X43	Hap241	KX051252	n/a	n/a	Ref. 21
Contradens aff. *contradens* (Lea, 1838) sp.1	Cambodia: Mekong River basin	PM	UMMZ:304652	Hap183	KP795034	KP795054	KP795016	Ref. 54
Contradens eximius (Lea, 1856) **comb. nov.**	Thailand: Mekong River basin, Chi River	PM	RMBH: biv_127 _1	Hap095	KX865936	n/a	KX865807	Ref. 9
Contradens eximius (Lea, 1856) **comb. nov.**	Thailand: Mekong River basin, Chi River	PM	RMBH: biv_127 _2	Hap096	KX865937	n/a	KX865808	Ref. 9
Contradens eximius (Lea, 1856) **comb. nov.**	Thailand: Mekong River basin, Chi River	PM	RMBH: biv_127 _3	Hap095	KX865938	KX865686	KX865809	Ref. 9
Contradens eximius (Lea, 1856) **comb. nov.**	Thailand: Mekong River basin, Phong River	PM	RMBH: biv_121 _1	Hap103	KX865939	KX865687	KX865810	Ref. 9
Contradens eximius (Lea, 1856) **comb. nov.**	Thailand: Mekong River basin, Phong River	PM	RMBH: biv_121_2	Hap095	KX865940	KX865688	KX865811	Ref. 9
Contradens eximius (Lea, 1856) **comb. nov.**	Thailand: Mekong River basin, Phong River	PM	RMBH: biv_121_3	Hap096	KX865941	KX865689	KX865812	Ref. 9
Contradens sp.'Nam Long'	Thailand: Mekong River basin, Loei River	PM	RMBH: biv_119_5	Hap061	KX865928	KX865682	KX865799	Ref. 9
Contradens sp.'Nam Long'	Thailand: Mekong River basin, Loei River	PM	RMBH: biv_119_6	Hap061	KX865929	KX865683	KX865800	Ref. 9
Contradens sp.'Nam Long'	Laos: Mekong River basin, Nam Long River	PM	RMBH: biv_182_3	Hap111	KX865930	KX865684	KX865801	Ref. 9
Contradens sp.'Nam Long'	Laos: Mekong River basin, Nam Long River	PM	RMBH: biv_182_12	Hap111	KX865931	KX865685	KX865802	Ref. 9
Contradens sp.'Nam Long'	Laos: Mekong River basin, Nam Long River	PM	RMBH: biv_182_10	Hap111	KX865932	n/a	KX865803	Ref. 9
Contradens sp.'Nam Long'	Laos: Mekong River basin, Nam Pe River	PM	RMBH: biv_184_1	Hap061	KX865933	n/a	KX865804	Ref. 9
Contradens sp.'Nam Long'	Laos: Mekong River basin, Nam Pe River	PM	RMBH: biv_184_3	Hap061	KX865934	n/a	KX865805	Ref. 9
Contradens sp.'Nam Long'	Laos: Mekong River basin, Nam Pe River	PM	RMBH: biv_185_2	Hap061	KX865935	n/a	KX865806	Ref. 9

Taxa	Locality	Range Code	Specimen Voucher*	Haplotype Code	COI	16S rRNA	28S rDNA	Reference
Contradens sp.'Vieng Phou Kha'	Laos: Mekong River basin, a tributary of Nam Fa River near Vieng Phou Kha	PM	biv202_2	Hap188	KY561630	KY561645	KY561662	Ref. 9
Contradens sp.'Vieng Phou Kha'	Laos: Mekong River basin, a tributary of Nam Fa River near Vieng Phou Kha	PM	biv203_5	Hap188	KY561631	KY561646	KY561663	Ref. 9
Contradens sp.'Vieng Phou Kha'	Laos: Mekong River basin, a tributary of Nam Fa River near Vieng Phou Kha	PM	biv203_4	Hap189	KY561632	KY561647	KY561664	Ref. 9
Physunio modelli Brandt, 1974	Thailand: Mekong River basin, Chi River	PM	RMBH: biv_125_2	Hap087	KX865883	KX865654	KX865754	Ref. 9
Physunio modelli Brandt, 1974	Thailand: Mekong River basin, Chi River	PM	RMBH: biv_125_3	Hap088	KX865884	KX865655	KX865755	Ref. 9
Physunio modelli Brandt, 1974	Thailand: Mekong River basin, Chi River	PM	RMBH: biv_131_1	Hap087	KX865885	n/a	KX865756	Ref. 9
Physunio modelli Brandt, 1974	Thailand: Mekong River basin, Chi River	PM	RMBH: biv_131_2	Hap087	KX865886	n/a	KX865757	Ref. 9
Physunio modelli Brandt, 1974	Thailand: Mekong River basin, Chi River	PM	RMBH: biv_131_3	Hap087	KX865887	n/a	KX865758	Ref. 9
Physunio modelli Brandt, 1974	Thailand: Mekong River basin, Chi River	PM	RMBH: biv_131	Hap108	KX865888	n/a	KX865759	Ref. 9
Physunio modelli Brandt, 1974	Thailand: Mekong River basin, Phong River	PM	RMBH: biv_205_1	Hap190	KY561627	n/a	KY561659	Ref. 9
Physunio modelli Brandt, 1974	Thailand: Mekong River basin, Phong River	PM	RMBH: biv_205_2	Hap087	KY561628	n/a	KY561660	Ref. 9
Physunio modelli Brandt, 1974	Thailand: Mekong River basin, Phong River	PM	RMBH: biv_205_3	Hap087	KY561629	n/a	KY561661	Ref. 9
Physunio modelli Brandt, 1974	Thailand	PM	n/a	Hap192	KX822655	n/a	n/a	Ref. 17
Physunio sp.'Contradens'	Cambodia: Mekong River basin	PM	UMMZ:304653	Hap185	KP795035	KP795055	KP795017	Ref. 54
Physunio sp.'Trapezoideus'	Laos: Mekong River basin, Nam Ou River	PM	UMMZ:304347/UMMZ:MC:304347	Hap186	KP795036	KF011265	KP795018	Ref. 54
Physunio superbus (Lea, 1843)	Malaysia	PM	X180	Hap221	KX051282	n/a	n/a	Ref. 21
Physunio superbus (Lea, 1843)	Malaysia	PM	BIV1699	Hap221	KX051280	n/a	n/a	Ref. 21
Physunio superbus (Lea, 1843)	Malaysia	PM	X236	Hap221	KX051277	n/a	n/a	Ref. 21
Physunio superbus (Lea, 1843)	Malaysia	PM	X239	Hap221	KX051276	n/a	n/a	Ref. 21
Physunio superbus (Lea, 1843)	Malaysia	PM	BIV1776	Hap222	KX051278	n/a	n/a	Ref. 21
Physunio superbus (Lea, 1843)	Malaysia	PM	BIV1698	Hap222	KX051281	n/a	n/a	Ref. 21
Physunio superbus (Lea, 1843)	Malaysia	PM	BIV1756	Hap222	KX051279	n/a	n/a	Ref. 21
Physunio superbus (Lea, 1843)	Malaysia	PM	X246	Hap223	KX051275	n/a	n/a	Ref. 21
Trapezoideus nesemanni **sp. nov.**	Myanmar: Sittaung River basin, Tauk Ue Kupt River	WI	RMBH: biv_144_14	Hap127	KX865906	KX865663	KX865777	Ref. 9
Trapezoideus nesemanni **sp. nov.**	Myanmar: Sittaung River basin, Tauk Ue Kupt River	WI	RMBH: biv_144_25	Hap127	KX865907	KX865664	KX865778	Ref. 9
Trapezoideus nesemanni **sp. nov.**	Myanmar: Sittaung River basin, Tauk	WI	RMBH: biv_144_19	Hap128	KX865908	KX865665	KX865779	Ref. 9

Taxa	Locality	Range Code	Specimen Voucher*	Haplotype Code	COI	16S rRNA	28S rDNA	Reference
	Ue Kupt River							
Trapezoideus nesemanni **sp. nov.**	Myanmar: Sittaung River basin, Tauk Ue Kupt River	WI	RMBH: biv_255_2	Hap314	MF352254	n/a	MF352379	Present study
Trapezoideus nesemanni **sp. nov.**	Myanmar: Sittaung River basin, Tauk Ue Kupt River	WI	RMBH: biv_255_3	Hap314	MF352255	n/a	MF352380	Present study
Trapezoideus nesemanni **sp. nov.**	Myanmar: Sittaung River basin, Tauk Ue Kupt River	WI	RMBH: biv_255_4	Hap314	MF352256	n/a	MF352381	Present study
Trapezoideus panhai **sp. nov.**	Myanmar: Sittaung River basin, Kyan Hone River	WI	RMBH: biv_138_4	Hap115	KX865909	KX865666	KX865780	Ref. 9
Trapezoideus panhai **sp. nov.**	Myanmar: Sittaung River basin, Kyan Hone River	WI	RMBH: biv_138_7	Hap115	KX865910	KX865667	KX865781	Ref. 9
Trapezoideus panhai **sp. nov.**	Myanmar: Sittaung River basin, Kyan Hone River	WI	RMBH: biv_155_4	Hap115	KX865911	KX865668	KX865782	Ref. 9
Trapezoideus panhai **sp. nov.**	Myanmar: Sittaung River basin, Kyan Hone River	WI	RMBH: biv_155_25	Hap115	KX865912	KX865669	KX865783	Ref. 9
Trapezoideus panhai **sp. nov.**	Myanmar: Sittaung River basin, Kyan Hone River	WI	RMBH: biv_138_12	Hap116	KX865913	KX865670	KX865784	Ref. 9
Trapezoideus panhai **sp. nov.**	Myanmar: Sittaung River basin, Kyan Hone River	WI	RMBH: biv_155_11	Hap116	KX865914	KX865671	KX865785	Ref. 9
Trapezoideus sp.'Salween'	Myanmar: Salween River basin, Lake Inle	WI	RMBH: biv_114_1	Hap050	KX865915	KX865672	KX865786	Ref. 9
Trapezoideus sp.'Salween'	Myanmar: Salween River basin, Lake Inle	WI	RMBH: biv_114_3	Hap050	KX865916	KX865673	KX865787	Ref. 9
Trapezoideus sp.'Salween'	Myanmar: Salween River basin, Snake Stream	WI	RMBH: biv_143_2	Hap050	KX865917	KX865674	KX865788	Ref. 9
Trapezoideus sp.'Salween'	Myanmar: Salween River basin, Lake Inle	WI	RMBH: biv_114_2	Hap051	KX865918	KX865675	KX865789	Ref. 9
Trapezoideus sp.'Salween'	Myanmar: Salween River basin, Lake Inle	WI	RMBH: biv_115_1	Hap050	KX865919	n/a	KX865790	Ref. 9
Trapezoideus sp.'Salween'	Myanmar: Salween River basin, Lake Inle	WI	RMBH: biv_115_3	Hap050	KX865920	n/a	KX865791	Ref. 9
Trapezoideus sp.'Salween'	Myanmar: Salween River basin, Lake Inle	WI	RMBH: biv_115_2	Hap082	KX865921	n/a	KX865792	Ref. 9
Trapezoideus sp.'Salween'	Myanmar: Salween River basin, Snake Stream	WI	RMBH: biv_139_7	Hap117	KX865922	KX865676	KX865793	Ref. 9
Trapezoideus sp.'Salween'	Myanmar: Salween River basin, Snake Stream	WI	RMBH: biv_139_15	Hap118	KX865923	KX865677	KX865794	Ref. 9
Trapezoideus sp.'Salween'	Myanmar: Salween River basin, Snake Stream	WI	RMBH: biv_139_18	Hap119	KX865924	KX865678	KX865795	Ref. 9
Trapezoideus sp.'Salween'	Myanmar: Salween River basin, Nam Pilu River	WI	RMBH: biv_140_22	Hap120	KX865925	KX865679	KX865796	Ref. 9
Trapezoideus sp.'Salween'	Myanmar: Salween River basin, Nam Pilu River	WI	RMBH: biv_140_24	Hap121	KX865926	KX865680	KX865797	Ref. 9
Trapezoideus sp.'Salween'	Myanmar: Salween River basin, Nam Pilu River	WI	RMBH: biv_140_25	Hap050	KX865927	KX865681	KX865798	Ref. 9
Trapezoideus subclathratus (Martens, 1899) **stat. res.** et **comb. nov.**	Myanmar: Irrawaddy River basin, Nanuinhka Chaung River	WI	RMBH: biv_111_2	Hap032	KX865889	KX865656	KX865760	Ref. 9

Taxa	Locality	Range Code	Specimen Voucher*	Haplotype Code	COI	16S rRNA	28S rDNA	Reference
Trapezoideus subclathratus (Martens, 1899) **stat. res.** et **comb. nov.**	Myanmar: Irrawaddy River basin, Nanuinhka Chaung River	WI	RMBH: biv_111_21	Hap032	KX865890	KX865657	KX865761	Ref. 9
Trapezoideus subclathratus (Martens, 1899) **stat. res.** et **comb. nov.**	Myanmar: Irrawaddy River basin, Nanuinhka Chaung River	WI	RMBH: biv_111_43	Hap034	KX865891	KX865658	KX865762	Ref. 9
Trapezoideus subclathratus (Martens, 1899) **stat. res.** et **comb. nov.**	Myanmar: Irrawaddy River basin, Mali Hka River basin, Pan Khai stream	WI	RMBH: biv_101_4	Hap036	KX865892	n/a	n/a	Ref. 9
Trapezoideus subclathratus (Martens, 1899) **stat. res.** et **comb. nov.**	Myanmar: Irrawaddy River basin, Mali Hka River basin, Nam Shu River	WI	RMBH: biv_105_24	Hap036	KX865893	n/a	n/a	Ref. 9
Trapezoideus subclathratus (Martens, 1899) **stat. res.** et **comb. nov.**	Myanmar: Irrawaddy River basin, Mali Hka River basin, Pan Khai stream	WI	RMBH: biv_101_5	Hap036	KX865894	KX865659	KX865763	Ref. 9
Trapezoideus subclathratus (Martens, 1899) **stat. res.** et **comb. nov.**	Myanmar: Irrawaddy River basin, Mali Hka River basin, Pan Khai stream	WI	RMBH: biv_101_6	Hap036	KX865895	n/a	KX865764	Ref. 9
Trapezoideus subclathratus (Martens, 1899) **stat. res.** et **comb. nov.**	Myanmar: Irrawaddy River basin, Mali Hka River basin, Mansakun River	WI	RMBH: biv_103_17	Hap036	KX865896	n/a	KX865765	Ref. 9
Trapezoideus subclathratus (Martens, 1899) **stat. res.** et **comb. nov.**	Myanmar: Irrawaddy River basin, Mali Hka River basin, Mansakun River	WI	RMBH: biv_103_18	Hap036	KX865897	n/a	KX865766	Ref. 9
Trapezoideus subclathratus (Martens, 1899) **stat. res.** et **comb. nov.**	Myanmar: Irrawaddy River basin, Mali Hka River basin, Mansakun River	WI	RMBH: biv_103_19	Hap036	KX865898	n/a	KX865767	Ref. 9
Trapezoideus subclathratus (Martens, 1899) **stat. res.** et **comb. nov.**	Myanmar: Irrawaddy River basin, Mali Hka River basin, Nam Balak River	WI	RMBH: biv_102_7	Hap039	KX865899	KX865660	KX865768	Ref. 9
Trapezoideus subclathratus (Martens, 1899) **stat. res.** et **comb. nov.**	Myanmar: Irrawaddy River basin, Mali Hka River basin, Nam Balak River	WI	RMBH: biv_102_11	Hap036	KX865900	KX865661	KX865769	Ref. 9
Trapezoideus subclathratus (Martens, 1899) **stat. res.** et **comb. nov.**	Myanmar: Irrawaddy River basin, Mali Hka River basin, Nam Balak River	WI	RMBH: biv_102_14	Hap036	KX865901	KX865662	KX865770	Ref. 9
Trapezoideus subclathratus (Martens, 1899) **stat. res.** et **comb. nov.**	Myanmar: Irrawaddy River basin, Mali Hka River basin, Nam Shu River	WI	RMBH: biv_105_31	Hap039	KX865902	n/a	KX865771	Ref. 9
Trapezoideus subclathratus (Martens, 1899) **stat. res.** et **comb. nov.**	Myanmar: Irrawaddy River basin, Mali Hka River basin, Nam Shu River	WI	RMBH: biv_105_32	Hap039	KX865903	n/a	KX865772	Ref. 9
Trapezoideus subclathratus (Martens, 1899) **stat. res.** et **comb. nov.**	Myanmar: Irrawaddy River basin, Mali Hka River basin, unnamed stream	WI	RMBH: biv_104_34	Hap039	KX865904	n/a	KX865773	Ref. 9
Trapezoideus subclathratus (Martens, 1899) **stat. res.** et **comb. nov.**	Myanmar: Irrawaddy River basin, Mali Hka River basin, unnamed stream	WI	RMBH: biv_104_35	Hap039	KX865905	n/a	KX865774	Ref. 9
Trapezoideus subclathratus (Martens, 1899) **stat. res.** et **comb. nov.**	Myanmar: Irrawaddy River basin, Mali Hka River basin, Pan Khai stream	WI	RMBH: biv_101_1	Hap035	n/a	n/a	KX865775	Ref. 9
Trapezoideus subclathratus (Martens, 1899) **stat. res.** et **comb. nov.**	Myanmar: Irrawaddy River basin, Mali Hka River basin, Nam Shu River	WI	RMBH: biv_105_22	Hap035	n/a	n/a	KX865776	Ref. 9
Rectidentini Modell, 1942								
Ensidens aff. *sagittarius* (Lea, 1856) sp.1	Cambodia: Mekong River basin	PM	UMMZ:304651	Hap182	KP795033	KP795053	KP795015	Ref. 54
Ensidens aff. *sagittarius* (Lea, 1856) sp.2	Thailand: Mekong River basin, artificial pond near the Ban Nong-Bua village	PM	RMBH: biv_117_1	Hap068	KX865942	KX865690	KX865813	Ref. 9
Ensidens aff. *sagittarius* (Lea, 1856) sp.2	Thailand: Mekong River basin, artificial pond near the Ban Nong-Bua village	PM	RMBH: biv_117_2	Hap068	KX865943	KX865691	KX865814	Ref. 9
Ensidens aff. *sagittarius* (Lea, 1856) sp.2	Thailand: Mekong River basin, artificial pond near the Ban Nong-Bua village	PM	RMBH: biv_117_3	Hap069	KX865944	KX865692	KX865815	Ref. 9

Taxa	Locality	Range Code	Specimen Voucher*	Haplotype Code	COI	16S rRNA	28S rDNA	Reference
Ensidens aff. *sagittarius* (Lea, 1856) sp.3	Thailand: Mekong River basin, Chi River	PM	RMBH: biv_128_1	Hap073	KX865945	KX865693	KX865816	Ref. 9
Ensidens aff. *sagittarius* (Lea, 1856) sp.3	Thailand: Mekong River basin, Chi River	PM	RMBH: biv_128_2	Hap074	KX865946	KX865694	KX865817	Ref. 9
Ensidens aff. *sagittarius* (Lea, 1856) sp.3	Thailand: Mekong River basin, Chi River	PM	RMBH: biv_128_3	Hap075	KX865947	n/a	KX865818	Ref. 9
Ensidens aff. *sagittarius* (Lea, 1856) sp.3	Thailand: Mekong River basin, Chi River	PM	RMBH: biv_123_1	Hap083	KX865948	n/a	KX865819	Ref. 9
Ensidens aff. *sagittarius* (Lea, 1856) sp.3	Thailand: Mekong River basin, Chi River	PM	RMBH: biv_123_2	Hap084	KX865949	KX865695	KX865820	Ref. 9
Ensidens aff. *sagittarius* (Lea, 1856) sp.3	Thailand: Mekong River basin, Chi River	PM	RMBH: biv_123_3	Hap085	KX865950	KX865696	KX865821	Ref. 9
Ensidens cf. *ingallsianus* (Lea, 1852)	Laos: Mekong River basin	PM	NCSM84889	Hap193	KX822641	n/a	KX822598	Ref. 17
Ensidens sp.1	Laos: Mekong River basin	PM	NCSM84902	Hap194	KX822642	n/a	KX822599	Ref. 17
Hyriopsis sp.2	Thailand: Mekong River basin, Chi River	PM	RMBH: biv_130_1	Hap076	KX865951	KX865697	KX865822	Ref. 9
Hyriopsis sp.2	Thailand: Mekong River basin, Chi River	PM	RMBH: biv_130_2	Hap077	KX865952	KX865698	KX865823	Ref. 9
Hyriopsis sp.2	Thailand: Mekong River basin, Chi River	PM	RMBH: biv_130_3	Hap078	KX865953	n/a	KX865824	Ref. 9
Hyriopsis sp.2	Thailand: Mekong River basin	PM	n/a	Hap195	KX822643	n/a	KX822600	Ref. 17
Hyriopsis sp.2	Thailand: Mekong River basin	PM	Hb5	Hap195	KX383941	n/a	n/a	Ref. 21
Hyriopsis sp.2	Thailand: Mekong River basin	PM	Hb4	Hap195	KX383940	n/a	n/a	Ref. 21
Hyriopsis sp.2	Thailand: Mekong River basin	PM	Hb3	Hap195	KX383939	n/a	n/a	Ref. 21
Hyriopsis sp.1	Thailand: Chao Phraya basin	PM	839512HbB	Hap226	KX383948	n/a	n/a	Ref. 21
Hyriopsis sp.1	Thailand: Chao Phraya basin	PM	839511HbA	Hap227	KX383947	n/a	n/a	Ref. 21
Hyriopsis bialata Simpson, 1900	Malaysia	PM	BIV1774	Hap224	KX051274	n/a	n/a	Ref. 21
Hyriopsis bialata Simpson, 1900	Malaysia	PM	BIV1775	Hap224	KX051273	n/a	n/a	Ref. 21
Hyriopsis myersiana (Lea, 1856)	Thailand	PM	n/a	Hap197	KX822645	n/a	KX822602	Ref. 17
Hyriopsis myersiana (Lea, 1856)	Thailand: Mekong River basin	PM	Biv40	Hap197	KX383943	n/a	n/a	Ref. 21
Hyriopsis myersiana (Lea, 1856)	Thailand: Mekong River basin	PM	COI17	Hap225	KX383949	n/a	n/a	Ref. 21
Hyriopsis desowitzi Brandt, 1974	Thailand	PM	n/a	Hap196	KX822644	n/a	KX822601	Ref. 17
Hyriopsis desowitzi Brandt, 1974	Thailand: Chao Phraya basin	PM	Hd11	Hap196	KX383945	n/a	n/a	Ref. 21
Hyriopsis desowitzi Brandt, 1974	Thailand: Chao Phraya basin	PM	Hd10	Hap196	KX383944	n/a	n/a	Ref. 21
Hyriopsis desowitzi Brandt, 1974	Thailand: Chao Phraya basin	PM	n/a	Hap228	KX383946	n/a	n/a	Ref. 21
Rectidens sumatrensis (Dunker, 1852)	Malaysia	PM	n/a	Hap203	KX822664	n/a	KX822620	Ref. 17

Taxa	Locality	Range Code	Specimen Voucher*	Haplotype Code	COI	16S rRNA	28S rDNA	Reference
Rectidens sumatrensis (Dunker, 1852)	Malaysia	PM	biv 211_1	Hap203	MF352288	n/a	MF352405	Present study
UNIONINAE Rafinesque, 1820								
Unio crassus Philipsson, 1788	France	EU	n/a	hap260	KC703878	n/a	KC703644	Ref. 88
Unio pictorum (Linnaeus, 1758)	Europe	EU	n/a	hap261	KC429109	n/a	KC429447	Ref. 89
Unio tumidus Philipsson, 1788	Ukraine	EU	n/a	hap262	KX822672	n/a	KX822630	Ref. 17
Aculamprotula tortuosa (Lea, 1865)	China	EA	n/a	hap263	KX822631	n/a	KX822586	Ref. 17
Cuneopsis heudei (Heude, 1874)	China	EA	n/a	hap264	KX822638	n/a	KX822595	Ref. 17
Cuneopsis pisciculus (Heude, 1874)	China	EA	n/a	hap265	KX822639	n/a	KX822596	Ref. 17
Cuneopsis rufescens (Heude, 1874)	China	EA	n/a	hap266	KX822640	n/a	KX822597	Ref. 17
Nodularia douglasiae (Griffith & Pidgeon, 1833)	China	EA	n/a	hap267	KX822653	n/a	KX822610	Ref. 17
Nodularia nuxpersicae Dunker, 1848	Vietnam	EA	NCSM84990	hap268	KX822654	n/a	KX822611	Ref. 17
Schistodesmus lampreyanus (Baird & Adams, 1867)	China	EA	n/a	hap269	KX822665	n/a	KX822621	Ref. 17
ANODONTINAE Rafinesque, 1820								
Alasmidonta marginata Say, 1818	USA	NA	UMMZ 265695	hap242	AF156502	n/a	AF400688	Ref. 86
Anodonta anatina (Linnaeus, 1758)	European Russia	EU	n/a	hap243	KX822632	n/a	KX822588	Ref. 17
Anodonta cygnea (Linnaeus, 1758)	Italy	EU	n/a	hap244	KX822633	n/a	KX822589	Ref. 17
Anodonta nuttalliana Lea, 1838	USA	NA	n/a	hap245	KX822634	n/a	KX822590	Ref. 17
Lasmigona compressa (Lea, 1829)	USA	NA	UMMZ 265696	hap246	AF156503	n/a	DQ191414	Ref. 87, 90
Pseudanodonta complanata (Rossmässler, 1835)	Ukraine	EU	n/a	hap247	KX822661	n/a	KX822617	Ref. 17
Pyganodon grandis (Say, 1829)	USA	NA	n/a	hap248	AF231734	n/a	AF305384	Ref. 90, 91
Simpsonaias ambigua (Say, 1825)	USA	NA	NCSM30607	hap249	KX822666	n/a	KX822622	Ref. 17
Strophitus undulatus (Say, 1817)	USA	NA	UMMZ 265693	hap250	AF156505	n/a	DQ191415	Ref. 87, 90
Anemina sp.	Siberia	EA	n/a	hap251	KY561633	KY561648	KY561665	Ref. 9
Cristaria plicata (Leach, 1814)	Vietnam	EA	n/a	hap252	KY561634	n/a	KY561666	Ref. 9
Cristaria sp.	China	EA	n/a	hap253	EU698940	n/a	n/a	GenBank
Pletholophus tenuis (Griffith & Pidgeon, 1833)	Vietnam	EA	NCSM84924	hap254	KX822658	n/a	KX822614	Ref. 17
Sinanodonta lucida (Heude, 1877)	China	EA	n/a	hap255	KX822667	n/a	KX822624	Ref. 17
Sinanodonta sp.	Vietnam	EA	n/a	hap256	KY561635	KY561649	KY561667	Ref. 9

Taxa	Locality	Range Code	Specimen Voucher*	Haplotype Code	COI	16S rRNA	28S rDNA	Reference
Lanceolaria gladiola (Heude, 1877)	China	EA	n/a	hap257	KX822648	n/a	KX822605	Ref. 17
Lanceolaria grayana (Lea, 1834)	China	EA	n/a	hap258	KX822649	n/a	KX822606	Ref. 17
Lanceolaria grayii (Griffith & Pidgeon, 1833)	Vietnam	EA	n/a	hap259	KX822650	n/a	KX822607	Ref. 17
GONIDEINAE Ortmann, 1916								
Chamberlainia hainesiana (Lea, 1856)	Thailand	PM	n/a	hap270	KX822635	n/a	KX822592	Ref. 17
Sinohyriopsis cumingii (Lea, 1852)	Vietnam	EA	n/a	hap271	KY561636	KY561650	KY561668	Ref. 9
Lamprotula caveata (Heude, 1877)	China	EA	n/a	hap272	KX822646	n/a	KX822603	Ref. 17
Lamprotula leaii (Griffith & Pidgeon, 1833)	Vietnam	EA	n/a	hap273	KY561637	KY561651	KY561669	Ref. 9
Potomida littoralis (Cuvier, 1798)	France	EU	n/a	hap274	JN243905	n/a	JN243883	Ref. 37
*Pronodularia japanen*sis (Lea, 1859)	Japan	EA	NCSM27183	hap275	KX822659	KU946322	KX822615	Ref. 17, 92
Gonidea angulata (Lea, 1838)	USA	NA	n/a	hap276	DQ272371	n/a	AF400691	Ref. 86, 93
Leguminaia wheatleyi (Lea, 1862)	Turkey	EU	n/a	hap277	KX822651	n/a	KX822608	Ref. 17
Leguminaia sp.	Turkey	EU	n/a	hap278	KY561638	KY561652	n/a	Ref. 9
Microcondylaea bonellii (A. Ferussac 1827)	Italy	EU	n/a	hap279	KX822652	n/a	KX822609	Ref. 17
Solenaia carinata (Heude, 1877)	China	EA	n/a	hap280	KX822669	n/a	KX822626	Ref. 17
Solenaia oleivora (Heude, 1877)	China	EA	n/a	hap281	KX822670	n/a	KX822627	Ref. 17
Solenaia sp.	Vietnam	EA	n/a	hap282	KY561639	KY561653	KY561670	Ref. 9
AMBLEMINAE Rafinesque, 1820								
Amblema plicata (Say, 1817)	USA	NA	n/a	hap283	U56841	n/a	AF305385	Ref. 86, 94
Actinonaias ligamentina (Lamarck, 1819)	USA	NA	n/a	hap284	AF156517	n/a	DQ191420	Ref. 87, 90
Lampsilis cardium Rafinesque, 1820	USA	NA	n/a	hap285	AF120653	n/a	AF305386	Ref. 86, 95
Villosa iris (Lea, 1829)	USA	NA	n/a	hap286	AF156524	n/a	DQ191422	Ref. 87, 90
Elliptio complanata (Lightfoot, 1786)	USA	NA	n/a	hap287	EU448173	n/a	JF899181	Ref. 17, 96
Elliptio dilatata (Rafinesque, 1820)	USA	NA	n/a	hap288	AF156507	n/a	AF400690	Ref. 86, 90
Pleurobema sintoxia (Rafinesque, 1820)	USA	NA	n/a	hap289	AF156509	n/a	DQ191418	Ref. 87, 90
Quadrula quadrula (Rafinesque, 1820)	USA	NA	n/a	hap290	AF156511	n/a	DQ191417	Ref. 87, 90
Quadrula verrucosa (Rafinesque, 1820)	USA	NA	n/a	hap291	DQ191413	n/a	DQ191416	Ref. 87

Taxa	Locality	Range Code	Specimen Voucher*	Haplotype Code	COI	16S rRNA	28S rDNA	Reference
Out-Group Taxa								
MARGARITIFERIDAE Haas, 1940								
Margaritifera laosensis (Lea, 1863)	Laos: Mekong River basin, Nam Long River	not used	biv 186_1	Hap030	JX497731	KC845943	KT343741	Ref. 97
Margaritifera dahurica (Middendorff, 1850)	Far East of Russia: Amur River basin, Ilistaya River	not used	biv 92_6	Hap031	KJ161516	KJ943526	KT343747	Ref. 97
Margaritifera margaritifera (Linnaeus, 1758)	Northwestern Russia: Onega River basin, Somba River	not used	biv 618	Hap026	KX550089	KX550091	KX550093	Ref. 9
Margaritifera laevis (Haas, 1910)	Far East of Russia: Kurile Archipelago, Kunashir Island, Sennaya River	not used	biv d0036/22	Hap024	KJ161500	KJ943523	KT343742	Ref. 97
Margaritifera middendorffi (Rosén, 1926)	Far East of Russia: Kamchatka, Bolshaya River basin, Nachilova River	not used	biv d0099/6	Hap025	KJ161547	KJ943528	KT343745	Ref. 97
Margaritifera falcata (Gould, 1850)	USA: Idaho, Fremont Co., Buffalo River	not used	n/a	Hap027	AY579128	AY579085	n/a	Ref. 98
Margaritifera auricularia (Spengler, 1793)	Spain: Tarragona, Ebro River	not used	n/a	Hap010	AY579125	AY579083	n/a	Ref. 98
Margaritifera marocana (Pallary, 1918)	Morocco: Oum Er-Rbia River basin, El-Abid River	not used	n/a	Hap011	EU429679	EU429691	n/a	Ref. 99
Margaritifera monodonta (Say, 1829)	USA: Mississippi River basin	not used	n/a	Hap028	AY579131	AY579089	AF305382	Ref. 98, 101
Margaritifera marrianae Johnson, 1983	USA: Alabama River basin	not used	n/a	Hap029	HM849098	AY579086	n/a	Ref. 98, 100
IRIDINIDAE Swainson, 1840								
Aspatharia pfeifferiana (Bernardi, 1860)	Zambia: Chambeshi River	not used	n/a	Hap009	KC429107	KC429264	n/a	Ref. 101, 103
Chambardia wahlbergi (Krauss, 1848)	Zambia: Zambezi River	not used	n/a	Hap012	JN243886	KP184845	JN243864	Ref. 102
ETHERIIDAE Deshayes, 1832								
Etheria elliptica Lamarck, 1807	Zambia: Chambeshi River	not used	n/a	Hap014	KP184897	KP184847	KP184873	Ref. 102
MYCETOPODIDAE Gray, 1840		not used						
Anodontites elongata (Swainson, 1823)	Peru	not used	n/a	Hap013	KP184896	KP184846	KP184872	Ref. 102
HYRIIDAE Swainson, 1840								
Triplodon corrugatus (Lamarck, 1819)	Peru	not used	n/a	Hap015	JN243890	KP184851	JN243868	Ref. 102
Castalia ambigua Lamarck, 1819	Peru	not used	n/a	Hap016	JN243889	KP184848	JN243867	Ref. 102
Diplodon suavidicus (Lea, 1856)	Guyana	not used	n/a	Hap017	KP184898	KP184849	KP184874	Ref. 102
Microdontia anodontaeformis (Tapparone Canefri, 1883)	New Guinea	not used	n/a	Hap018	KP184909	KP184861	KP184885	Ref. 102
Alathyria jacksoni Iredale, 1934	Australia: New South Wales	not used	n/a	Hap019	KP184912	KP184864	KP184888	Ref. 102
Alathyria pertexta Iredale, 1934	Australia: Queensland	not used	n/a	Hap020	KP184910	KP184862	KP184886	Ref. 102

Taxa	Locality	Range Code	Specimen Voucher*	Haplotype Code	COI	16S rRNA	28S rDNA	Reference
Alathyria profuga (Gould, 1850)	Australia: New South Wales	not used	n/a	Hap021	KP184913	KP184865	KP184889	Ref. 102
Lortiella froggatti Iredale, 1934	Western Australia	not used	n/a	Hap022	AF231746	KP184867	KP184891	Ref. 102
Velesunio ambiguus (Philippi, 1847)	Australia: New South Wales	not used	n/a	Hap023	KP184915	KP184868	KP184892	Ref. 89, 102
TRIGONIIDAE Lamarck, 1819								
Neotrigonia margaritacea (Lamarck, 1804)	Tasmania and Australia	not used	n/a	Hap001	U56850	DQ280034	DQ279963	Ref. 94, 98, 103
Neotrigonia lamarckii (Gray, 1838)	Australia: Coral Sea, North Stradbroke Island, Queensland	not used	n/a	Hap002	KC429105	KC429262	KC429443	Ref. 89, 101

*All of our voucher specimens are deposited in RMBH, Russian Museum of Biodiversity Hotspots, the Federal Center for Integrated Arctic Research, Russian Academy of Sciences (Arkhangelsk, Russia).

Supplementary Table 2. List of sampling localities in Indo-China (new samples and data of Bolotov et al.[9]). *N* is the number of sequenced specimens, and *S* is the number of putative biological species at the locality

Country	Freshwater Drainage	Sampling Locality	Locality Code	Latitude (N)	Longitude (E)	Alt., m a.s.l.	Habitat	Meso-habitat	*N*	*S*
Myanmar	Sittaung River	Kyan Hone River	ST01	19.5059	96.8280	896	Mountain river	Run	6	1
Myanmar	Sittaung River	Tauk Ue Kupt River	ST02	19.3075	96.7219	426	Mountain river	Run	6	1
Myanmar	Sittaung River	Kanni River	ST03	19.0545	96.5131	60	Plain river	Run	3	1
Myanmar	Sittaung River	Pathi River	ST04	19.0278	96.5353	70	Plain river	Pool	6	2
Myanmar	Sittaung River	Reservoir at Yetho River	ST05	18.8457	96.3012	71	Reservoir	Pool	3	1
Myanmar	Sittaung River	Pyowne Stream	ST06	18.9694	96.5309	59	Plain stream	Run	3	1
Myanmar	Sittaung River	Fish breeding ponds near Taungoo	ST07	18.9593	96.4831	56	Reservoir	Pool	3	2
Myanmar	Sittaung River	Myit Kyi Pauk Stream	ST08	18.9613	96.4455	48	Plain stream	Run	6	2
Myanmar	Sittaung River	Near Taungoo	ST09	18.9840	96.4361	45	Plain river	Run	15	4
Myanmar	Irrawaddy River	A tributary of Lake Indawgyi	IR01	25.1209	96.2812	174	Plain stream	Pool	3	1
Myanmar	Irrawaddy River	Lake Indawgyi	IR02	25.1099	96.2925	170	Lake	Pool	12	3
Myanmar	Mali Hka River -> Irrawaddy River	Mansakun River	IR03	27.4909	97.3351	413	Mountain river	Run	3	1
Myanmar	Mali Hka River -> Irrawaddy River	Nam Balak River	IR04	27.4741	97.3493	418	Mountain river	Run	3	1
Myanmar	Mali Hka River -> Irrawaddy River	Nam Shu River	IR05	27.5482	97.3700	434	Mountain river	Run	4	1
Myanmar	Mali Hka River -> Irrawaddy River	Pan Khai Stream	IR06	27.4493	97.3432	209	Mountain stream	Pool	4	1
Myanmar	Mali Hka River -> Irrawaddy River	Unnamed stream	IR07	27.5475	97.3705	435	Mountain stream	Run	2	1
Myanmar	Irrawaddy River	Nanuinhka Chaung River	IR08	25.0815	96.2874	178	Plain stream	Run	3	1
Myanmar	Irrawaddy River	Nant Phar Lake	IR09	24.2972	97.2610	105	Lake	Pool	8	3
Myanmar	Irrawaddy River	Tar Pein River	IR10	24.3049	97.2514	105	Plain river	Run	3	1
Myanmar	Irrawaddy River	Shwe Kyi Lake	IR11	24.2927	97.2299	108	Lake	Pool	3	1

Country	Freshwater Drainage	Sampling Locality	Locality Code	Latitude (N)	Longitude (E)	Alt., m a.s.l.	Habitat	Meso-habitat	*N*	*S*
Myanmar	Irrawaddy River	Nant Sa Yi River	IR12	24.2196	97.2224	103	Plain river	Run	5	2
Myanmar	Irrawaddy River	Myaung Lake	IR13	24.2387	97.1658	104	Lake	Pool	11	4
Myanmar	Salween River	Lake Inle	SW01	20.4420	96.9036	887	Lake	Pool	9	2
Myanmar	Salween River	Lake Inle, a channel in Nuangshve	SW02	20.6632	96.9310	892	Lake	Pool	3	1
Myanmar	Salween River	Nam Pilu River	SW03	19.6746	97.1352	878	Plain river	Pool	6	2
Myanmar	Salween River	Snake Stream	SW04	19.7266	97.0992	878	Plain stream	Pool	5	2
Myanmar	Tavoy River	Tavoy River	TA01	14.5012	98.1557	18	Plain river	Run	13	3
Myanmar	Kaladan River	Unnamed puddle	KA01	21.0078	92.9831	14	Floodplain puddle	Pool	1	1
Laos	Nam Ou River -> Mekong River	Nam Long River	ME01	21.7700	102.1863	480	Mountain river	Run	5	2
Laos	Nam Ou River -> Mekong River	Nam Pe River	ME02	21.5905	102.0829	863	Mountain river	Run	4	2
Laos	Nam Fa River -> Mekong River	A tributary of Nam Fa River near Vieng Phou Kha	ME03	20.6820	101.0794	674	Mountain stream	Pool	7	2
Thailand	Mekong River	A pond near the Ban Nong-Bua village	ME04	17.4547	101.3958	650	Pond	Pool	6	2
Thailand	Mekong River	Chi River	ME05	16.2258	103.3007	145	Plain river	Run	27	7
Thailand	Mekong River	Huai Nam Khu Reservoir	ME06	17.1050	101.6193	288	Reservoir	Pool	1	1
Thailand	Mekong River	Loei River	ME07	17.0982	101.4814	531	Mountain river	Run	6	2
Thailand	Mekong River	Phong River	ME08	16.8616	101.9105	242	Plain river	Run	17	4
Malaysia	Perak River	Perak River	MA01	4.8848	100.9875	44	Plain river	Run	2	2

Supplementary Table 3. Habitats of the native Unionidae species from western Indo-China (our new data)

Genus	Species	Distribution	Habitat
Leoparreysia Vikhrev, Bolotov et Aksenova **gen. nov.**	*L. canefrii* Vikhrev, Bolotov et Kondakov **gen.** et **sp. nov.**	Sittaung	Pool sites with clay and silty-sand bottom in a plain river
	L. tavoyensis (Gould, 1843) **comb. nov.**	Tavoy	Pool and run sites with clay and gravel bottom in a plain river
Radiatula Simpson, 1900	*R. mouhoti* Vikhrev, Bolotov et Konopleva **sp. nov.**	Sittaung	Pool sites with clay and silty-sand bottom in a plain river
	R. myitkyinae (Prashad, 1930) **stat. res.** et **comb. nov.**	Irrawaddy	Volcanic rocky outcrop in Lake Indawgyi
	R. aff. *bonneaudii* (Eydoux, 1838) sp.1	Irrawaddy	Runs in a plain river
Oxynaia Haas, 1913	*O.* sp. 'Taungoo'	Sittaung	Pool and run sites with clay bottom in a plain stream
	O. sp. 'Tavoy'	Tavoy	Pool and run sites with clay and gravel bottom in a plain river
	O. pugio (Benson, 1862)	Irrawaddy	Lakes with clay and silty bottom
Indonaia Prashad, 1918 **stat. res.**	*I. andersoniana* (Nevill, 1877) **comb. res.**	Irrawaddy	Lakes with clay, rocky and silty bottom
Lamellidens Simpson, 1900	*L. brandti* Bolotov, Konopleva et Vikhrev **sp. nov.**	Sittaung	Plain rivers and lakes (eurytopic species)
	L. savadiensis (Nevill, 1877) **stat. res.**	Irrawaddy and Sittaung	Plain rivers and lakes (eurytopic species)
	L. generosus (Gould, 1847)	Salween	Plain rivers and lakes (eurytopic species)
	L. aff. *marginalis* (Lamarck, 1819) sp.3	Kaladan	A single specimen was sampled from a floodplain puddle with clay bottom; likely it is an eurytopic species like the majority of its congeners
Trapezidens Bolotov, Vikhrev et Konopleva **gen. nov.**	*T. obesa feae* Kondakov, Konopleva et Vikhrev **gen.** et **ssp. nov.**	Sittaung	Pool and run sites with clay bottom in a plain stream
	T. exolescens (Gould, 1843) **comb. nov.**	Tavoy	Pool and run sites with clay and gravel bottom in a plain river
Pseudodon Gould, 1844	*P. bogani* Bolotov, Kondakov et Konopleva **sp. nov.**	Sittaung	Runs with coarse-grained sand bottom in a plain river
	P. manueli Konopleva, Kondakov et Vikhrev **sp. nov.**	Sittaung	Runs with coarse-grained sand bottom in a plain stream
	P. avae (Theobald, 1873)	Irrawaddy	Pool sites with clay bottom in a plain stream
Trapezoideus Simpson 1900	*T. nesemanni* Konopleva, Vikhrev et Bolotov **sp. nov.**	Sittaung	Pool sites with clay and silty-sand bottom in a mountain stream
	T. panhai Konopleva, Bolotov et Kondakov **sp. nov.**	Sittaung	Rapids and runs with gravel and sandy bottom in a mountain river
	T. subclathratus (Martens, 1899) **stat. res.** et **comb. nov.**	Irrawaddy	From run sites with clay bottom in a plain stream to runs and boulder rapids in glacier feeding mountain tributaries of the Mali Hka River (Kachin State, the north of Myanmar). It seems to be a single Unionidae species inhabiting the upstream section of the Mali Hka River
	T. 'Salween'	Salween	Rivers and lakes (eurytopic species)

Supplementary Table 4. Primer sequences for PCR amplification and sequencing

Gene fragment	Primer's name	Direction	Sequence (5′-3′)	Reference
COI	LoboF1	Forward	kbtchacaaaycayaargayathgg	Ref. 104
	LoboR1	Reverse	taaacytcwggrtgwccraaraayca	
16S rRNA	16Sar	Forward	cgcctgtttatcaaaaacat	Ref. 105
	16sar-L-myt	Forward	cgactgtttaacaaaaacat	Ref. 106
	16sbr-H-myt	Reverse	ccgttctgaactcagctcatgt	
28S rDNA	C1	Forward	acccgctgaatttaagcat	Ref. 107
	D2	Reverse	tccgtgtttcaagacgg	

Supplementary Table 5. Alignment length prior to and after treatment for length variability in GBlocks v. 0.91b

Partition	Original length of alignment (bp)	Fraction selected by GBlocks (%)	Final length of alignment (bp)
COI	659	100	659
16S rRNA	531	84	445
28S rDNA	852	85	725

Supplementary Table 6. Probability (*p*-value) of phylogenetic conflict among sequence data sets from a partition-homogeneity test implemented in PAUP* v. 4.0a151

Sequence data set	16S	28S
COI	0.57	0.10
16S		0.98
COI+16S		0.45

Supplementary Table 7. Models of sequence evolution for each partition based on corrected Akaike Information Criterion (AICc) of MEGA6 that were applied within a Bayesian inference framework

Partition	Model	Gamma	Invariant
COI			
1st codon of COI	GTR+G	4.25	n/a
2nd codon of COI	GTR+G+I	1.11	0.48
3rd codon of COI	GTR+G	0.95	n/a
16S	GTR+G+I	0.30	0.29
28S	GTR+G	0.53	n/a

n/a – not available.

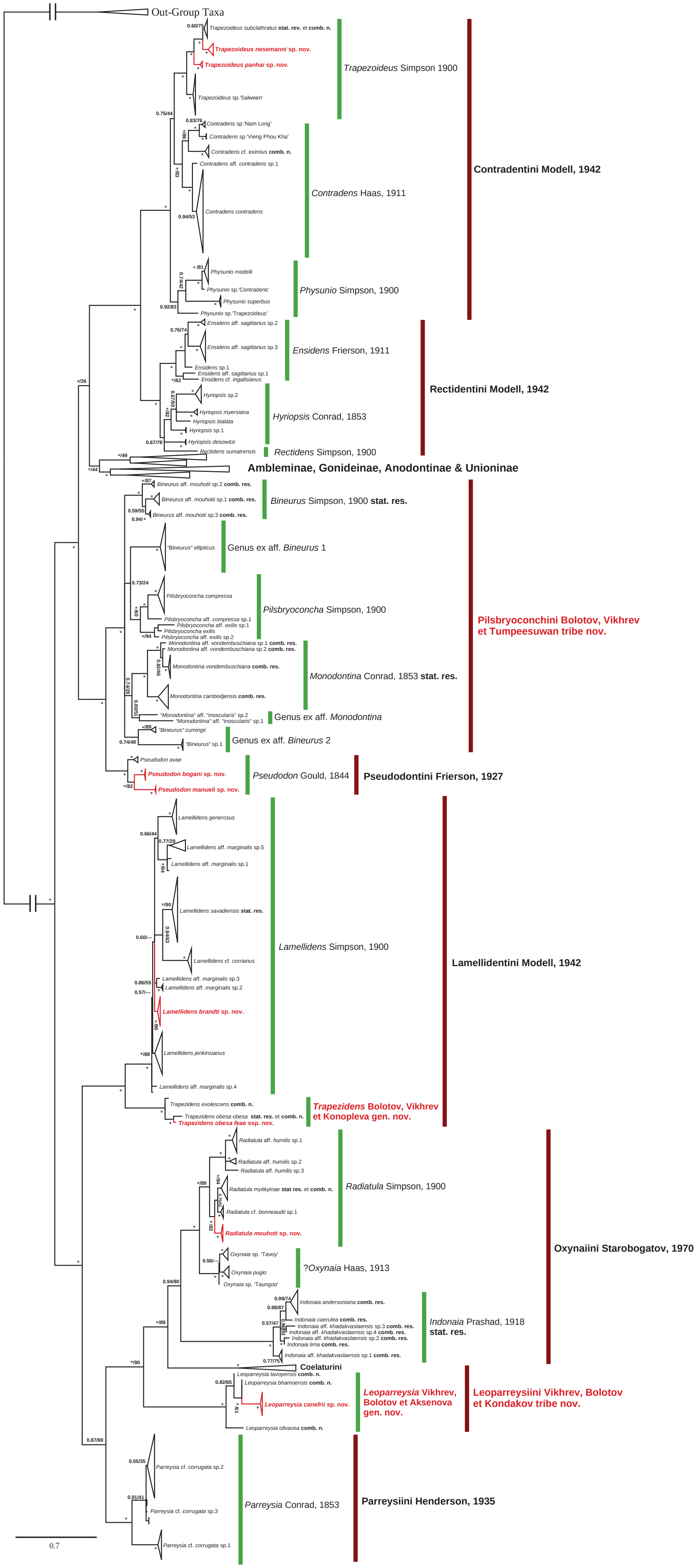

Supplementary Fig. 1. Majority rule consensus phylogenetic tree of the Unionidae recovered from Bayesian Inference analysis of the complete data set of mitochondrial and nuclear sequences (five partitions: three codons of COI + 16S rRNA + 28S rDNA). Numbers near branches are Bayesian posterior probabilities (BPP)/ML bootstrap support values («*» indicates values ≥0.95; «---» indicates topological difference). The red labels indicate new taxa described in the present study. The scale bar indicates the branch lengths. The nodes representing an out-group and taxa that were not discussed in this study are collapsed.

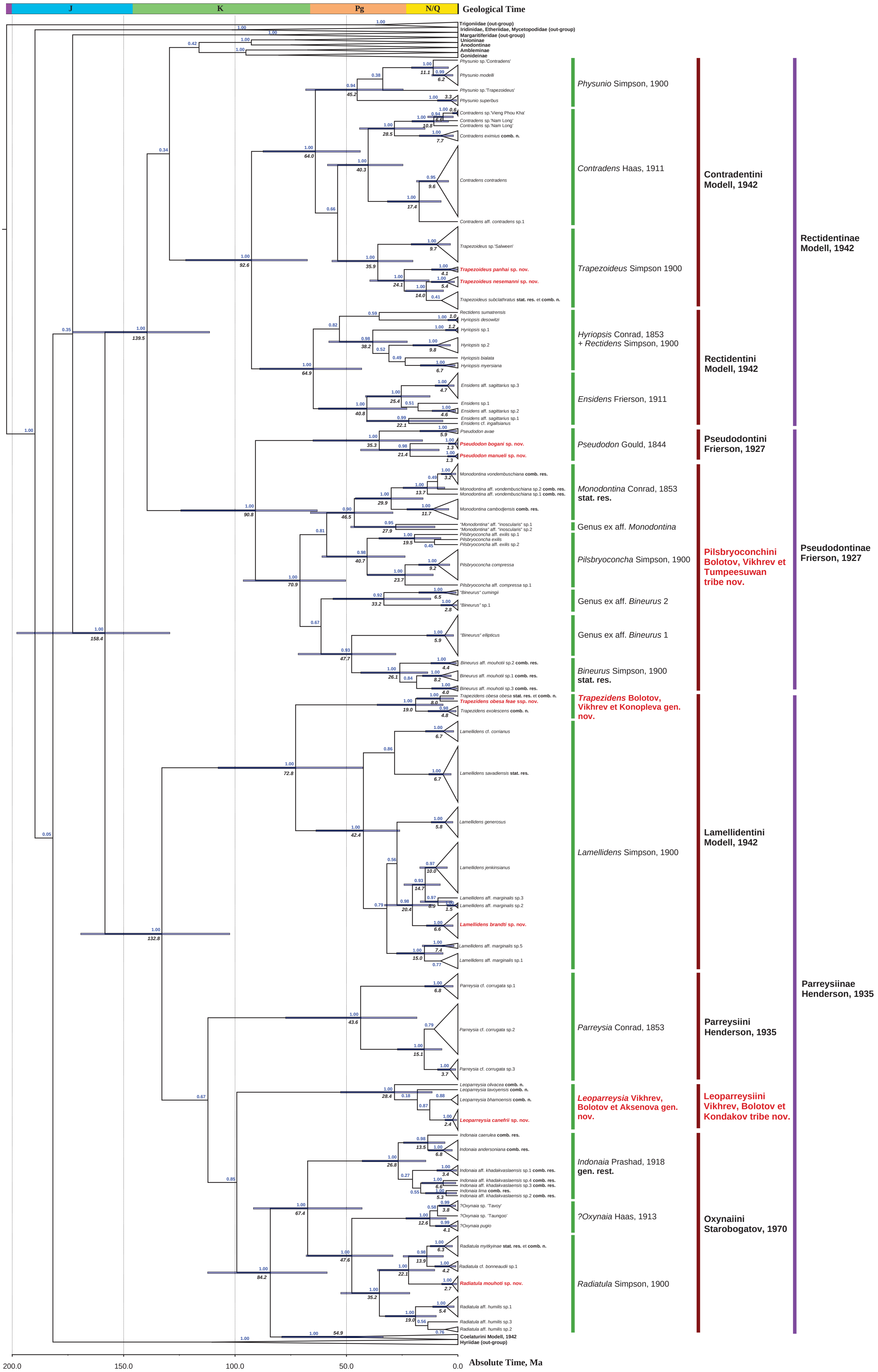

Supplementary Figure 2. Fossil-calibrated ultrametric chronogram calculated under a lognormal relaxed clock model and a Yule process speciation implemented in BEAST 1.8.4 and obtained for the complete data set of mitochondrial and nuclear sequences (five partitions: three codons of COI + 16S rRNA + 28S rDNA). The non-Indo-Chinese and out-group clades are collapsed. Black numbers near nodes are the mean age values, blue numbers near nodes are BPP values, and bars are 95% confidence intervals of the estimated divergence time between lineages (Ma). The timing of weakly supported nodes (BPP < 0.90) is omitted. Stratigraphic chart according to the International Commission on Stratigraphy, 2015. The list of sequences is presented in Supplementary Table 1.

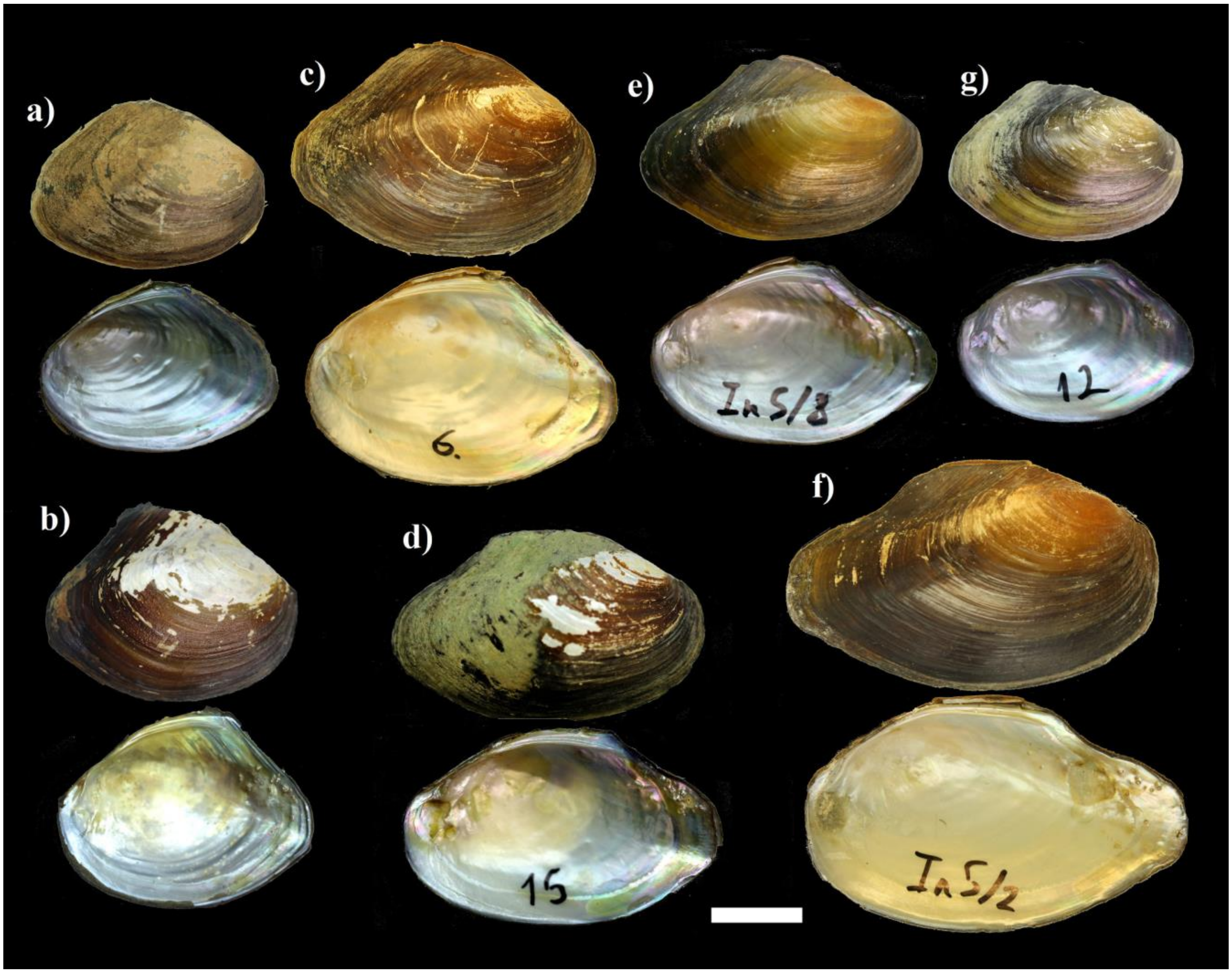

Supplementary Figure 3. Shell variability of *Lamellidens generosus* (Gould, 1847) from Lake Inle and surrounding water bodies. The corresponding nominal taxa are as follows: (**a-c**) *Physunio micropteroides* Annandale, 1918 **syn. nov.** (topotypes, specimen nos. RMBH biv142_1*, biv141_4, biv112_6, respectively), (**d-f**) *Physunio ferrugineus* Annandale, 1918 **syn. nov.** (topotypes, specimen nos. RMBH biv112_15*, biv113_8*, biv113_2, respectively), and (**g**) *Unio generosus* Gould, 1847 (specimen no. RMBH biv112_12*). An asterisk indicates sequenced specimens (Supplementary Table 1). Scale bar = 2 cm.

References

1. Rahel, F. J. Homogenization of freshwater faunas. *Annual Review of Ecology and Systematics* **33,** 291–315; DOI:10.1146/annurev.ecolsys.33.010802.150429 (2002).
2. Lydeard, C. *et al.* The global decline of nonmarine mollusks. *BioScience* **54,** 321–330; DOI:10.1641/0006-3568(2004)054[0321:TGDONM]2.0.CO;2 (2004).
3. Vörösmarty, C. J. *et al.* Global threats to human water security and river biodiversity. *Nature* **467,** 555–561; DOI:10.1038/nature09440 (2010).
4. Dirzo, R. *et al.* Defaunation in the Anthropocene. *Science* **345,** 401–406; DOI:10.1126/science.1251817 (2014).
5. Ceballos, G. *et al.* Accelerated modern human–induced species losses: Entering the sixth mass extinction. *Science Advances* **1,** e1400253; DOI:10.1126/sciadv.1400253 (2015).
6. McGill, B. J., Dornelas, M., Gotelli, N. J. & Magurran, A. E. Fifteen forms of biodiversity trend in the Anthropocene. *Trends in Ecology & Evolution* **30,** 104–113; DOI:10.1016/j.tree.2014.11.006 (2015).
7. Heino, J., Virkkala, R. & Toivonen, H. Climate change and freshwater biodiversity: detected patterns, future trends and adaptations in northern regions. *Biological Reviews* **84,** 39–54; DOI:10.1111/j.1469-185X.2008.00060.x (2009).
8. Wiens, J. J. Climate-related local extinctions are already widespread among plant and animal species. *PLoS Biology* **14,** e2001104; DOI:10.1371/journal.pbio.2001104 (2016).
9. Bolotov, I.N. *et al.* Ancient river inference explains exceptional Oriental freshwater mussel radiations. *Scientific Reports* **7,** 2135; DOI:10.1038/s41598-017-02312-z (2017).
10. Zieritz, A. *et al.* Diversity, biogeography and conservation of freshwater mussels (Bivalvia: Unionida) in East and Southeast Asia. *Hydrobiologia*, 1–16; DOI:10.1007/s10750-017-3104-8 (2017).
11. Tisseuil, C. *et al.* Global diversity patterns and cross-taxa convergence in freshwater systems. *Journal of Animal Ecology* **82,** 365–376; DOI:10.1111/1365-2656.12018 (2013).
12. Strong, E. E., Gargominy, O., Ponder, W. F. & Bouchet, P. Global diversity of gastropods (Gastropoda; Mollusca) in freshwater. *Hydrobiologia* **595,** 149–166; DOI:10.1007/978-1-4020-8259-7_17 (2008).
13. Bogan, A. E. Global diversity of freshwater mussels (Mollusca, Bivalvia) in freshwater. *Hydrobiologia* **595,** 139–147; DOI: 10.1007/s10750-007-9011-7 (2008).
14. Bogan, A. E. & Roe, K. J. Freshwater bivalve (Unioniformes) diversity, systematics, and evolution: status and future directions. *Journal of the North American Benthological Society* **27,** 349–369; DOI: 10.1899/07-069.1 (2008).
15. Graf, D. L. Patterns of freshwater bivalve global diversity and the state of phylogenetic studies on the Unionoida, Sphaeriidae, and Cyrenidae. *American Malacological Bulletin* **31,** 135–153; DOI:10.4003/006.031.0106 (2013).
16. Graf, D. L. & Cummings, K. S. Review of the systematics and global diversity of freshwater mussel species (Bivalvia: Unionoida). *Journal of Molluscan Studies* **73,** 291–314; DOI:10.1093/mollus/eym029 (2007).
17. Lopes-Lima, M. *et al.* Phylogeny of the most species-rich freshwater bivalve family (Bivalvia: Unionida: Unionidae): Defining modern subfamilies and tribes. *Molecular Phylogenetics and Evolution* **106,** 174–191; DOI:10.1016/j.ympev.2016.08.021 (2017).
18. Schneider, S., Böhme, M. & Prieto, J. Unionidae (Bivalvia; Palaeoheterodonta) from the Palaeogene of northern Vietnam: exploring the origins of the modern East Asian freshwater bivalve fauna. *Journal of Systematic Palaeontology* **11,** 337–357; DOI:10.1080/14772019.2012.665085 (2013).
19. Chowdhury, G. W., Zieritz, A. & Aldridge, D. C. Ecosystem engineering by mussels supports biodiversity and water clarity in a heavily polluted lake in Dhaka, Bangladesh. *Freshwater Science* **35,** 188–199; DOI:10.1086/684169 (2016).

20. Bolotov, I. N. *et al.* Spreading of the Chinese pond mussel, *Sinanodonta woodiana*, across Wallacea: One or more lineages invade tropical islands and Europe. *Biochemical Systematics and Ecology* **67,** 58–64; DOI:10.1016/j.bse.2016.05.018 (2016).

21. Zieritz, A. *et al.* Factors driving changes in freshwater mussel (Bivalvia, Unionida) diversity and distribution in Peninsular Malaysia. *Science of the Total Environment* **571,** 1069–1078; DOI:10.1016/j.scitotenv.2016.07.098 (2016).

22. Ng, T. H. *et al.* Molluscs for sale: assessment of freshwater gastropods and bivalves in the ornamental pet trade. *PloS ONE* **11,** e0161130; DOI:10.1371/journal.pone.0161130 (2016).

23. Bolotov, I. N. *et al.* Ecology and conservation of the endangered Indochinese freshwater pearl mussel, *Margaritifera laosensis* (Lea, 1863) in the Nam Pe and Nam Long rivers, Northern Laos. *Tropical Conservation Science* **7,** 706–719; DOI:10.1177/194008291400700409 (2014).

24. Subba Rao, N.V. *Handbook of freshwater molluscs of India* (Calcutta, 1989).

25. Konopleva E.S., Bolotov, I. N., Vikhrev I.V., Gofarov, M.Y. & Kondakov, A.V. An integrative approach underscores the taxonomic status of *Lamellidens exolescens*, a freshwater mussel from the Oriental tropics (Bivalvia: Unionidae). *Systematics and Biodiversity* **15,** 204–217; DOI:10.1080/14772000.2016.1249530 (2016).

26. Gould, A. A. Dr. Gould had examined the shells not long since announced as having been received from the Rev. Francis Mason, missionary at Tavoy, in British Burmah. *Proceedings of the Boston Society of Natural History* **1,** 139–141 (1843).

27. Gould, A. A. Dr. Gould read descriptions of two *Anodon*, from the river Salwen, in British Burmah, sent him by Rev. F. Mason. *Proceedings of the Boston Society of Natural History* **1,** 160–161 (1844).

28. Gould, A. A. Dr. Gould described new shells, received from Rev. Mr. Mason, of Burmah. *Proceedings of the Boston Society of Natural History* **2,** 218–221 (1847).

29. Mason, F. *Tenasserim: Or notes on the fauna, flora, minerals, and nations of British Burmah and Pegu: With systematic catalogues of the known minerals, plants, mammals, fishes, mollusks, sea-nettles, corals, sea-urchins, worms, insects, crabs, reptiles, and birds; with vernacular names* (Burma, Maulmain, 1851).

30. Theobald, W. Notes on the distribution of some of the land and freshwater shells of India, Part II. *Journal of the Asiatic Society of Bengal* **27,** 313–323 (1858).

31. Theobald, W. Notes on a collection of land and fresh-water shells from the Shan States. Collected by F. Fedden, Esq., 1864–65. *Journal of the Asiatic Society of Bengal* **34,** 273–279 (1865).

32. Theobald, W. Descriptions of new species of Unionidae. *Journal of the Asiatic Society of Bengal* **43,** 207 (1873).

33. Blanford, W. T. Contributions of Indian Malacology, No. VII. List of species of *Unio* and *Anodonta* described as occurring in India, Ceylon, and Burma. *Journal of the Asiatic Society of Bengal* **35,** 134–155 (1866).

34. Hanley, S. Description of new land and freshwater shells from India. *Proceedings of the Zoological Society of London* **1875,** 605–607 (1875).

35. Hanley, S. & Theobald, W. *Conchologia Indica: Illustrations of the Land and Freshwater Shells of British India* (London, 1876).

36. Nevill G. Mollusca brought by Dr. J. Anderson from Yunan and Upper Burma, with descriptions of new species. *Journal of the Asiatic Society of Bengal* **46,** 14–41 (1877).

37. Martens, E. v. Binnen-Conchylien aus Ober-Birma. *Archiv für Naturgeschichte* **65,** 30–48 (1899).

38. Tapparone-Canefri, C. Viaggio de Leonardo Fea in Birmania e regioni vicine. XVIII. Molluschi terrestri e d'acqua dolce. *Annali del Museo Civico di Storia Naturale de Genova (series 2)* **27,** 295–359 (1889).

39. Simpson, C.T. Synopsis of the naiades, or pearly fresh-water mussels. *Proceedings of the United States National Museum* **22,** 501–1044 (1900).

40. Simpson, C.T. A descriptive catalogue of the naiades, or pearly fresh-water mussels (Parts I-III). (Detroit, 1914).

41. Preston, H.B. A catalogue of the Asiatic naiades in the collection of the Indian Museum, Calcutta, with descriptions of new species. *Records of the Indian Museum* **7,** 279–308 (1912).

42. Annandale, N. Aquatic molluscs of the Inlé Lake and connected waters. *Records of the Indian Museum* **14,** 103–182 (1918).

43. Prashad, B. A revision of the Burmese Unionidae. *Records of the Indian Museum* **24,** 91–111 (1922).

44. Prashad, B. Pelecypoda of the Indawgyi Lake and of its connected freshwater areas in the Myitkyina District, Upper Burma. *Records of the Indian Museum* **32,** 247–255 (1930).

45. Haas, F. Superfamilia Unionacea. *Das Tierreich* **88,** 1–663 (1969).

46. Benson, W.H. Descriptions of Indian and Burmese species of the genus *Unio*, Retz. *Annals and Magazine of Natural History (Third Series)* **10,** 184–195 (1862).

47. Anthony, J.G. Descriptions of two new species of *Monocondylaea*. *American Journal of Conchology* **1,** 205–206 (1865).

48. Prashad, B. Notes on Lamellibranchs in the Indian Museum. *Records of the Indian Museum* **19,** 165–173 (1920).

49. Fea, L. Nei Carin Indipendenti. *Estratto dal Bollettino della Società Geografica Italiana* **1,** 1–15 (1888).

50. Haas F. Genus *Margaritinopsis* Haas 1912. *Martini und Chemnitz, Systematisches Conchyliencabinet* **9,** 121–123 (1912).

51. Brandt, R. A. M. (1974). The non-marine aquatic mollusca of Thailand. *Archiv für Molluscкenkunde* **105,** 1–423.

52. Nesemann, H. A., Sharma, S. U., Sharma, G. O. & Sinha, R. K. Illustrated checklist of large freshwater bivalves of the Ganga river system (Mollusca: Bivalvia: Solecurtidae, Unionidae, Amblemidae). *Nachrichchtenblatt der Ersten Vorarlberger Malakologischen Gesellschaft* **13,** 1–51 (2005).

53. Whelan, N. V., Geneva, A. J. & Graf, D. L. Molecular phylogenetic analysis of tropical freshwater mussels (Mollusca: Bivalvia: Unionoida) resolves the position of *Coelatura* and supports a monophyletic Unionidae. *Molecular Phylogenetics and Evolution* **61,** 504–514; DOI:10.1016/j.ympev.2011.07.016 (2011).

54. Pfeiffer, J. M. III & Graf. D. L. Evolution of bilaterally asymmetrical larvae in freshwater mussels (Bivalvia: Unionoida: Unionidae). *Zoological Journal of the Linnean Society* **175,** 307–318; DOI: 10.1111/zoj.12282 (2015).

55. Frierson, L.S. *A Classified and Annotated Check List of the North American Naiades* (Waco, Texas, 1927).

56. Clark, M. K. *et al.* Late Cenozoic uplift of southeastern Tibet. *Geology* **33,** 525–528; DOI:10.1130/G21265.1 (2005).

57. Wang, M., Yang, J.-X., Chen, X.-Y. Molecular phylogeny and biogeography of *Percocypris* (Cyprinidae, Teleostei). *PLoS ONE* **8,** e61827; DOI:10.1371/journal.pone.0061827 (2013).

58. Köhler, F. *et al.* The status and distribution of freshwater molluscs of the Indo-Burma region. in The status and distribution of freshwater biodiversity in Indo-Burma, 66–88 (2012).

59. Graf, D. L. & Cummings, K. S. The freshwater mussels (Unionoida) of the World (and other less consequential bivalves), updated 5 August 2015. MUSSEL Project Web Site. Available: http://www.mussel-project.net (2015).

60. Hall, T. A. BioEdit: a user-friendly biological sequence alignment editor and analysis program for Windows 95/98/NT. *Nucleic Acids Symposium Series* **41,** 95–98 (1999).

61. Tamura, K., Stecher, G., Peterson, D., Filipski, A. & Kumar, S., MEGA6: Molecular Evolutionary Genetics Analysis version 6.0. *Molecular Biology and Evolution* **30,** 2725–2729; DOI:10.1093/molbev/mst197 (2013).

62. Talavera, G. & Castresana, J. Improvement of phylogenies after removing divergent and ambiguously aligned blocks from protein sequence alignments. *Systematic Biology* **56,** 564–577; DOI:10.1080/10635150701472164 (2007).
63. Xia, X., Xie, Z., Salemi, M., Chen, L. & Wang Y. An index of substitution saturation and its application. *Molecular Phylogenetics and Evolution* **26,** 1–7; DOI:10.1016/S1055-7903(02)00326-3 (2003).
64. Xia, X. & Lemey, P. Assessing substitution saturation with DAMBE. in The Phylogenetic Handbook: A Practical Approach to DNA and Protein Phylogeny, Second Edition (Lemey, P., Salemi, M. & Vandamme, A., eds.) 615–630 (Cambridge University Press, 2009).
65. Swofford, D.L. PAUP*. Phylogenetic Analysis Using Parsimony (*and Other Methods). Version 4.0b10. (Sinauer Associates, Sunderland, Massachusetts, 2002).
66. Villesen, P. FaBox: an online toolbox for fasta sequences. *Molecular Ecology Notes* **7,** 965–968; DOI:10.1111/j.1471-8286.2007.01821.x (2007).
67. Stamatakis, A. RAxML-VI-HPC: maximum likelihood-based phylogenetic analyses with thousands of taxa and mixed models. *Bioinformatics* **22,** 2688–2690; DOI:10.1093/bioinformatics/btl446 (2006).
68. Miller, M., Pfeiffer, W. & Schwartz, T. Creating the CIPRES Science Gateway for inference of large phylogenetic trees. in Gateway Computing Environments Workshop (GCE). 1–8 (IEEE, 2010).
69. Ronquist, F. *et al.* MrBayes 3.2: Efficient Bayesian Phylogenetic Inference and Model Choice Across a Large Model Space. *Systematic Biology* **61,** 539–542; DOI:10.1093/sysbio/sys029 (2012).
70. Rambaut, A., Suchard, M. & Drummond, A. J. Tracer v1.6. Available: http://beast.bio.ed.ac.uk/software/tracer/ (2013).
71. Drummond, A. J., Ho, S. Y., Phillips, M. J. & Rambaut, A. Relaxed phylogenetics and dating with confidence. *PLoS Biology* **4,** 699; DOI:10.1371/journal.pbio.0040088 (2006).
72. Drummond, A.J. & Rambaut, A. BEAST: Bayesian evolutionary analysis by sampling trees. *BMC Evolutionary Biology* **7,** 214; DOI:10.1186/1471-2148-7-214 (2007).
73. Drummond, A. J., Suchard, M. A., Xie, D. & Rambaut, A. Bayesian phylogenetics with BEAUti and the BEAST 1.7. *Molecular Biology and Evolution* **29,** 1969–1973; DOI:10.1093/molbev/mss075 (2012).
74. Blaxter, M. *et al.* Defining operational taxonomic units using DNA barcode data. *Philosophical Transactions of the Royal Society B: Biological Sciences* **360,** 1935–1943; DOI:10.1098/rstb.2005.1725 (2005).
75. De Queiroz, K. Species concepts and species delimitation. *Systematic Biology* **56,** 879–886; DOI:10.1080/10635150701701083 (2007).
76. Jones, M., Ghoorah, A. & Blaxter, M. jMOTU and Taxonerator: Turning DNA barcode sequences into annotated operational taxonomic units. *PLoS ONE* **6,** e19259; DOI:10.1371/journal.pone.0019259 (2011).
77. Wiens, J. J. Species delimitation: new approaches for discovering diversity. *Systematic Biology* **56,** 875–878; DOI:10.1080/10635150701748506 (2007).
78. Zhang, J., Kapli, P., Pavlidis, P. & Stamatakis, A. A general species delimitation method with applications to phylogenetic placements. *Bioinformatics* **29,** 2869–2876; DOI:10.1093/bioinformatics/btt499 (2013).
79. Kapli, P. *et al.* Multi-rate Poisson tree processes for single-locus species delimitation under maximum likelihood and Markov chain Monte Carlo. *Bioinformatics* **33,** 1630–1638; DOI:10.1093/bioinformatics/btx025 (2017).
80. Delić, T., Trontelj, P., Rendoš, M. & Fišer, C. The importance of naming cryptic species and the conservation of endemic subterranean amphipods. *Scientific Reports* **7,** 3391; DOI:10.1038/s41598-017-02938-z (2017).
81. Renner, S. S. A return to Linnaeus's focus on diagnosis, not description: The use of DNA characters in the formal naming of species. *Systematic Biology* **65,** 1086–1095; DOI:10.1093/sysbio/syw032 (2016).

82. Jörger, K. M. & Schrödl, M. How to describe a cryptic species? Practical challenges of molecular taxonomy. *Frontiers in Zoology* **10,** 59; DOI:10.1186/1742-9994-10-59 (2013).
83. Anthony, J.G. Descriptions of new species of shells. *American Journal of Conchology* **1,** 351 (1865).
84. Sowerby, G.B. Genus *Unio*. *Conchologica Iconica* **16,** 61–96 (1868).
85. Graf, D. L., Geneva, A. J., Pfeiffer, J. M. & Chilala, A. D. Phylogenetic analysis of *Prisodontopsis* Tomlin, 1928 and *Mweruella* Haas, 1936 (Bivalvia: Unionidae) from Lake Mweru (Congo basin) supports a Quaternary radiation in the Zambian Congo. *Journal of Molluscan Studies* **80,** 303–314; DOI:10.1093/mollus/eyu012 (2014).
86. Graf, D. L. Molecular phylogenetic analysis of two problematic freshwater mussel genera (*Unio* and *Gonidea*) and a re-evaluation of the classification of Nearctic Unionidae (Bivalvia: Palaeoheterodonta: Unionoida). *Journal of Molluscan Studies* **68,** 65–71; DOI:10.1093/mollus/68.1.65 (2002).
87. Graf, D. L. & Cummings, K. S. Palaeoheterodont diversity (Mollusca: Trigonioida + Unionoida): what we know and what we wish we knew about freshwater mussel evolution. *Zoological Journal of the Linnean Society* **148,** 343–394; DOI:10.1111/j.1096-3642.2006.00259.x (2006).
88. Prié, V. & Puillandre, N. Molecular phylogeny, taxonomy, and distribution of French *Unio* species (Bivalvia, Unionidae). *Hydrobiologia* **735,** 95–110; DOI:10.1007/s10750-013-1571-0 (2014).
89. Sharma, P. P. *et al.* Into the deep: a phylogenetic approach to the bivalve subclass Protobranchia. *Molecular Phylogenetics and Evolution* **69,** 188–204; DOI:10.1016/j.ympev.2013.05.018 (2013).
90. Graf, D. L. & Foighil, D. Ó. The evolution of brooding characters among the freshwater pearly mussels (Bivalvia: Unionoidea) of North America. *Journal of Molluscan Studies* **66,** 157–170; DOI:10.1093/mollus/66.2.157 (2000).
91. Bogan, A. E. & Hoeh, W. R. On becoming cemented: evolutionary relationships among the genera in the freshwater bivalve family Etheriidae (Bivalvia: Unionoida). *Geological Society of London, Special Publications* **177,** 159–168; DOI:10.1144/GSL.SP.2000.177.01.09 (2000).
92. Froufe, E. *et al.* Phylogeny, phylogeography, and evolution in the Mediterranean region: News from a freshwater mussel (*Potomida*, Unionida). *Molecular Phylogenetics and Evolution* **100,** 322–332; DOI:10.1016/j.ympev.2016.04.030 (2016).
93. Gustafson, R. G. & Iwamoto, E. M. A DNA-based identification key to Pacific Northwest freshwater mussel glochidia: importance to salmonid and mussel conservation. *Northwest Science* **79,** 233–245 (2005).
94. Hoeh, W. R. *et al.* Testing altenative hypotheses of *Neotrigonia* (Bivalvia: Trigonioida) phylogenetic relationships using cytochrome c oxidase subunit I DNA sequences. *Malacologia* **40,** 267–278 (1998).
95. Giribet, G. & Wheeler, W. On bivalve phylogeny: a high-level analysis of the Bivalvia (Mollusca) based on combined morphology and DNA sequence data. *Invertebrate Biology* **121,** 271–324; DOI:10.1111/j.1744-7410.2002.tb00132.x (2002).
96. Distel, D. L. *et al.* Molecular phylogeny of Pholadoidea Lamarck, 1809 supports a single origin for xylotrophy (wood feeding) and xylotrophic bacterial endosymbiosis in Bivalvia. *Molecular Phylogenetics and Evolution* **61,** 245–254; DOI:10.1016/j.ympev.2011.05.019 (2011).
97. Bolotov, I. N. *et al.* Multi-locus fossil-calibrated phylogeny, biogeography and a subgeneric revision of the Margaritiferidae (Mollusca: Bivalvia: Unionoida). *Molecular Phylogenetics and Evolution* **103,** 104–121; DOI:10.1016/j.ympev.2016.07.020 (2016).
98. Huff, S.W. *et al.* Investigations into the phylogenetic relationships of freshwater pearl mussels (Bivalvia: Margaritiferidae) based on molecular data: implications for their taxonomy and biogeography. *Journal of Molluscan Studies* **70,** 379–388; DOI:10.1093/mollus/70.4.379 (2004).

99. Araújo, R., Toledo, C., Van Damme, D., Ghamizi, M. & Machordom, A. *Margaritifera marocana* (Pallary, 1918): a valid species inhabiting Moroccan rivers. *Journal of Molluscan Studies* **75,** 95–101; DOI:10.1093/mollus/eyn043 (2009).

100. Breton, S. *et al.* Novel protein genes in animal mtDNA: a new sex determination system in freshwater mussels (Bivalvia: Unionoida)? *Molecular Biology and Evolution* **28,** 1645–1659; DOI:10.1093/molbev/msq345 (2011).

101. González, V. L. & Giribet, G. A multilocus phylogeny of archiheterodont bivalves (Mollusca, Bivalvia, Archiheterodonta). *Zoologica Scripta* **44,** 41–58; DOI:10.1111/zsc.12086 (2015).

102. Graf, D. L., Jones, H., Geneva, A. J., Pfeiffer, J. M. & Klunzinger, M. W. Molecular phylogenetic analysis supports a Gondwanan origin of the Hyriidae (Mollusca: Bivalvia: Unionida) and the paraphyly of Australasian taxa. *Molecular Phylogenetics and Evolution* **85,** 1–9; DOI:10.1016/j.ympev.2015.01.012 (2015).

103. Giribet, G. *et al.* Evidence for a clade composed of molluscs with serially repeated structures: monoplacophorans are related to chitons. *Proceedings of the National Academy of Sciences* **103,** 7723–7728; DOI:10.1073/pnas.0602578103 (2006).

104. Lobo, J. *et al.* Enhanced primers for amplification of DNA barcodes from a broad range of marine metazoans. BMC Ecology **13,** 34; DOI:10.1186/1472-6785-13-34 (2013).

105. Palumbi, S.R. Nucleic acids II: The polymerase chain reaction. in Molecular Systematics (Hillis D.M., Moritz C., Mable B.K., eds.). 205–247 (Sinauer Associates Inc., Sunderland, MA, 1996).

106. Lydeard, C., Mulvey, M., Davis, G. M. Molecular systematics and evolution of reproductive traits of North American freshwater unionacean mussels (Mollusca: Bivalvia) as inferred from 16S rRNA gene sequences. *Philosophical Transactions of the Royal Society B: Biological Sciences* **351,** 1593–1603; DOI:10.1098/rstb.1996.0143 (1996).

107. Jovelin, R. & Justine, J. L. Phylogenetic relationships within the polyopisthocotylean monogeneans (Platyhelminthes) inferred from partial 28S rDNA sequences. *International Journal for Parasitology* **31,** 393–401; DOI:10.1016/S0020-7519(01)00114-X (2001).
